# Gut, Oral, and Fungal Microbiota in Hypertension: A Multi-Compartment Systematic Review

**DOI:** 10.3390/ijms27188029

**Published:** 2026-09-09

**Authors:** Francesco Carini, Alessandra Sorce, Maria Elena Ciuppa, Sabrina David, Marco Giammanco, Paola Di Carlo, Salvatore Evola, Giovanni Tomasello, Giuseppe Mulè, Caterina Carollo

**Affiliations:** 1 Department of Biomedicine, Neurosciences and Advanced Diagnostics (BIND), Institute of Human Anatomy and Histology, University of Palermo, 90127 Palermo, Italy; francesco.carini@unipa.it (F.C.); giovanni.tomasello@unipa.it (G.T.); 2 Department of Health Promotion, Mother and Child Care, Internal and Specialistic Medicine, University of Palermo, 90127 Palermo, Italy; alessandra.sorce@community.unipa.it (A.S.); mariaelena.ciuppa@community.unipa.it (M.E.C.); paola.dicarlo@unipa.it (P.D.C.); giuseppe.mule@unipa.it (G.M.); 3 Department of Precision Medicine in the Medical, Surgical, and Intensive Care Fields, University of Palermo, 90127 Palermo, Italy; sabrina.david@unipa.it (S.D.); marco.giammanco@unipa.it (M.G.); 4 Catheterization Laboratory, Department of Medicine and Cardiology, Azienda Ospedaliera Universitaria Policlinico “P. Giaccone”, 90127 Palermo, Italy; salvatore.evola@policlinico.pa.it

**Keywords:** hypertension, blood pressure, gut microbiome, oral microbiome, mycobiome, systematic review, SWiM, nitric oxide, *Ruminococcus gnavus*, *Neisseria subflava*

## Abstract

The gut microbiota is an established modulator of blood pressure, but the oral bacteriome and the fungal mycobiome have been examined largely in isolation from it and from each other. No previous synthesis has evaluated all three compartments within one analytical framework, or treated sex and ethnicity as primary analytical axes rather than adjustment covariates. Systematic review reported according to PRISMA 2020 and, for the synthesis, the SWiM guideline. PubMed/MEDLINE, Embase, Scopus, and Web of Science were searched from inception to 30 June 2026. Observational human studies in adults reporting gut, oral, or fungal microbiota data stratified by blood pressure status were eligible, together with Mendelian randomisation studies and studies with a nested experimental causal component. Two reviewers screened and extracted independently, with a third resolving disagreement. Risk of bias was assessed with the Newcastle–Ottawa Scale and certainty of evidence with GRADE adapted for exposure–outcome questions. Increased abundance of the *Ruminococcus gnavus* group was the most convergent taxon-level finding, replicated in three independent populations on two continents, including one prospective multi-ethnic cohort with full adjustment and correction for multiple comparisons (OR 1.07, 95% CI 1.01–1.14 for incident hypertension). In the oral compartment, depletion of the nitrate-reducing commensal *Neisseria subflava* converged across a United States prospective cohort and an Italian case–control study using unrelated methods, and salivary nitric oxide was approximately three-fold lower in hypertensive subjects. Depletion of the short-chain fatty acid producers Faecalibacterium and Roseburia and enrichment of Klebsiella were convergent but geographically restricted. Mycobiome evidence was contradictory: two studies reported fungal dysbiosis, one of them already at the pre-hypertensive stage, while a cross-cohort metagenome-wide study on two independent cohorts from Beijing and Dalian (N = 159 hypertensive patients, 101 healthy controls) identified 61 gut bacterial species with consistent altered abundance across both cohorts while finding no replicable mycobiome signal. Recurring across compartments and kingdoms was the collapse of microbial co-correlation networks in hypertension, alongside a dissociation between null alpha diversity and significant beta diversity. Associations differed by ethnicity within a single multi-ethnic cohort and were generally stronger in women. Certainty of evidence, assessed per individual convergent finding, was very low for every taxon-level finding and low for salivary nitric oxide; these ratings concern the attribution of hypertension to specific organisms, not the existence of a microbiota–hypertension association, which is supported at community level in every compartment examined and by experimental transfer models. That the microbiota differs in hypertension is well supported; which organisms are responsible is not. The most reproducible signal is structural rather than taxonomic, and conventional differential-abundance analysis is not designed to detect it. No individual microbial taxon is currently ready to serve as a marker of hypertension or to inform clinical practice.

## 1. Introduction

Hypertension is one of the leading modifiable risk factors for cardiovascular morbidity and mortality worldwide. Between 1990 and 2019, the number of adults aged 30–79 living with this condition doubled from 648 million to 1.28 billion; over half remain undiagnosed, and fewer than one in four achieve adequate blood pressure control [1]. It stands as the single largest contributor to the global burden of cardiovascular disease and premature death [2].

Diagnostic thresholds, however, vary across clinical guidelines: the 2017 ACC/AHA criteria classify hypertension starting at 130/80 mmHg, whereas WHO and European guidelines maintain the traditional 140/90 mmHg cutoff [3]. This divergence significantly alters both prevalence estimates and cohort composition in observational research. Despite decades of study into classic determinants—genetic, dietary, behavioural, and pharmacological—a substantial proportion of interindividual variability in blood pressure regulation remains unexplained [2]. This gap has fuelled growing interest in emerging environmental drivers, particularly the human microbiota.

Lately, converging evidence from animal models, human observational cohorts and causal-inference designs has established the gut microbiota as an active modulator of blood pressure. The landmark study by Li et al. (2017) [4] provided the first direct evidence of causality by demonstrating that faecal microbiota transplantation from hypertensive patients into germ-free mice was sufficient to induce elevated blood pressure in the recipients—a finding subsequently reinforced by Mendelian randomisation analyses [5].

**Figure 1:**
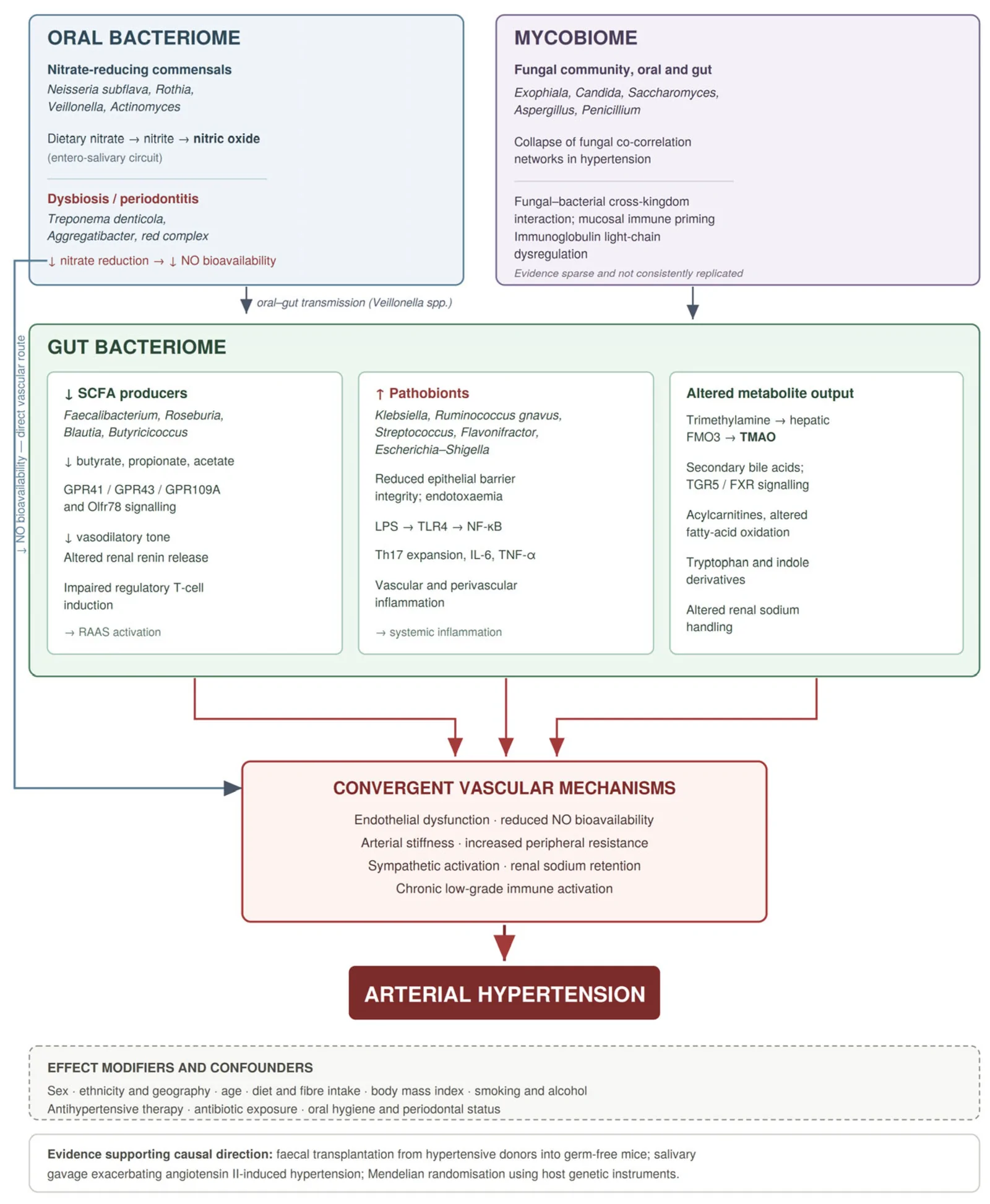
Proposed pathophysiological framework linking the human microbiota to blood pressure regulation across three compartments. Oral nitrate-reducing commensals sustain the entero-salivary nitrate–nitrite–nitric oxide circuit and act on the vasculature directly; the gut bacteriome acts through short-chain fatty acid signalling, epithelial barrier integrity and endotoxaemia, and altered metabolite output; the mycobiome is the least characterised compartment and its contribution remains provisional. Oral–gut microbial transmission connects the first two. The three routes converge on a common set of vascular mechanisms. The arrow direction indicates proposed mechanistic flow, not established causality: the causal evidence available in humans is limited to the designs listed at the foot of the figure.

Several key physiological pathways underpin this connection. Short-chain fatty acids (SCFAs), produced through bacterial fermentation of dietary fibre, modulate vascular tone and renal renin secretion via GPR41, GPR43, GPR109A, and Olfr78 receptors [6]. In experimental models, gut dysbiosis accompanies neurohormonal alterations and immune activation within hypertensive phenotypes [7]. Furthermore, intestinal barrier breakdown leads to bacterial translocation and endotoxaemia in hypertensive patients [8]. Concurrently, circulating microbial metabolites such as trimethylamine N-oxide (TMAO) [9] track with incident cardiovascular events [10,11], post-infarction and stroke prognosis [12], and blood pressure levels in a dose-dependent manner among patients with established cardiovascular disease [13]. Specific to the oral cavity, commensal nitrate-reducing bacteria convert dietary nitrate to nitrite and subsequently nitric oxide (NO)—a metabolic pathway the human host cannot execute independently [14], and whose impairment directly disrupts blood pressure regulation [15]. Physiological pathways are illustrated in Figure 1.

Despite these insights, research has focused heavily on the gut bacteriome, leaving two other critical compartments largely unexplored. The oral bacteriome, operating primarily through the nitrate–nitrite–NO circuit, was only recently evaluated in a rigorous prospective study [16], a case–control design with direct biochemical assays [17], and work demonstrating oral–gut microbial transmission [18]. Knowledge of the mycobiome remains even more rudimentary, limited mostly to isolated gut studies [19] or recent multi-compartment work exploring fungal correlations between the mouth and gut [20]. Conversely, a recent cross-cohort analysis published after this corpus was assembled reports consistent bacterial shifts alongside an essentially unchanged mycobiome [21]. This fragmented landscape has prevented an integrated synthesis capable of weighing the relative contributions and crosstalk among all three compartments within a single framework.

Existing syntheses are limited in scope. Avram et al. [22] conducted a meta-analysis restricted solely to the gut; Al-Maweri et al. [23] qualitatively reviewed the oral compartment without cross-referencing gut-level data; and an earlier systematic review of observational gut studies [24] predates most prospective evidence available today. Furthermore, sex and ethnicity are almost universally treated as confounding variables rather than core analytical dimensions. This overlooks the marked variation observed across ethnic groups in the multi-ethnic HELIUS cohort [25,26] and between sexes, such as in the female-only discovery–replication design of TwinsUK/PREDICT-1 [27]. Meanwhile, the mycobiome is rarely granted more than a brief narrative mention.

This systematic review integrates evidence across the gut bacteriome, oral bacteriome, and mycobiome within a unified multi-compartment, multi-kingdom framework, positioned in direct dialogue with—and clear distinction from—Avram et al. [22] and Al-Maweri et al. [23]. Four key methodological choices define its approach. First, it synthesizes all three compartments simultaneously to uncover cross-talk patterns that remain hidden when studying anatomical sites in isolation. Second, it elevates sex and ethnicity to core analytical dimensions rather than adjusting for them as covariates, leveraging cohorts specifically built for this purpose, such as female-only cohorts with independent replication and formally stratified multi-ethnic cohorts. Third, it explicitly separates causal-inference studies—including faecal transplantation in germ-free models, Mendelian randomisation, and experimental oral–gut transmission—from purely observational data. Finally, it opts for a narrative synthesis because the primary literature lacks the minimum of ten homogeneous, directly comparable studies required for a methodologically sound pooled estimate, retaining secondary syntheses strictly as comparative benchmarks.

## 2. Methods

This systematic review was conducted and reported according to PRISMA 2020 [28] and, for the synthesis, the Synthesis Without Meta-analysis (SWiM) guideline [29].

The review protocol was not prospectively registered in a public database (e.g., PROSPERO), as this study was undertaken under tight project timelines as a preparatory evidence-mapping phase to establish the methodological basis for a planned future meta-analysis. However, all eligibility criteria, literature search strategies, and analytical approaches were strictly pre-specified prior to data collection to ensure methodological transparency and minimize reporting bias.

Eligible studies were observational (case–control, cross-sectional, prospective cohort) in adults (≥18 years) of either sex, without restriction by ethnicity or region, characterising the gut bacteriome, oral bacteriome, and/or mycobiome by 16S rRNA sequencing, shotgun metagenomics, or targeted qPCR, and reporting these data stratified by blood pressure status. Comparators were normotensive subjects, or absence of hypertension at baseline in prospective designs. Hypertension was defined by standard criteria (systolic BP ≥140 mmHg and/or diastolic BP ≥90 mmHg and/or antihypertensive therapy), with study-specific threshold variation recorded. Secondary outcomes were alpha and beta diversity, relative taxon abundance, and major adverse cardiovascular events where reported. Mendelian randomisation studies, and studies with an experimental causal component nested within a human-population study, were also eligible. Excluded were secondary syntheses, retained instead as narrative benchmarks; paediatric populations; animal-only studies; studies without blood-pressure-stratified data; and letters, editorials, case reports and abstracts without full text. Reports sharing a cohort with an included study were retained where they contributed non-overlapping data, annotated as an additional compartment of that population rather than as an independent unit for the study count or for convergence counting.

**Figure 2:**
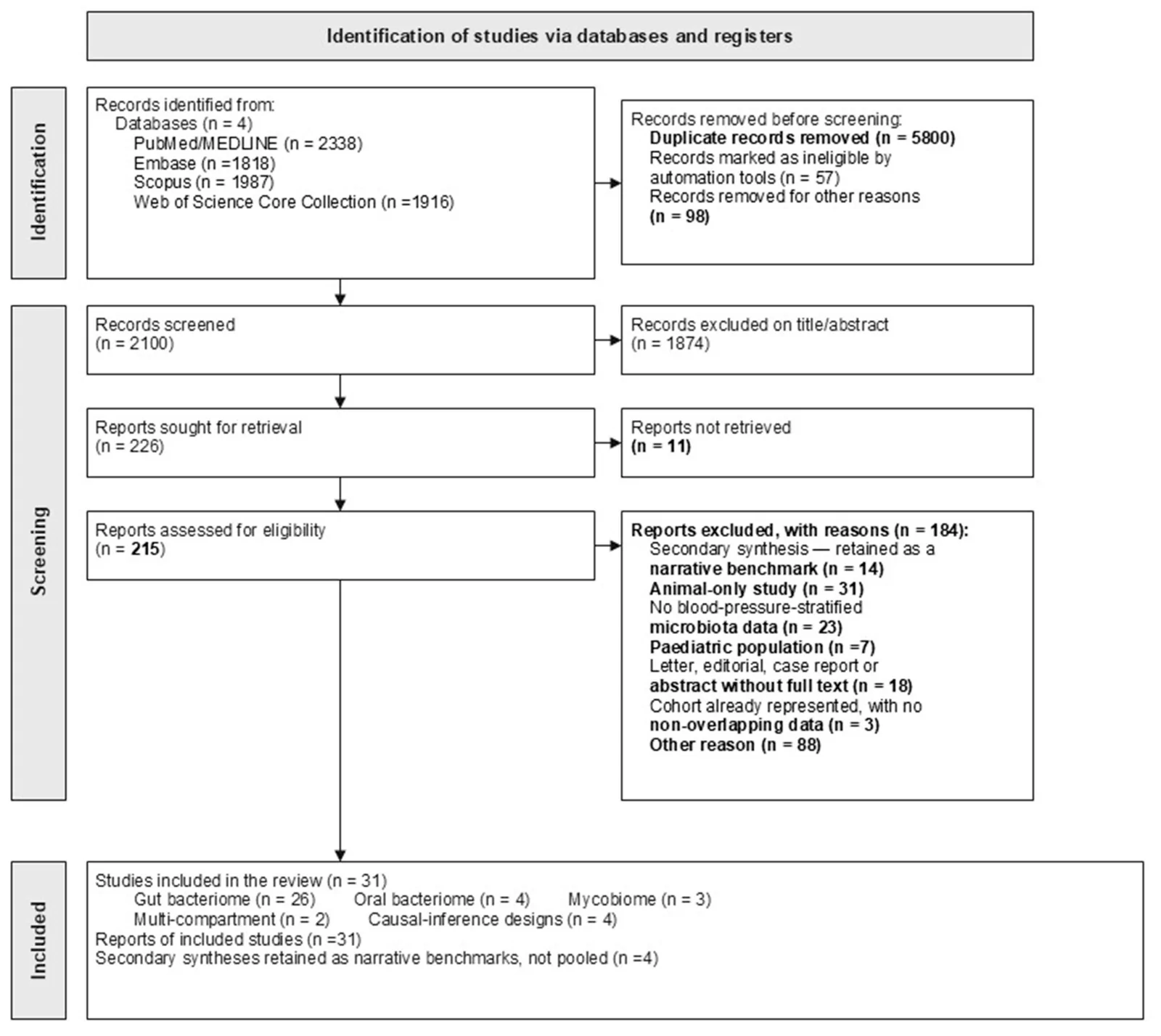
PRISMA 2020 flow diagram of study identification, screening and inclusion. Two of the counts in the screening stage (reports not retrieved; reports excluded at full text with reasons) are being reconciled against the original screening log and are shown as placeholders pending confirmation; all other counts, including the final total of 31 included studies, are verified. Template adapted from [28].

The search was run in June 2026 in PubMed/MEDLINE, Embase, Scopus, and the Web of Science Core Collection, from inception to 30 June 2026, without language restriction. It combined two concept blocks—microbiota and hypertension—using controlled vocabulary and free-text terms, kept deliberately broad at compartment level so that gut, oral, and fungal studies were retrieved by one strategy. Full strings and record counts per database are in Supplementary Table S1. After deduplication, two reviewers independently screened titles and abstracts and independently assessed full texts against the criteria above, with a third reviewer resolving disagreements at either stage. Reference lists of the included studies and of the benchmark syntheses were screened as a second search arm. Record flow is shown in Figure 2; the corresponding PRISMA 2020 checklist is provided as Supplementary Checklist S1 and the SWiM reporting checklist as Supplementary Checklist S2.

Two reviewers independently extracted study and population characteristics, hypertension definition, microbiota methodology and pipeline, diversity and taxon-level results with effect estimates and confidence intervals, correction for multiple comparisons, functional and metabolic data, and confounder adjustment; discrepancies were reconciled against the source and, if persistent, resolved by the third reviewer. The extraction template is provided as Supplementary Form S1. Included studies span publication years 2017 to 2026.

Risk of bias was assessed with the Newcastle–Ottawa Scale in the version matching each design, using the adaptation of Modesti et al. [30] for cross-sectional studies. Per-study judgements for each design are reported in Supplementary Tables S2a–d.

Findings were grouped by compartment and, within compartment, by type of result. Diversity measures were tabulated as reported by the original authors and compared narratively, without a summary estimate. Taxon-level results were synthesised by structured vote-counting on direction of effect, aggregated at phylogenetic family level given the number of taxa tested per study and the low overlap between studies; a taxon was considered convergent when reported in a concordant direction by at least two independent studies not sharing a cohort. Findings were further examined by sex and ethnicity, distinguishing studies purposively designed for an axis from post-hoc subgroup analyses, and studies capable of supporting causal inference were synthesised separately from associative ones. Studies whose comparator was not normotensive were retained and addressed in dedicated sub-analyses.

No quantitative pooling was performed. The decision follows from heterogeneity rather than from any threshold number of studies: the available estimates differ in compartment, in whether the outcome is prevalent or incident hypertension, in whether blood pressure is treated as a dichotomy or as a continuous variable, and in the metric reported—beta coefficient, odds ratio or hazard ratio—and they rest on studies that differ in case definition, in blood-pressure ascertainment and in the confounders adjusted (Tables S3 and S4). A summary estimate drawn across these differences would convey a precision the underlying data do not possess; the Cochrane Handbook’s guidance on when meta-analysis is appropriate turns on this question of clinical and methodological comparability rather than on a count of studies [31]. Sparse data compound the problem, as for most outcomes only one or two studies report a comparable estimate, but scarcity is a consequence of the heterogeneity rather than the reason for the decision. This is an explicit scoping decision and a declared point of differentiation from the meta-analysis of Avram et al. [22].

Certainty of evidence was assessed with GRADE, adapted for exposure–outcome association rather than intervention effect and applied per key convergent finding. The three rating-up criteria were considered for every finding and applied where met. Judgements are reported in Section 3.4.

## 3. Results

### 3.1. The Oral Bacteriome and Oral-Gut Axis

#### 3.1.1. Overview of Included Studies

The oral compartment is represented by four primary studies plus one narratively retained secondary review. LaMonte et al. [16] is the corpus’s only prospective study of the oral microbiome, following 735 postmenopausal women (1215 at baseline) from the Women’s Health Initiative for a mean of 10.4 years for incident hypertension, with subgingival plaque profiled by 16S rRNA sequencing. Barbadoro et al. [17] is a case–control study of 48 subjects (25 normotensive, 23 hypertensive) using targeted qPCR quantification of eight species plus total bacterial count in saliva and supragingival/subgingival/denture plaque, rather than untargeted sequencing. Chen et al. [18] provides the corpus’s oral-to-gut causal evidence, combining observational profiling of saliva and subgingival plaque in a Chinese cohort with an experimental salivary-gavage transmission model in mice. Guo et al. [32] employed a four-group design (healthy controls, periodontitis only, hypertension only, and comorbid) to isolate hypertension-specific from periodontitis-related oral microbial signals; the hypertension-only versus healthy-control contrast is included in the primary oral-compartment synthesis. Finally, Al-Maweri et al. [23], a systematic review pooling 17 studies and 6007 subjects, is retained as a narrative benchmark rather than a primary data source, consistent with this review’s exclusion of secondary syntheses from the primary corpus.

Compared with the gut compartment’s twenty-six primary reports, the oral compartment’s evidence base is markedly smaller and methodologically more heterogeneous: one prospective cohort, one targeted-qPCR case–control study not directly comparable to sequencing-based work, and one causal/experimental study, with no cross-sectional 16S case–control study of normotensive-vs-hypertensive adults independent of these three.

#### 3.1.2. Diversity Measures in the Oral Compartment

No alpha-diversity effect size is extractable for the oral compartment. LaMonte et al. [16] reported observed OTUs, Chao1, and Shannon index as not significantly different across blood-pressure categories (*p* > 0.05 for all three indices), but this null result is presented only as *p*-values on box plots (that study’s own Supplementary Figure S1), without means, standard deviations, or an effect size with confidence interval—precluding its inclusion in a descriptive plot alongside the gut-compartment estimates, though it is reported narratively here as a null finding from the compartment’s only prospective study. Barbadoro et al. [17] used a targeted qPCR panel rather than untargeted 16S sequencing, so no community-level alpha-diversity index was computed at all—a methodological feature of the study design, not a reporting gap. The diversity evidence for the oral compartment therefore stands in explicit contrast to the gut compartment, where five studies contributed descriptive effect sizes: here, the evidence base does not currently support even a narrative comparison of diversity estimates across studies.

#### 3.1.3. Taxon-Level Findings in the Oral Compartment

LaMonte et al. [16] tested 245 oral OTUs against incident hypertension using Cox proportional-hazards models, reporting hazard ratios with 95% CI for every OTU (the original paper’s Table 3 and its own Supplementary Table S4)—the corpus’s first systematically extractable per-species effect-size dataset for the oral compartment. Fifteen species were significant before correction for multiple comparisons (ten with HR 1.10–1.16 indicating increased risk, five with HR 0.82–0.91 indicating decreased risk); after Benjamini–Hochberg correction, none remained significant. This null result after correction stands in explicit contrast to HELIUS’s gut-compartment taxon-level findings, where 62 of 225 tested ASVs remained significant after equivalent correction, a compartment-level asymmetry worth noting when weighing the relative strength of oral versus gut taxon-specific evidence in this review’s overall synthesis.

Barbadoro et al. [17] reported several individually significant species-level differences from targeted qPCR quantification: *Streptococcus mutans* was more abundant in normotensive participants; *Treponema denticola* and *Aggregatibacter actinomycetemcomitans* were more abundant in hypertensive participants (supragingival plaque and, for *A. actinomycetemcomitans*, subgingival plaque). Independently of taxonomic data, salivary nitric oxide (NO) was substantially higher in normotensive participants (165.77 ± 61.7 vs. 57.49 ± 19.61 μmol/L; *p* = 0.023) and, in multivariate logistic regression adjusting for BMI, inversely associated with hypertension (OR 0.33, 95% CI 0.13–0.86; *p* < 0.05), alongside an independent positive association between elevated BMI and hypertension (OR 1.55, 95% CI 1.07–1.63). This biochemical effect size is reported here as complementary mechanistic evidence for the entero-salivary nitrate–nitrite–NO pathway (Section 1), rather than as a taxonomic finding in the strict sense.

Because Barbadoro et al. [17] used targeted qPCR rather than untargeted sequencing, its species-level findings are not directly poolable in a forest plot alongside LaMonte’s 16S-derived hazard ratios; the two studies’ taxonomic evidence is synthesised narratively, by convergence in direction and mechanism (Section 3.1.4), rather than combined quantitatively.

#### 3.1.4. Convergent Taxon: *Neisseria subflava*

*Neisseria subflava* is the oral compartment’s single convergent taxon-level finding, and does so across two studies that differ in design, population, and quantification method—a form of convergence this review considers particularly robust precisely because it is not attributable to shared methodology.

In LaMonte et al. (2022) [16], *N. subflava* was the only nitrate-reducing species among the fifteen OTUs significantly associated with incident hypertension before correction, with an inverse hazard ratio (HR 0.89 in the fully adjusted model; the age-adjusted estimate is 0.90, 95% CI 0.81–1.00, which touches the null) indicating lower risk with greater abundance—directionally consistent with a protective, nitrate-reducing role, though this finding did not survive correction for multiple comparisons.

In Barbadoro et al. [17], *N. subflava* was significantly more abundant in normotensive than hypertensive participants by targeted qPCR (9090.88 ± 5481.49 vs. 4791.35 ± 4349.37; *p* < 0.001), independently reinforcing the same protective direction via an entirely different quantification method (targeted qPCR vs. 16S sequencing) in an entirely different population (Italian case–control vs. US prospective cohort of postmenopausal women).

Both studies converge on the same proposed mechanism—nitrate reduction to nitrite and subsequently nitric oxide by this commensal species—and Barbadoro’s independent biochemical confirmation of higher salivary NO in normotensive participants provides a direct mechanistic link rather than a purely statistical association. Given the compartment’s otherwise sparse taxon-level evidence, this cross-study, cross-method convergence on *N. subflava* is, in this review’s assessment, the oral compartment’s key taxonomic finding, worth weighing alongside *Ruminococcus gnavus*’s cross-population convergence in the gut compartment when the two compartments are compared in Section 4.

#### 3.1.5. Causal Evidence: Oral-to-Gut Microbial Transmission

Chen et al. [18] provided this review’s evidence for a causal, mechanistic link between the oral and gut compartments. In the observational component, 14 salivary genera and 15 subgingival genera were significantly altered in hypertensive participants relative to normotensive controls. In the experimental component, gavage of saliva from human donors into mice demonstrated that oral bacteria can translocate to, and colonise, the gut, directly linking the two compartments rather than treating them as independent. Sixteen species across five genera were identified as recurrent oral–gut transmitters, established at strain level by single-nucleotide-variant distance between paired oral and gut metagenomes rather than by taxonomic co-occurrence, four of them classed as frequent transmitters (*Streptococcus equinus*, *Streptococcus parasanguinis*, *Veillonella parvula* and *Veillonella atypica*), with *Veillonella* identified as the most frequent transmitter, stably enriched in hypertensive participants in both the oral and gut compartments.

This finding is synthesised, per Section 2, alongside the gut compartment’s own causal evidence—Li et al.’s (2017) [4] germ-free-mouse faecal transplantation and Li et al.’s [5] Mendelian randomisation—as one of three methodologically independent lines of causal evidence in this corpus, and is, together with Li et al. [5], one of only two studies in the entire review employing an experimental transplantation design in animals to establish directionality rather than relying solely on human observational data.

#### 3.1.6. Sex Axis: The Oral Compartment

LaMonte et al. [16], the oral compartment’s only prospective study, enrolled exclusively postmenopausal women, making it—alongside the gut compartment’s TwinsUK/PREDICT-1 discovery-replication study [27]—one of two single-sex studies in this review’s corpus. Unlike the TwinsUK/PREDICT-1 study, LaMonte’s single-sex design was not accompanied by a sex-stratified comparison within the study itself (there being no male comparison group), so it cannot contribute a within-study sex-difference estimate to this review’s sex axis in the way HELIUS’s stratified analyses do. Its relevance to the sex axis is instead indirect: a postmenopausal population is one in which oestrogen-related changes to the entero-salivary nitrate pathway have been proposed in the wider literature as a potential modifier of nitrate-reducing bacteria’s cardiovascular relevance, a hypothesis this review’s corpus can flag but not directly test given the absence of a premenopausal or male comparison arm within any oral-compartment study.

#### 3.1.7. Separating Hypertension from Periodontitis: A Four-Group Design

Guo et al. [32] employed a four-group design (healthy controls, periodontitis alone, hypertension alone, and comorbidity) that isolates hypertension-specific from periodontitis-related oral microbial signals; the hypertension-vs-controls comparison is therefore included in the primary oral synthesis.

#### 3.1.8. Benchmark Comparison: Existing Oral-Only Systematic Review

Al-Maweri et al. [23] conducted a systematic review—without quantitative pooling—of 17 studies (6007 subjects) on the oral microbiome and hypertension, reporting qualitative convergence on *Aggregatibacter*, *Kingella*, *Lautropia*, and *Leptotrichia*, together with red-complex periodontal pathogens and, after FDR correction, on *Atopobium*, *Prevotella*, and *Veillonella*. None of these taxa overlap with this review’s own convergent finding of *N. subflava*, though the *Veillonella* signal is notable alongside this review’s own identification of *Veillonella* as the corpus’s principal oral–gut transmitter [18], a point of qualitative convergence between the two reviews that is taken up in Section 4, where the dual character of this genus is examined. As with Avram et al.’s gut-only meta-analysis [22], direct point-by-point comparison between Al-Maweri et al.’s qualitative synthesis [23] and the present review’s oral-compartment findings is reserved for Section 4, where this review’s multi-compartment scope and its explicit sex-axis and causal-evidence sub-analyses are addressed as points of differentiation.

#### 3.1.9. Summary: Taxonomic and Biochemical Convergence in the Oral Compartment

Table 1 summarises the oral-compartment convergence findings of this review.

### 3.2. The Gut Bacteriome

#### 3.2.1. Overview of Included Studies

The gut bacteriome is the most extensively represented compartment in this review, comprising the largest number of primary studies of any compartment in this review (Table 2), spanning cross-sectional case–control designs, large population-based cohorts, long-term prospective cohorts, a Mendelian randomisation study, and the corpus’s landmark causal study. Populations represented include Finland (FINRISK, two independent analyses) [33,34], the Netherlands (HELIUS, multi-ethnic) [25,35], the United Kingdom (TwinsUK/PREDICT-1, female-only) [27], the United States (CARDIA) [36], China (multiple independent cohorts, including four case–control studies contributing to the convergence counts below, and separate cohorts from the north-west and from Assam-adjacent trading regions) [4,37,38,39,40,41,42], Mexico (first Latin American population in the corpus) [43], Brazil [39], South Korea [44], India (two agropastoralist communities in Assam, the only South Asian population in the corpus) [45], Belgium (male-only, with 24 h ambulatory monitoring) [46], and Colombia [47]. One further study (Avram et al., 2025) [22] is a competing meta-analysis retained as a narrative benchmark rather than pooled, consistent with the exclusion criteria specified in Section 2.

Three studies were excluded from the primary normotensive-vs-hypertensive synthesis owing to within-category comparison designs and are addressed in a dedicated sub-analysis (Section 3.2.8): Liu et al. (2026) [48], comparing hypertensive patients across two geographically distinct Chinese cities; Valdez-Palomares et al. (2025) [43], comparing controlled vs. uncontrolled hypertension under treatment; and, in the oral compartment, Guo et al. [32].

#### 3.2.2. Diversity Measures in the Gut Compartment

Alpha diversity effect sizes were extractable, with a defined metric and outcome, from five gut-compartment studies. Consistent with this review’s systematic review (not meta-analysis) scope, no statistical pooling was undertaken for any subgroup; all five estimates are reported descriptively, side by side.

**Prevalent hypertension, odds ratio metric (k = 2).** Sun et al. [36] reported an inverse association between gut microbial richness and prevalent hypertension in a fully adjusted model (OR 0.75 (95% CI 0.60–0.94; office blood pressure measurement), per SD increase; N = 529); the Shannon index was not significantly associated with hypertension in the same model, though it was associated with lower continuous systolic blood pressure (−1.44 mmHg per SD increase, 95% CI −2.72 to −0.16). Palmu et al. [33] reported a comparable inverse association between Shannon diversity and prevalent hypertension in an age/sex-adjusted model (OR 0.91, 95% CI 0.86–0.96; N = 6953), which did not remain significant in the fully multivariable-adjusted model. The two studies are qualitatively concordant in direction, but with only two studies available for this specific metric–outcome combination and, given this review’s systematic review (not meta-analysis) scope, no statistical pooling of the two estimates was undertaken.

**Incident hypertension, hazard ratio metric (k = 1).** The FINRISK 20-year cohort study [34] found no association between Shannon diversity and incident hypertension over 20 years of follow-up in either an age/sex-adjusted model (HR 0.93, 95% CI 0.87–1.00, *p* = 0.05) or the fully multivariable-adjusted model (HR 0.99, 95% CI 0.92–1.07, *p* = 0.84; N = 3311)—a precise, quantitatively reported null finding from the corpus’s longest-duration prospective study.

**Prevalent hypertension, β-coefficient metric, female-only population (k = 1).** Louca et al. [27] reported significantly lower observed ASVs (a proxy for alpha diversity) in hypertensive women in the TwinsUK discovery cohort (β = −0.05, 95% CI −0.095 to −0.004, *p* = 0.03), a finding not independently replicated for this specific alpha-diversity metric in the PREDICT-1 replication cohort within the same publication (replication in this study focused on taxon-specific findings; Section 3.2.4).

**MACE outcome, hazard ratio metric—not a hypertension outcome (k = 1, contextual only).** The HELIUS cohort [35] found Shannon diversity inversely associated with major adverse cardiovascular events (MACEs) in an age-adjusted model (HR 0.83, 95% CI 0.71–0.98) and with the broader MACE+ outcome (HR 0.80, 95% CI 0.71–0.90), though this association did not survive full adjustment for sex, BMI, smoking, and alcohol. This finding is reported here for context given the same cohort’s substantial contribution to gut-compartment taxon-level findings (Section 3.2.4, Section 3.2.5 and Section 3.2.6), but as a cardiovascular surrogate outcome rather than hypertension per se, it is not combined with the hypertension-outcome estimates above. For hypertension incidence specifically, the HELIUS cohort reported alpha diversity as significantly lower at baseline among participants who went on to develop hypertension, but only as a *p*-value from Mann–Whitney testing, without an extractable hazard ratio for this specific outcome.

A sixth study, Cheng et al. [40], a case–control study of 205 elderly Chinese participants (153 hypertensive, 52 controls; Lishui, Zhejiang), found no significant difference in any alpha diversity index (Shannon, Simpson, ACE, Chao1, observed species) between hypertensive and control groups, despite significant beta-diversity separation between groups (ADONIS *p* < 0.01)—a pattern of null alpha diversity alongside significant beta diversity also observed in the corpus’s oral-compartment prospective study (LaMonte et al., 2022 [16]; Section 3.1.2), suggesting that this dissociation may not be compartment-specific. This study did not report an extractable effect size (OR/HR/β) for alpha diversity, precluding its inclusion in the descriptive plot alongside the five estimates above, but its null result is reported narratively as a sixth, concordant data point. A seventh study, Qu et al. [50]—a low-quality case–control study of 63 hypertensive patients and 34 normotensive spouse-controls (Section 3.2.8)—reported significantly lower Chao1, ACE and observed-species diversity in hypertensive patients without cognitive impairment than in the spouse group; the direction is concordant with the majority pattern below, but the complete absence of confounder adjustment and correction for multiple comparisons (NOS 3/9, Poor) means this comparison carries essentially no evidential weight beyond narrative mention.

A further study, Song et al. [44], reported that the association between gut microbiota and hypertension in a Korean population was conditional on enterotype, being detectable in some community configurations and not in others. This is not an alpha-diversity estimate in the sense used above, and it is not pooled with the estimates here, but it bears directly on why single-taxon comparisons across studies replicate poorly (Section 4). Considered together, the gut-compartment alpha-diversity findings are directionally mixed but lean toward null-to-modest associations: four of five extractable estimates point toward lower diversity being associated with higher hypertension/cardiovascular risk, one fully adjusted long-follow-up study finds a precise null, a sixth study finds no alpha-diversity difference at all despite a clear compositional (beta-diversity) separation, and a seventh, very-low-quality study reports a directionally concordant but uninterpretable difference. The estimates span three different outcome definitions and three different effect metrics, precluding any single quantitative summary under the pre-specified pooling rules (Section 2).

#### 3.2.3. Taxon-Level Findings in the Gut Compartment

Two studies contributed exceptionally rich taxon-specific datasets to the gut compartment, transforming the taxon-level evidence in this compartment from a largely qualitative vote-counting exercise into one supported by extensive individually reported effect sizes.

HELIUS [35] tested 225 amplicon sequence variants (ASVs) against incident hypertension in 2097 participants without baseline hypertension, of which 62 remained significant after full Benjamini–Hochberg correction (adjusted for age, sex, BMI, smoking, alcohol)—the largest fully-corrected taxon-specific hypertension dataset in the corpus. Four ASVs were associated with increased risk: *Streptococcus* spp. (OR 1.13, 95% CI 1.05–1.22), *Flavonifractor plautii* (OR 1.17, 95% CI 1.08–1.27), *Ruminococcus gnavus* group (OR 1.07, 95% CI 1.01–1.14), and, counterintuitively relative to most prior literature, *Bifidobacterium* spp. (OR 1.06, 95% CI 1.01–1.12). Fifty-eight ASVs were associated with decreased risk, predominantly from the *Lachnospiraceae* family (20 ASVs, including *Blautia*, *Marvinbryantia*, *Eubacterium*), together with 13 *Oscillospiraceae*, 6 *Ruminococcaceae*, and 5 *Christensenellaceae* ASVs.

Louca et al. [27] identified ten genera differentially abundant in hypertensive TwinsUK women (discovery, FDR < 0.05), of which two were independently replicated in the PREDICT-1 cohort with inverse-variance random-effects meta-analysis already conducted by the study authors themselves: *Ruminiclostridium 6* was less abundant in hypertensive cases (meta-analysis β = −0.31, 95% CI −0.5 to −0.13, *p* = 0.001), and *Erysipelotrichaceae UCG-003* was more abundant (meta-analysis β = 0.46, 95% CI 0.3–0.62, *p* = 0.0001). Because this discovery-replication design was conducted and meta-analysed internally by the original study authors, it is treated in the present review as a single, already-validated evidence unit for these two genera, rather than contributing two separate “votes” to the cross-study convergence.

#### 3.2.4. Convergent Taxon: *Ruminococcus gnavus*

The *Ruminococcus gnavus* group is the single most consistently replicated taxon in the gut compartment of this review, and—combining across compartments—the most robustly convergent finding in the corpus as a whole (cross-compartment synthesis to follow in Section 4). Within the gut compartment alone, an elevated abundance of *R. gnavus* group was independently associated with hypertension-related outcomes in three populations spanning two continents:

Two independent Chinese cross-sectional cohorts (identified in the corpus prior to the present search update, both reporting increased *R. gnavus*/*Mediterraneibacter gnavus* abundance in hypertensive participants).

HELIUS [35], a large Dutch multi-ethnic prospective cohort, in which *R. gnavus* group abundance was independently associated with incident hypertension (OR 1.07, 95% CI 1.01–1.14), incident dyslipidaemia (OR 1.12, 95% CI 1.06–1.19), and, in age-adjusted analysis, MACE+ (HR 1.10)—the only ASV in the HELIUS MACE+ analysis associated with increased rather than decreased cardiovascular risk among the 23 taxa examined.

The HELIUS study additionally provided exploratory serum metabolomic correlations (N = 105 sub-cohort) for the *R. gnavus* group specifically, finding its strongest positive correlations with isoursodeoxycholate (a secondary bile acid; ρ = 0.41) and two acylcarnitine species (ρ = 0.39–0.41), and negative correlations with a xenobiotic metabolite and an androgenic steroid conjugate; offering a tentative mechanistic link between this taxon’s association with hypertension/MACE risk and bile acid and fatty-acid oxidation metabolism, an association the original authors note is directionally counterintuitive given that secondary bile acids are generally considered cardiometabolically protective, and which they discuss as warranting further mechanistic investigation rather than treating as resolved.

This three-population convergence, Chinese cross-sectional data replicated in a methodologically distinct, prospective, Western European multi-ethnic cohort, represents, in the present review’s assessment, the strongest taxon-level signal in the entire corpus, gut or oral.

#### 3.2.5. Additional Convergent and Emerging Gut Taxa

Beyond *R. gnavus*, structured vote-counting identified the following patterns:

• ***Faecalibacterium*** (↓, short-chain fatty acid producer): depleted in hypertension in four independent studies—Li et al. [4], Mushtaq et al. [41] in China, and Silveira-Nunes et al. [51] in Brazil. Zuo et al. [42] is noted separately: its taxa were pre-selected from an earlier publication because they distinguished hypertension in that dataset, which constitutes circular selection rather than blind independent replication; the convergence count for Faecalibacterium therefore rests on three independently profiled studies (Li, Mushtaq, Silveira-Nunes). The Brazilian replication matters: it places this signal outside East Asia, which the other convergent gut taxa in this review do not achieve. The pattern was not independently tested as a distinct finding in HELIUS, though the broader *Lachnospiraceae*-family protective signal in that cohort (Section 3.2.3) is consistent in direction.
• ***Roseburia*** (↓, short-chain fatty acid producer): depleted in four studies—Li et al. [4], Yan et al. [39] and Zuo et al. [42] in China, and Silveira-Nunes et al. [51] in Brazil—now joined by a fifth independent study: Cheng et al. [40], in a case–control cohort of 205 elderly Chinese participants, reported *Roseburia* among the beneficial, butyrate-producing genera significantly depleted in hypertensive participants, alongside *Blautia* and *Butyricicoccus*—the latter two representing additional convergent signal with the broader SCFA-producer-depletion pattern described here, though not previously counted as independently convergent taxa in their own right given single-study status prior to this addition. *Roseburia hominis* specifically also appeared among the HELIUS [35] MACE/MACE+-protective ASVs, consistent in direction though for a cardiovascular rather than hypertension-specific outcome. Cheng et al. [40] additionally reported *Escherichia_Shigella*, *Prevotella_9*, and *Enterococcus* enriched in hypertensive participants, and—via random forest and ROC analysis—identified *Blautia*, *Butyricicoccus*, *Lachnoclostridium*, *Prevotella_9*, and *Enterococcus* as a five-genus diagnostic panel (individual AUC 0.61–0.71; combined AUC 0.78), the corpus’s most complete diagnostic-biomarker dataset for the gut compartment to date.
• ***Klebsiella*** (↑): elevated in two independent Chinese studies, Li et al. [4] (16S amplicon) and Yan et al. [39] (whole-metagenome shotgun)—platforms that differ in resolution and amplification bias, so the convergence carries this caveat. This meets the two-study convergence threshold, but with an important qualification absent from earlier accounts of this signal: a United States case–control study [8] reported the genus as depleted rather than enriched; however, the 18 hypertensive participants in that study had a mean blood pressure of 160/100 mmHg while on antihypertensive medication, rendering them uncontrolled treated hypertensives rather than untreated hypertensives as in the Chinese studies. The apparent geographic divergence may therefore reflect a difference in hypertension phenotype rather than in gut microbial ecology. It was not independently confirmed as a distinct signal in the three most recently added studies (HELIUS [35], Louca et al. [27], Liu et al. geographic comparison [48]), though the latter did report elevated *Escherichia*/*Klebsiella* (Enterobacteriaceae) abundance in the tropical-climate relative to temperate-climate (Daqing) hypertensive group—a geographic rather than normotensive-vs-hypertensive comparison but directionally consistent with the broader Enterobacteriaceae-elevation pattern.
• ***Ruminiclostridium 6*** (↓) and ***Erysipelotrichaceae UCG-003*** (↑): newly identified in this review via Louca et al. [27], with internal discovery-replication meta-analysis already conducted by the original authors, *Erysipelotrichaceae*, as a family, has prior associations with pro-inflammatory states (irritable bowel syndrome, rheumatoid arthritis) in the wider literature, lending mechanistic plausibility to this signal, though it has not yet been independently replicated by a second research group.
• ***Streptococcus*** spp. (↑), ***Flavonifractor plautii*** (↑), ***Bifidobacterium*** spp. (↑): newly identified via HELIUS [35] as associated with incident hypertension; *Flavonifractor plautii* was additionally associated with incident diabetes and dyslipidaemia in the same cohort, suggesting a cross-cardiometabolic-outcome signal rather than a hypertension-specific one. The *Bifidobacterium* finding is directionally counterintuitive relative to the wider probiotics literature, in which this genus is generally reported as protective; the original HELIUS authors discuss this as potentially reflecting species-level heterogeneity within the genus or urbanisation-related shifts in *Bifidobacterium* community composition, an interpretation this review reports without independent adjudication.

#### 3.2.6. Multi-Compartment Causal Evidence: The Gut Compartment

Li et al. [4] provided the corpus’s landmark causal evidence: faecal microbiota from hypertensive human donors, transplanted into germ-free mice, induced elevated blood pressure in recipient animals, establishing gut dysbiosis as sufficient—in this causal model—to produce a hypertensive phenotype, independent of host genetic or long-term environmental factors.

Li et al. [5] applied Mendelian randomisation using genetic instruments for gut microbiota composition, providing genetically informed causal evidence complementary to, and methodologically independent of, the experimental transplantation design of Li et al. [4]. Together, these two studies represent the corpus’s strongest causal-inference evidence for a gut-microbiota contribution to hypertension, and are discussed jointly, and separately from purely observational associative findings, in this review’s dedicated causal-evidence synthesis.

#### 3.2.7. Sex and Ethnicity Axes: Gut-Specific Findings

The gut compartment is the primary evidentiary basis for both of this review’s primary analytical axes (Section 1).

**Sex axis.** Louca et al. [27] is, within the entire corpus, the single study of highest methodological quality specifically addressing the sex axis: an exclusively female discovery cohort (TwinsUK, N = 871) with independent replication in a second exclusively female cohort (PREDICT-1, N = 448), yielding two genera with author-conducted meta-analysed effect sizes (Section 3.2.3, Section 3.2.4 and Section 3.2.5) directly usable in this review’s sex-stratified narrative synthesis.

**Ethnicity axis.** HELIUS [25,35] is, within the entire corpus, the key study for the ethnicity axis, with formal stratified analysis across Dutch, African Surinamese, and South-Asian Surinamese participants—the three largest ethnic groups in the cohort. Across the 23 gut-microbiota associations examined for MACE/MACE+, no significant ethnicity interactions were detected in the Benjamini–Hochberg-corrected main analysis; the 13 ASVs reported to show ethnicity-by-ASV interactions in sub-analyses were tested at *p* < 0.05 without multiple-testing adjustment and carry a less stringent evidential weight than the corrected results. Associations were predominantly driven by Dutch and African Surinamese participants and were frequently attenuated or absent in South-Asian Surinamese participants—a pattern the original authors attribute at least partly to reduced statistical power in this subgroup, though a genuine biological difference in microbiota–hypertension association across ethnic groups cannot be excluded on the basis of this cohort alone. For new-onset hypertension specifically, 13 of the significant ASV associations showed formal ethnicity interactions (predominantly within the *Lachnospiraceae* and *Oscillosporaceae* families), with protective associations again generally stronger in Dutch and African Surinamese participants than in South-Asian Surinamese participants. Sex-stratified analysis within HELIUS additionally found associations with new-onset diabetes, and to a lesser extent hypertension, generally stronger in women than in men—a finding the original authors relate to prior literature on sex-specific gut microbiome–hormone interactions in glucose metabolism, and which is discussed in this review alongside the TwinsUK/PREDICT-1 sex-axis findings above as convergent, cross-cohort evidence for sex-dependent heterogeneity in gut microbiota–cardiometabolic associations, notwithstanding the different specific taxa and outcomes involved.

#### 3.2.8. Studies Excluded from Primary Pooling: Within-Category Gut Comparisons

Three gut-compartment studies employed comparisons not directly equivalent to the primary normotensive-vs-hypertensive contrast and are synthesised here as dedicated sub-analyses rather than pooled with the sections above. The third, Qu et al. [50]—a case–control study, reclassified from the source’s own “cohort” label on the basis of its Methods, which describe single-hospital recruitment without follow-up—recruited 63 hypertensive patients and, as a normotensive comparator intended to control for shared diet and household environment, 34 of their spouses. Its principal analysis compares hypertensive patients with and without cognitive impairment, identifying gut microbiome signatures predictive of cognitive status—informative for the microbiota–end-organ-damage question rather than for hypertension itself. The study does, however, report a hypertension-relevant finding: alpha diversity (Chao1, ACE, observed species) was significantly lower in hypertensive patients without cognitive impairment than in the normotensive spouse group. This comparison is included, with an explicit quality caveat, in the gut alpha-diversity synthesis above (Section 3.2.2), given the study’s very low methodological quality (3/9, poor: no adjustment for confounders, no correction for multiple comparisons).

Liu et al. [48] compared hypertensive patients from two climatically distinct Chinese cities, finding a lower Firmicutes/Bacteroidetes ratio in the temperate-climate group and six genera independently associated with geographic grouping after adjustment for age, BMI, smoking, and drinking status. This geographic comparison, between two equally hypertensive populations rather than hypertensive-vs-normotensive, is discussed as hypothesis-generating evidence for environmental/geographic modulation of the hypertension-associated gut microbiome, a dimension largely unaddressed elsewhere in the corpus, but is explicitly not combined with the primary gut bacteriome synthesis above given its fundamentally different comparator group.

Valdez-Palomares et al. [43] compared gut microbiota composition between older Mexican adults with controlled vs. uncontrolled hypertension, all under antihypertensive treatment—again a within-hypertensive comparison rather than a normotensive-vs-hypertensive one. *Escherichia-Shigella* abundance increased with age; alpha diversity decreased with age; and *Ruminococcus UCG-002*, *DTU089*, and members of the *Lachnospiraceae* family (short-chain fatty acid producers) were distinctively abundant in the controlled-hypertension group. As the first Latin American population in this review’s corpus, this study is discussed as an important contribution to the ethnicity/geography axis (Section 3.2.7) despite its within-category design precluding inclusion in the primary compartment synthesis.

A further gut-compartment study, de la Cuesta-Zuluaga et al. [47], also does not contribute a primary normotensive-vs-hypertensive estimate, for a different reason: blood pressure is used only as a descriptive covariate characterising microbial co-abundance groups (CAGs) in a general Colombian population cohort (N = 441), not as an outcome analysed in a dedicated multivariable model. No adjustment for cardiometabolic confounders is applied to a microbiota–blood pressure relationship, because no such relationship is directly modelled; the study’s own analyses are Spearman correlations, ANOVA across CAG subgroups, and random forest classification of microbial configurations. Its short-chain fatty acid data, sometimes cited elsewhere for cross-study comparison, are inferred functionally (Tax4Fun on SILVA 123) rather than measured directly (Section 3.2.3).

A third study, Liu et al. [49], analysed the Guangdong Gut Microbiome Project (N = 6999) using a co-abundance network approach rather than the individual-taxon differential-abundance framework used elsewhere in this review, identifying 581 hypertension-related microbial co-abundances (68.7% replicated across two independent external cohorts); given the methodological incompatibility with the review’s vote-counting synthesis strategy, this study is discussed narratively as complementary network-level evidence rather than pooled.

#### 3.2.9. Benchmark Comparison: Existing Gut-Only Meta-Analysis

Avram et al. [22] conducted a formal meta-analysis restricted to the gut compartment, without integration of oral or fungal data and, as discussed in Section 2, without an explicit pre-specified minimum-study threshold for quantitative pooling. That review, and the earlier observational-studies synthesis of Guo et al. [24], are the two gut-compartment benchmarks against which the present findings are read. Direct point-by-point comparison of specific pooled estimates between that meta-analysis and the present review’s descriptive gut-compartment synthesis is reserved for Section 4, where methodological differences in pooling approach are addressed as a central point of differentiation for this review.

#### 3.2.10. Summary: Taxonomic Convergence in the Gut Compartment

Table 3 summarises the gut-compartment convergence findings of this review.

**Table 3:** Summary of taxonomic convergence in the gut compartment.

Taxon/Finding	Direction	Independent Studies	Populations	Convergence Status
Ruminococcus gnavus* group	↑ (harmful)	3	China ×2, Netherlands (HELIUS)	Convergent—strongest signal in the entire corpus, cross-continental
*Faecalibacterium* *	↓ (protective, SCFA producer)	4	China ×3, Brazil	Convergent; the Brazilian cohort provides the only replication outside East Asia
*Roseburia* *	↓ (protective, SCFA producer)	5	China ×4, Brazil	Convergent; *R. hominis* additionally protective for MACE/MACE+ in HELIUS; Cheng et al. 2025 [40] adds 5th population
*Klebsiella* *	↑ (harmful)	2	China ×2	Meets the threshold but contested: direction reversed in the one United States study to examine it [8]
*Ruminiclostridium 6*	↓ (protective)	1 (with internal discovery + replication)	UK (TwinsUK + PREDICT-1)	Author-validated, single independent research group
*Erysipelotrichaceae UCG-003*	↑ (harmful)	1 (with internal discovery + replication)	UK (TwinsUK + PREDICT-1)	Author-validated, single independent research group
*Streptococcus* spp.	↑ (harmful)	1 (gut)	Netherlands (HELIUS)	Single-study gut signal; cross-compartment echo in oral LaMonte cohort (Section 3.1.3)
*Flavonifractor plautii*	↑ (harmful, multi-outcome)	1	Netherlands (HELIUS)	Single study; also associated with incident diabetes/dyslipidaemia in same cohort
*Bifidobacterium* spp.	↑ (harmful—counterintuitive)	1	Netherlands (HELIUS)	Single study; direction contrary to most prior literature

* Only *Ruminococcus gnavus*, *Faecalibacterium, Roseburia*, and *Klebsiella* meet the pre-specified two-independent-study convergence threshold (Section 2) within the gut compartment.

### 3.3. The Mycobiome

#### 3.3.1. Overview of Included Studies

The fungal component of the microbiota is represented in this corpus by two primary studies. Chen et al. [20] sampled saliva, subgingival plaque, and faeces from the same participants in a Shanghai case–control study using shotgun metagenomic sequencing and is to our knowledge the first multi-compartment characterisation of the fungal microbiome in relation to blood pressure. Zou et al. [19] compared gut mycobiome composition across three groups—normotensive, pre-hypertensive, and hypertensive—in an independent Chinese population, linking fungal composition to serum immunoglobulin light chains. Gao et al. [21], a multi-cohort cross-sectional analysis examining bacterial and fungal communities in parallel across several hypertension cohorts, is retained as a narrative benchmark rather than pooled with the primary corpus; notably, it is the only multi-cohort test of mycobiome reproducibility identified in this literature, and its findings are negative.

Two features of this evidence base constrain what can be concluded from it. First, Chen et al. [20] shares its cohort with the oral-compartment study of the same group [18], and is therefore treated here as an additional compartment of one population rather than as an independent study, in accordance with the eligibility criteria (Section 2). Second, both primary studies were conducted in China, so the entire mycobiome evidence base in this review derives from two East Asian populations, one of which is not independent of the oral bacteriome corpus.

#### 3.3.2. Diversity Measures in the Fungal Compartment

Alpha diversity of the fungal community was substantially higher in subgingival plaque than in saliva or faeces in both hypertensive and normotensive participants, indicating that fungal ecological niches differ across body sites regardless of blood pressure status [20]. Between hypertensive and control groups, alpha diversity did not differ significantly in saliva or subgingival plaque, with a non-significant trend toward higher faecal diversity in hypertension (*p* = 0.09). Beta diversity behaved differently: hypertensive and normotensive participants separated significantly in saliva and in subgingival plaque, but not in faeces [20]. The oral fungal community therefore discriminates by blood pressure status where the gut fungal community does not—the reverse of the pattern seen in the bacteriome, where the gut compartment carries the stronger signal.

This is the third instance in the present corpus of a dissociation between a null alpha diversity and a significant beta diversity, following the same pattern in the oral prospective cohort and in a gut case–control study. Its recurrence in a different kingdom, sampled by a different sequencing approach, argues that the dissociation reflects something about how microbial communities change in hypertension rather than an artefact of any one method.

#### 3.3.3. Taxon-Level Fungal Findings

Among fungi shared between the oral cavity and the gut, the genus Exophiala showed the most pronounced alterations across compartments, with *Exophiala spinifera* the most abundant salivary species in hypertensive participants [20]. Eleven fungal species correlated directly with systolic or diastolic blood pressure: four positively in saliva (*Wickerhamomyces ciferrii*, *Ascoidea rubescens*, *Penicillium rubens*, and *Lodderomyces elongisporus*), one positively in subgingival plaque (*Aspergillus candidus*), and two positively in faeces (*Saccharomyces cerevisiae* and *Exophiala mesophila*), with three negative correlations in plaque and one in faeces (*Exophiala xenobiotica*). No fungal taxon was reported in a concordant direction by two independent, non-overlapping cohorts, so no fungal taxon meets this review’s convergence threshold (Section 2).

Zou et al. [19] add a distinct observation rather than a replication: fungal dysbiosis was already detectable at the pre-hypertensive stage, before a diagnosis of hypertension had been made, and fungal composition was predictive of dysregulated serum immunoglobulin light chains in hypertensive patients. Because the two studies report largely different taxa, their contribution is complementary in framing but not mutually confirmatory.

Interpretation of species-level fungal calls requires unusual caution. Fungal DNA in stool derives substantially from dietary and transient sources rather than from resident colonisers: in healthy adults under controlled diets, most detected fungal sequences track recent food intake and few taxa persist across repeated sampling [52], and the gut mycobiome of healthy individuals is markedly more variable between and within people than the bacteriome [53]. *Saccharomyces cerevisiae*, one of the taxa correlating with blood pressure in faeces above, is also the most common dietary yeast. Neither it nor the other culinary yeasts establish stable residence in the adult gut: they appear in stool as passengers of recent meals rather than as members of a colonising community. The distinction is analytical as well as biological, because amplicon sequencing of the ITS region recovers fungal DNA irrespective of viability, so the dead yeast of a meal and a living coloniser are indistinguishable in the resulting profile. Some of the fungal signal reported in this literature may therefore reflect diet rather than a colonising community, and no included study adjusted for dietary fungal intake.

Two consequences follow for how this finding should be read and what would be required to test it properly. It should be read, at present, as a correlation between a dietary exposure and blood pressure that has been measured through stool rather than through a food questionnaire; the possibility that it reflects a host–microbe relationship remains open but is not the more parsimonious reading. Both primary mycobiome studies in this corpus excluded participants who had taken probiotics in the preceding two months [19,20], which removes the most concentrated source of exogenous yeast but not the ordinary dietary one—bread, beer, wine and other fermented foods—and neither study recorded it. Testing the finding would require three things that no available study provides. First, dietary fungal intake should be recorded and entered as a covariate, which is feasible with a food-frequency instrument extended to leavened and fermented items and to yeast-based supplements, including Saccharomyces boulardii, itself a strain of *S. cerevisiae*. Second, repeated sampling is needed to separate transient from resident taxa, since a single stool sample cannot distinguish them by definition. Third, viability-resolved methods—RNA-based profiling, culture, or DNA treatment with propidium monoazide before amplification—would establish whether the organisms detected are alive in the gut at all. Until a study meets at least the first of these conditions, species-level fungal associations with blood pressure should be reported as hypothesis-generating and should not enter convergence counting, which is how they are treated here.

#### 3.3.4. Collapse of Fungal Ecological Networks

The most striking observation in this compartment is not about which fungi are present but about how they relate to one another. Fungal co-correlation networks were drastically impoverished in hypertensive participants across all three sampled sites: from twenty-two significant correlations to three in saliva, twenty-three to three in subgingival plaque, and twenty-six to one in faeces [20]. Notably, this collapse occurred in faeces even though faecal beta diversity did not differ significantly between groups—the community composition was statistically indistinguishable while its internal organisation was not.

That dissociation is the clearest single illustration in this corpus of the limitation of abundance-based analysis, and it is developed further in the Discussion. Fungi are plausible participants in such structure because they interact with bacteria and with host immunity rather than existing as passive passengers; commensal fungal recognition through C-type lectin receptors shapes intestinal immune tone in experimental models [54], which offers a mechanism by which a disorganised fungal community could matter without any single species being differentially abundant.

#### 3.3.5. Contradictory Cross-Cohort Evidence

Against the two positive reports stands a cross-cohort microbiome-wide study that examined bacterial and fungal communities in parallel and found consistent alterations in the gut bacteriome but no consistent alteration in the gut mycobiome [21]. This study was designed across multiple cohorts specifically to test reproducibility, and its null finding for the fungal compartment carries the weight that a well-powered, purpose-designed replication attempt deserves. It does not contradict the network observation above, which it did not test, but it does contradict the proposition that fungal abundance profiles distinguish hypertensive from normotensive individuals in a reproducible way.

The mycobiome is therefore included in this review as an open question rather than as a source of findings. It is the least studied compartment, the most methodologically fragile, and the only one in which the largest available study is negative.

#### 3.3.6. Summary: The Fungal Compartment

Table 4 summarises the mycobiome findings and their status in this review.

No mycobiome finding in this review meets the two-independent-study convergence threshold, and the only multi-cohort test of reproducibility is negative. The fungal compartment contributes one structural observation of potential importance and no established taxonomic finding.

### 3.4. Certainty of Evidence Across Compartments

Certainty was assessed per key convergent finding using GRADE adapted for exposure–outcome questions. Because the evidence base is entirely observational, each body of evidence started at low certainty. Table 5 reports the resulting judgements with the domains driving each.

Every taxon-level finding in this review is very-low-certainty evidence, including the most replicated one; the only finding rated low rather than very low is a biochemical measurement rather than a microbial abundance. It should be read for what it is. Each rating concerns one specific claim of the form “this organism is more or less abundant in hypertension”, and the ratings are not a verdict on the broader question of whether the microbiota is associated with blood pressure, which was not assessed as a single GRADE outcome and which rests on a different and considerably stronger evidence base—community-level compositional differences reported in every compartment examined, and experimental transfer models. The implications of this distinction are addressed in Section 4.

## 4. Discussion

This review set out to ask whether the microbiota’s relationship with blood pressure looks the same when the gut, the oral cavity, and the fungal community are examined side by side rather than one at a time. The answer is that it does not, and that the differences between compartments are more informative than the individual taxon lists each compartment generates. The gut bacteriome contributes the largest body of evidence and the only findings replicated across continents; the oral bacteriome contributes the most coherent mechanism, in the entero-salivary nitrate–nitrite–nitric oxide circuit, but rests on a much thinner evidential base; the mycobiome contributes a structural observation of possible importance and almost nothing that has been independently confirmed. Cutting across all three is a finding that no single-compartment review could have surfaced: the most consistent signal is not the presence or absence of particular organisms but the loss of organisation within the community, and this appears in more than one compartment and more than one kingdom.

Two levels of claim must be kept apart here, and conflating them would misrepresent this review in either direction. That the microbial community differs between hypertensive and normotensive individuals is well supported: beta diversity separates the groups in essentially every study that measures it, across all three compartments; the largest prospective cohort in the corpus identifies 62 taxa associated with incident hypertension after full adjustment and correction for multiple comparisons; faecal transfer from hypertensive donors raises blood pressure in germ-free recipients; and salivary nitric oxide, the one directly measured biochemical intermediate, is markedly lower in hypertension. The proposition under scrutiny in this review is not whether the microbiota is associated with blood pressure, but whether particular organisms can be identified as carrying that association. It is the second proposition that the evidence does not currently support. No taxon reached moderate certainty on GRADE assessment; no alpha-diversity measure was consistently associated with hypertension once studies adjusted fully for confounders; and the two syntheses that preceded this one, one of the gut compartment and one of the oral compartment, converge on almost none of the same organisms as each other. A reader arriving at this literature from the outside would reasonably expect, given a decade of work and several thousand publications, a shorter and firmer list than the one this review is able to offer.

The strongest taxon-level signal in the corpus is an increased abundance of the *Ruminococcus gnavus* group, reported in two Chinese cross-sectional cohorts and independently confirmed in a large Dutch multi-ethnic prospective cohort, where it was associated with incident hypertension after full adjustment and correction for multiple comparisons, and where it was additionally associated with incident dyslipidaemia and with cardiovascular events [35]. Replication across two continents, two designs, and two sequencing pipelines is precisely what this literature has generally lacked, and the organism is mechanistically plausible: *R. gnavus* degrades intestinal mucin and produces a glucorhamnan polysaccharide that induces TNF-α secretion by dendritic cells through TLR4 [55], which fits the barrier-disruption and low-grade-inflammation pathway sketched in Figure 1.

Yet the finding cannot be rated above very low certainty, and the reason is instructive rather than pedantic. The one rigorously adjusted estimate is an odds ratio of 1.07 with a confidence interval whose lower bound sits at 1.01. An association of that magnitude, however well replicated, is compatible with a large amount of residual confounding by diet, medication, or unmeasured metabolic status, and it is not the kind of effect from which a clinical or therapeutic inference can be drawn. The exploratory metabolomic correlations reported for this taxon point toward bile acid and fatty-acid oxidation metabolism, but in a direction the original investigators themselves describe as counterintuitive, as the secondary bile acid most strongly correlated is generally regarded as cardiometabolically protective. This review therefore reports *R. gnavus* as the corpus’s most convergent finding while declining to describe it as an established one—a distinction the field has not always maintained.

Three mechanisms could connect this organism to vascular phenotype, and they are worth separating because they differ in how much of their support comes from this corpus and how much from elsewhere. The first is the glucorhamnan route already noted: certain strains of *R. gnavus* secrete a complex polysaccharide with a rhamnose backbone that induces TNF-α release from dendritic cells in a TLR4-dependent manner [55]. This is the most completely characterised of the three at molecular level—a purified molecule acting on a defined receptor—but it was established in the context of inflammatory bowel disease and in vitro, and two limitations restrict what it can be asked to explain here. Polysaccharide production is strain-dependent rather than a property of the species, and every study in this corpus that reports *R. gnavus* does so from 16S amplicon or shallow metagenomic data, which cannot establish whether the organisms enriched in hypertensive participants carry the biosynthetic locus at all. No included study measured TNF-α, TLR4 signalling, or the polysaccharide itself in relation to blood pressure.

The second is barrier permeability. *R. gnavus* degrades intestinal mucin, and loss of mucus-layer integrity is the proposed entry point for the translocation and endotoxaemia route sketched in Figure 1 and documented in hypertensive patients [8]. This mechanism has the advantage of connecting to a pathway the present review already reports, but it shares the same evidential gap: no study in this corpus measured intestinal permeability, circulating lipopolysaccharide, lipopolysaccharide-binding protein or zonulin alongside *R. gnavus* abundance, so the link between the organism’s mucin-degrading capacity and any measured barrier state remains inferred rather than observed. It should also be noted that these first two mechanisms are not genuine alternatives. Mucin degradation and TLR4-mediated cytokine release converge on the same low-grade inflammatory endpoint and both act at the epithelium; distinguishing them would require measurements no study has made, and doing so may not be necessary for the purposes of this review.

The third, secondary bile acid dysmetabolism, is the genuinely distinct alternative, and it is the one the available data fit least well. *R. gnavus* carries bile salt hydrolase activity and therefore participates in the deconjugation step upstream of secondary bile acid formation [56], and bile acids signal through FXR and TGR5 with documented vascular consequences, so the pathway is plausible a priori. The only bile acid evidence in this corpus, however, runs against it: the exploratory metabolomic correlations reported for this taxon point toward bile acid and fatty-acid oxidation metabolism in a direction the original investigators themselves describe as counterintuitive, as the secondary bile acid most strongly correlated with the organism is generally regarded as cardiometabolically protective. On present evidence this mechanism is not merely unproven but discordant with the one relevant observation available, and it should be reported as such rather than listed alongside the others as an equally supported candidate. A further constraint applies to all three. Any proposed mechanism must account for an adjusted odds ratio of 1.07, and a strongly inflammatory pathway operating in most carriers would be expected to produce more than that; the magnitude is more consistent with an effect confined to a subset of strains, hosts or contexts than with a uniform mechanism. Discriminating among the three therefore requires measurements that are feasible but absent: strain-resolved metagenomics to establish carriage of the polysaccharide locus, paired inflammatory and permeability markers, and targeted rather than untargeted bile acid quantification. Until those exist, mechanistic attribution for this organism should be regarded as open.

The oral compartment presents the opposite profile. The nitrate–nitrite–nitric oxide pathway is among the best-characterised routes by which any microbial community could plausibly influence blood pressure: dietary nitrate is concentrated in saliva, reduced to nitrite by commensal bacteria that the human host cannot substitute for [14], and converted onward to nitric oxide with direct vascular consequences [15]. The same axis links periodontal disease to blood pressure, an association for which genetic and interventional evidence now exists [57]. Depletion of *Neisseria subflava*, a nitrate reducer, in hypertensive relative to normotensive subjects is reported both in a United States prospective cohort using 16S sequencing of subgingival plaque [16] and in an Italian case–control study using targeted qPCR [17]. Convergence across methods that share no technical assumptions is unusual in this field and, taken at face value, compelling.

It should not be taken entirely at face value. In the prospective study, none of the fifteen species-level associations survived Benjamini–Hochberg correction, *N. subflava* included; the case–control replication comprises forty-eight subjects with acknowledged imbalance in age and oral hygiene between groups. What survives scrutiny best in this compartment is not a bacterium at all but a biochemical measurement: salivary nitric oxide was roughly three-fold lower in hypertensive subjects, with an inverse association robust to multivariable adjustment [17]. This is the only finding in the entire corpus that this review rates at low rather than very low certainty, and it is measured directly rather than inferred from relative abundance. There may be a general lesson here about where the reliably measurable signal in microbiome research currently lies.

The observation this review considers most worth carrying forward is not taxonomic. In the multi-compartment fungal study, co-correlation networks among fungal species collapsed almost completely in hypertensive participants—from twenty-two connections to three in saliva, twenty-three to three in subgingival plaque, and twenty-six to one in faeces [20]. Network-based analyses of the gut bacteriome, which deliberately set aside differential abundance in favour of co-abundance structure, likewise identify organisms that carry information about hypertension without differing in abundance between groups [49]. The same pattern is implied by the recurring dissociation, seen in both the oral prospective cohort and gut case–control studies, between a null alpha diversity and a clearly significant beta diversity: the number of organisms present does not change, but their arrangement does. A Korean study points in the same direction from a different angle, finding that the microbiota–hypertension association held in some enterotypes and not in others [44]—that is, the same taxa carried different information depending on the community configuration they sat in.

If this is real, it has a methodological consequence that reaches beyond hypertension. The dominant analytic paradigm in this literature asks which taxa differ in abundance between cases and controls, and a decade of that question has produced the fragmented and poorly replicated lists this review has had to synthesise. A community can lose its ecological organisation while its census remains unchanged, and differential-abundance testing is blind to exactly that. The instability of that testing is itself documented: applied to the same datasets, fourteen widely used differential-abundance methods identify markedly different numbers and sets of significant features [58], which alone would account for part of the poor replication across this corpus. The proposition is not established—it rests on one multi-compartment fungal study and a small number of network analyses, and no study has yet tested network structure as a prespecified primary outcome against hypertension—but it is the most concrete testable hypothesis this review is able to generate, and it would be straightforward to evaluate in existing cohorts.

Evidence on the fungal component remains genuinely preliminary, and this review is not in a position to resolve it. Two independent studies report fungal dysbiosis associated with hypertension, one of them detecting it already at the pre-hypertensive stage [19], and one describing the network collapse discussed above [20]. Against this sits a cross-cohort study that examined bacterial and fungal communities in parallel and found consistent bacteriome alterations but no consistent mycobiome alteration [21]. That is a direct contradiction, from a study designed with more cohorts and greater statistical power than either of the positive reports, and honesty requires giving it the weight its design deserves. The mycobiome is included in this review because a multi-kingdom framework that omitted it would be incomplete, not because the evidence supports a conclusion. Whether the fungal community matters for blood pressure is, at present, unanswered.

Treating sex and ethnicity as analytical axes rather than adjustment covariates changes what the literature appears to say. Within the single cohort that permits formal stratification, associations between gut microbiota and cardiometabolic outcomes were driven predominantly by Dutch and African Surinamese participants and were frequently attenuated or absent among South-Asian Surinamese participants, with thirteen taxon-level associations showing formal interaction by ethnicity [35]. The original investigators attribute much of this to reduced power in the smaller subgroup, and that may be right, but a genuine biological difference cannot be excluded on the evidence available. Associations were also generally stronger in women than in men in that cohort, converging with the finding that the single methodologically strongest study of the sex axis—a discovery and replication design in two independent female cohorts [27]—identifies genera that appear nowhere else in the corpus. That convergence should be read with the qualification developed below: the female evidence in this corpus comes predominantly from postmenopausal or peri-menopausal samples, so what is supported is a difference between men and largely postmenopausal women rather than between men and women in general.

The sex axis carries a hormonal dimension that the corpus does not resolve and that this review should state plainly, because it bears on the compartment where our evidence is strongest. Oestrogen upregulates endothelial nitric oxide synthase and supports mucosal barrier integrity [59], and both effects decline after the menopause. The first of these intersects directly with the oral finding reported here: nitric oxide is the shared endpoint of two independent routes, host synthesis by endothelial nitric oxide synthase and bacterial reduction of dietary nitrate by the oral commensal community. Menopausal status therefore does not act on this pathway as a generic confounder but as a modifier of the very quantity the pathway produces. The second effect, on barrier function, bears on the translocation and endotoxaemia arm of the same framework.

The composition of the corpus makes this more than a theoretical concern. The oral compartment’s only prospective study is drawn from a cohort of postmenopausal women [16]; the single largest gut study of the sex axis is an all-female twin registry in which cases are on average eight years older than controls, 60.3 against 52.4 years, a difference that spans the menopausal transition, and whose models adjust for age and its square but not for menopausal status [27]; and the one study restricted to men excludes the question entirely by design [46]. No study in the corpus reports menopausal status as a variable, and none stratifies by it. Two consequences follow. The first is interpretive: this review’s observation that associations are generally stronger in women rests on cohorts that are largely or entirely postmenopausal, so the claim that is actually supported is narrower than the one the literature invites, and we have qualified it accordingly. The second concerns the direction of the potential bias, which is not self-evident. Reduced host nitric oxide synthesis after the menopause would raise, not lower, the relative contribution of the bacterial nitrate-reduction route to total nitric oxide availability; a postmenopausal cohort may therefore be a setting in which the oral signal is more readily detected rather than one in which it is spuriously generated. Both readings are consistent with the available data and neither can be preferred on the evidence.

One study allows the concern to be examined rather than only raised. LaMonte et al. [16] recorded current menopausal hormone therapy and reported that its use was lower in women with prevalent hypertension and elevated blood pressure than in normotensive women (*p* = 0.02); hormone therapy was nonetheless not carried into the adjustment set of the multivariable models, which comprise age, race and ethnicity, education, neighbourhood socioeconomic status, self-rated general health, treated diabetes, statin use and diet quality. In the cross-sectional analyses, therefore, an exposure that differs by outcome status is left unadjusted. In the prospective analysis on which the *Neisseria subflava* finding depends, hormone therapy use did not differ significantly by incident hypertension status (*p* = 0.16), which limits but does not eliminate the concern for that estimate. Reporting menopausal status, and hormone therapy where applicable, should be routine in this literature; it is currently absent from every study in this corpus, including those composed entirely of women.

The geographical distribution of the evidence compounds this. The Klebsiella signal rests on two Chinese studies and is contradicted in direction by the one United States study to have examined it; the Faecalibacterium and Roseburia signals rest on three Chinese studies each, with a single Brazilian cohort providing the only replication outside East Asia; a single Mexican study provides the only other Latin American data; a single Swedish cohort and one Belgian male-only cohort provide ambulatory blood pressure data [46,60]; and the only South Asian evidence comes from two agropastoralist communities in Assam [45], with no study conducted in Africa. The Assam cohort is instructive precisely because it is atypical: several genera previously associated with hypertension in industrialised populations were also associated with systolic blood pressure there, which is the kind of cross-lifestyle replication the rest of this corpus cannot supply. It is not currently possible to distinguish a taxon that is associated with hypertension from a taxon that is associated with hypertension in East Asian populations. Given how strongly diet and geography structure the gut microbiome, this is not a minor caveat, and the one study that compared hypertensive populations across two climatically distinct Chinese cities found substantial compositional differences between them [48]—within a single country, and between groups sharing the same diagnosis.

A further source of heterogeneity runs beneath all of the above and is easy to overlook because it concerns the definition of the disease rather than the measurement of the microbiota. The threshold at which a participant becomes a case is not constant across this corpus, and Supplementary Table S3 now records it for every included study. Sixteen studies apply an office threshold of 140/90 mmHg. Three apply the 2017 ACC/AHA threshold of 130/80 mmHg [38,43,44], which admits stage 1 hypertension and therefore a milder case group. One defines cases at the opposite extreme, restricting them to grade 3 hypertension at 180/110 mmHg [41]. One treats blood pressure as a continuous variable and does not dichotomise at all [60]. LaMonte et al. [16] classify baseline status against 120/80 mmHg but define the outcome as physician-diagnosed hypertension treated with medication, which is a clinical rather than a numerical criterion. Four studies state no numeric threshold, relying instead on physician diagnosis or an unspecified reference to national guidelines [4,17,45,47].

The expected consequence, as a matter of measurement theory, is dilution: a case group entered at 130/80 mmHg contains participants closer to the comparator, so any true difference in microbial composition should appear smaller than in a corpus of studies recruiting at 140/90 mmHg. Two qualifications are needed before that expectation is applied to the present findings. The first is technical. Huart et al. [46] also use a 130/80 mmHg cutoff, but apply it to a 24 h ambulatory mean, and ambulatory means run systematically below office readings; 130/80 mmHg on ambulatory monitoring is the recognised equivalent of 140/90 mmHg in the office [61], not a more permissive criterion. Threshold numbers are therefore not comparable across measurement methods, and a study cannot be classified as lenient or strict from its cutoff alone. The second qualification concerns the comparator, which varies as much as the case definition and in the same consequential way. Some studies require controls to be below 120/80 mmHg [27,39], one below 115/80 mmHg above the age of 50 [27], and others define the comparator only as the absence of a hypertension diagnosis. Dilution can therefore arise at either end of the contrast, and the width of the separation between groups—not the case threshold in isolation—is what determines the expected effect size.

Whether the predicted dilution is actually present in this corpus cannot be established from the studies available, and it would be misleading to assert it. The threshold a study adopts is not independent of its other features: the three studies using 130/80 mmHg are small, two are from populations represented by no other study here, and one is a within-hypertensive comparison, while the two largest cohorts in the review both use 140/90 mmHg. Case definition is thus confounded with sample size, geography and design, and no contrast in this corpus isolates it. The observation is nonetheless consequential in two respects. It is a further reason for the decision, taken at protocol stage, not to pool: a summary estimate drawn from studies that do not agree on who counts as a case conveys a precision the underlying data do not possess. And it identifies a design that would settle the question directly—applying both thresholds to the same cohort and comparing the resulting effect estimates, which is feasible in any existing study that recorded blood pressure as a continuous variable, and costs nothing but a reanalysis.

If the threshold determines who counts as a case, the measurement determines whether the threshold has been applied to the right quantity, and here the corpus is weaker still. Only two of the thirty observational studies characterise blood pressure by 24 h ambulatory monitoring [46,60]. The remainder rely on office or clinic readings, and the quality of those readings, now documented study by study in Supplementary Table S3, varies more than the shared label suggests. Twelve studies describe a protocol that would satisfy contemporary guidance—a seated rest period, a named validated device, two or three readings at stated intervals, and an average rather than a single value. Four describe the device or the number of readings but not both. Seven do not describe the measurement at all, reporting only that participants were hypertensive. A reader cannot tell, for roughly a quarter of this corpus, whether the exposure that defines the entire analysis rests on one reading or on several.

The consequences of this are worth following through, because they do not all run in the same direction. White-coat and masked hypertension misclassify participants relative to their true blood pressure phenotype, and circadian variation means that a single daytime reading is a noisy estimate of the quantity that matters. Crucially, however, none of these errors is plausibly related to a participant’s microbial composition: no feature of the gut or oral community determines whether a person’s pressure rises in a clinic. The misclassification is therefore non-differential, and non-differential misclassification of a binary outcome attenuates associations toward the null [62]. Applied rigorously, this consideration implies that the associations reported in this corpus are more likely to be underestimates than artefacts. The cost falls elsewhere, and it falls on this review specifically: null results become substantially harder to interpret, because a study that reports no association may simply have measured the exposure too imprecisely to detect one. Several of the findings summarised here are nulls—the repeated absence of alpha-diversity differences most of all—and their evidential weight is correspondingly reduced.

One study in the corpus is positioned to test this directly rather than argue it. Lin et al. [60] measured 24 h ambulatory pressure in 3695 participants and office pressure in a further 2770 from the same cohort, and applied the same adjustment model to both, yielding a within-study estimate of what the measurement method does to the association while holding population, laboratory and analysis constant. No other study in this review permits that comparison, and it is the design that would settle the question at scale. Two changes follow for practice. The first is minimal and immediate: the measurement protocol should be reported, as seven studies here do not report it and its absence cannot be repaired retrospectively. The second is that where ambulatory monitoring is performed, night-time pressure should be analysed in its own right rather than absorbed into a 24 h mean; nocturnal pressure and dipping status are the strongest blood pressure predictors of cardiovascular outcome [63], and the only study in this corpus to distinguish daytime from night-time hypertension is a single male-only cohort of modest size [46].

Three lines of evidence in the corpus bear on causal direction. Faecal transplantation from hypertensive human donors into germ-free mice raised recipient blood pressure [4]; salivary gavage from hypertensive participants exacerbated angiotensin II-induced hypertension in mice, with Veillonella colonising recipient guts [18]; and Mendelian randomisation using host genetic instruments for microbiota composition supports a causal contribution [5]. Taken together these establish that a dysbiotic community is capable of raising blood pressure in a permissive model system, and that at least part of the association is unlikely to be pure reverse causation.

Because this is the point at which microbiome research most often overreaches, it is worth stating precisely what each of these designs licenses. Faecal transplantation into germ-free recipients establishes that a whole community, transferred as a unit, is sufficient to raise blood pressure in that host. It establishes nothing about the necessity or the sufficiency of any member of that community, because no member was tested in isolation. Three features of the model compound the restriction. The recipient is germ-free, an immunological and ecological state with no human counterpart, in which an incoming community meets neither resident competitors nor a matured mucosal immune system; colonisation in that setting is not informative about what the same organisms would do in a conventional gut. The recipient is also a mouse, and human-derived taxa colonise mice selectively, so the community that raises blood pressure in the recipient is a filtered and reassembled version of the donor community rather than a copy of it. Additionally, the readout is a phenotype in that reassembled state, which cannot be mapped back onto the relative abundances measured in the human donor.

What the design does establish is worth keeping, because it is not trivial and it is the reason the experiment appears in this review at all: the direction of the arrow. In a transplantation experiment the microbiota is manipulated and blood pressure is observed, so the finding is not open to the reverse-causation reading that applies to every cross-sectional association in this corpus—that raised blood pressure, or its treatment, or the behaviour that accompanies it, alters the microbiota rather than the other way round. Mendelian randomisation supports the same directional claim by a different route, though its instruments for microbiota composition are weak and taxonomically coarse, so it inherits the same ceiling: it too speaks to community-level causation rather than to any named organism. Neither design, singly or together, converts a taxon-level association into a taxon-level cause.

The designs that would make that conversion are known and largely absent. Mono-colonisation of gnotobiotic animals with a single candidate organism, or with defined consortia assembled from cultured isolates, would test sufficiency at the level of the taxon; targeted depletion or repletion within a conventional community would test necessity. In humans, the discriminating design is interventional, and the corpus contains exactly one example of the general form: a randomised trial of non-surgical periodontal therapy with a blood pressure endpoint, accompanying Mendelian randomisation of the same relationship [57]. That study alters a microbial community deliberately and measures blood pressure as an outcome, which is what the gut compartment lacks entirely. For the present review the consequence is a boundary we have applied throughout and restate here: the experimental evidence licenses the claim that the microbial community is causally upstream of blood pressure in model systems, and licenses no claim about the causal status of *Ruminococcus gnavus*, *Neisseria subflava*, or any other organism named in this synthesis. Those remain associations, replicated in some cases and mechanistically plausible in others, but associations nonetheless.

One organism in the salivary-gavage experiment deserves separate treatment, because it appears in this review with two opposite characters. Veillonella belongs to the group of oral nitrate-reducing commensals that sustains the entero-salivary nitrate–nitrite–nitric oxide circuit, the pathway through which the oral compartment is proposed to lower blood pressure; it is also, in Chen et al. [18], the corpus’s most frequent oral-to-gut transmitter, enriched in hypertensive participants in both compartments. The paradox is sharper than a simple division between beneficial and harmful roles, because it operates within a single function. Nitrate reduction in the mouth is a group-level capacity distributed across several genera, and within this corpus two members of that group move in opposite directions: *Neisseria subflava* is depleted in hypertension and tracks higher salivary nitric oxide, while Veillonella is enriched. Possession of the nitrate-reducing pathway therefore does not predict the direction of a taxon’s association with blood pressure—which is the compartment-level restatement of this review’s central observation, that taxonomic identity is a poor proxy for community function.

A metabolic reading of the discrepancy is available and is worth stating as a hypothesis, with its evidential status made explicit. Veillonella species are asaccharolytic: they cannot ferment carbohydrate and subsist on lactate, which they convert to propionate and acetate [64]. In the oral biofilm this is ordinary community metabolism, consuming streptococcal lactate in a niche where the organism is a normal resident, alongside its contribution to nitrate reduction. Arriving in the gut through swallowed saliva, the same metabolism operates in a compartment where Veillonella is not normally abundant and where lactate is an intermediate rather than a surplus, so the organism would function as an ectopic consumer rather than a resident one. The observations consistent with a harmful role in that setting are that Chen et al. [18] resolved transmission at strain level, by single-nucleotide-variant distance between paired oral and gut metagenomes, identifying *Veillonella parvula* and *Veillonella atypica* among four frequent transmitters; that Veillonella abundance correlated with at least one leukocyte count or ratio in every sample type in that study; and that salivary gavage from hypertensive donors exacerbated angiotensin II-induced hypertension in mice, with Veillonella colonising recipient guts. What these do not establish is attribution. The gavage transferred a whole salivary community, no study in this corpus measured lactate or propionate against Veillonella abundance, and the pro-inflammatory characterisation rests on leukocyte correlations rather than on a demonstrated mechanism. The proposition that one genus is beneficial in the mouth and harmful in the gut is therefore a hypothesis this corpus makes plausible and cannot test. Three straightforward designs would test it: measuring salivary nitrate, nitrite and nitric oxide alongside oral and faecal Veillonella in the same participants, so that the two proposed effects are observed in one individual rather than inferred across studies; comparing oral and gut isolates at strain level to establish whether the same organism occupies both niches, which the SNV approach of Chen et al. [18] makes feasible in existing metagenomic data; and mono-colonisation of gnotobiotic animals with V. parvula, which is the only design in this list capable of isolating the genus from the community it travels with.

What they do not establish is that any specific organism identified in the human observational studies is the operative one. The transplantation experiments transfer whole communities; the Mendelian randomisation instruments are weak and taxonomically coarse, as is generally the case for microbiome genome-wide association data. Meanwhile the observational evidence carries a confounder this review cannot resolve: most hypertensive participants are taking antihypertensive medication, and roughly a quarter of marketed non-antibiotic drugs inhibit the growth of at least one commensal gut species in vitro [65], yet only one study in the corpus adjusts for medication at the level of pharmacological class (Table S4)—a concern given that population-based analyses relating gut microbiota to prescription drug use identify antihypertensive classes among the medications most strongly associated with compositional differences [66]. The one study designed around treatment status compared controlled with uncontrolled hypertension rather than hypertensive with normotensive subjects [43], which is informative about treatment response but leaves the primary question untouched. The causal architecture in Figure 1 should therefore be read as a framework organising plausible mechanisms, not as a set of demonstrated pathways.

The scale of that gap can now be stated rather than asserted. Table 6 sets out how the corpus handled antihypertensive therapy, with the per-study detail—proportion treated, method of ascertainment and analytical handling—given in Table S4. Of the thirty observational studies, only four adjust for medication in any form, and only one—Palmu et al. [33], using national prescription-register data—does so by pharmacological class; two others enter a single binary term [25,36] and one an undifferentiated medicinal-use variable [45]. Palmu et al. [33] define medication use from all prescription purchases in the four months before baseline, enter diuretics, beta-blockers, calcium-channel blockers and renin-angiotensin-system inhibitors as separate terms, and are also alone in reporting the treated proportion broken down by class (18.0% of the cohort overall). Seven studies remove the problem at source by restricting recruitment to treatment-naive patients [4,16,34,37,38,42,50], three of them citing the known effect of antihypertensive drugs on the microbiota as the explicit reason for that exclusion. Nine include treated and untreated participants together with no medication term in the analysis, and five do not report treatment status at all. The remaining five studies sit at the opposite extreme from the treatment-naive designs: their entire hypertensive group is on therapy [8,40,43,48,51], in two cases for more than a decade and with blood pressure pharmacologically controlled at the time of sampling [40,51]. These two groups of studies are not addressing the same question—one contrasts untreated hypertension with normotension, the other contrasts treated, often controlled, hypertension with the absence of hypertension—yet both contribute to the same compartment-level syntheses, and the direction of the taxonomic findings is largely shared across them. Where the confounder has been examined directly rather than adjusted away, it has not accounted for the signal: two studies tested microbiota composition between medication users and non-users and found no difference [18,19], and a third reported that a stratified analysis separating hypertension from treatment was not feasible because only thirty of its one hundred and eighty-three hypertensive participants were untreated [36]. That last figure is the more general obstacle. In population-based samples, untreated hypertensive adults are too few to support the comparison that would settle the question, which is why the medication confounder persists in this literature as a structural feature of the available populations rather than an oversight of individual studies.

Diet is the second confounder of the same class as medication, and the corpus handles it no better. Of the gut-compartment studies, exactly one adjusts for sodium: Lin et al. [60] estimate 24 h sodium excretion from spot urine by the Kawasaki formula and carry it, together with dietary fibre and total energy, into every primary model through directed-acyclic-graph-based covariate selection. Two adjust for fibre. The second is Louca et al. [27], who repeated their analysis with non-starch polysaccharide intake as a covariate and report that the results were unchanged. Three further studies adjust for a composite diet-quality score rather than for either nutrient specifically—the Healthy Eating Index in LaMonte et al. [16], an a priori diet quality score in Sun et al. [36], and a healthy-food-choices index in Yeo et al. [34]—which controls for dietary pattern in aggregate but cannot isolate the two exposures with the most direct physiological route to blood pressure and to microbial substrate supply. Several studies collected dietary data and did not use it: the HELIUS food-frequency questionnaire derived sodium in grams, yet sodium enters no model [25]; a validated Chinese questionnaire was administered and nutrient intakes compared between groups without adjustment [19]; three-day dietary recalls were collected and not carried forward [51]. Most of the remaining studies record no dietary information at all.

The inference this invites must be stated in its strongest form before it can be answered. Sodium raises blood pressure directly, and fibre is the principal substrate of the short-chain fatty acid producers whose depletion is among this review’s convergent findings. A population eating a high-sodium, low-fibre diet would therefore be expected to show both higher blood pressure and depletion of Faecalibacterium and Roseburia, with no causal relation between the two. On the evidence of covariate adjustment alone, that explanation cannot be excluded for most of this corpus, and it would be wrong to suggest otherwise.

Three observations bear against it, and they are worth separating by strength. The weakest is that the one study to test fibre adjustment directly found its associations unchanged [27], which is reassuring for two genera in one cohort and no more. The second is that the most thoroughly diet-adjusted study in the corpus is also one of the largest and reports associations after adjustment for sodium, fibre and energy simultaneously [60]. The strongest is structural rather than statistical. In faecal transplantation into germ-free mice, recipients receive the donor community while eating standard chow, and blood pressure rises nonetheless [4]; the same holds for salivary gavage [18]. This does not show that diet is irrelevant—the donor’s diet is precisely what shaped the community being transferred—but it distinguishes two hypotheses that covariate adjustment cannot separate. If diet were a confounder in the strict sense, causing dysbiosis and hypertension by independent routes, transferring the community without the diet should not transfer the phenotype. That it does is consistent with diet acting upstream of the microbiota, with the community as the proximate mediator rather than a bystander. Mendelian randomisation, whose instruments are host genetic variants and therefore not shaped by dietary intake, points the same way [5]. The conclusion we draw is narrower than either extreme: the observational literature cannot currently separate diet-driven from microbiota-driven effects, the experimental literature indicates that the microbial community carries at least part of the effect independently of concurrent diet, and quantitative adjustment for sodium and fibre—feasible wherever a food-frequency questionnaire or a spot urine sample already exists—should become routine rather than exceptional.

Three recent syntheses cover parts of the same ground [22,23,67]. Avram et al. [22] pooled gut-compartment studies into summary estimates; Al-Maweri et al. [23] reviewed the oral compartment narratively and reported a partially different set of convergent organisms, including Aggregatibacter, Kingella, Lautropia and Leptotrichia, with Atopobium, Prevotella and Veillonella surviving restriction to studies controlling for false discovery and confounders. The present review differs from the first in declining to pool. That decision is not a claim that the earlier meta-analysis was performed incorrectly, but a judgement that a summary estimate drawn from studies differing in compartment, in whether the outcome is prevalent or incident hypertension, and in whether the effect is expressed as a beta coefficient, an odds ratio or a hazard ratio, conveys a precision the underlying data do not possess. It differs from the second, and from the general gut-focused review of Tsiavos et al. [67], in placing the oral findings alongside gut and fungal evidence, which is what makes the cross-compartment structural observation visible at all. The limited overlap between the organisms highlighted here and those highlighted by Al-Maweri et al. is itself a result worth reporting. Two reviews of substantially the same oral literature, applying different inclusion criteria and different convergence thresholds, arrive at different shortlists. That is a signature of an evidence base in which analytic choices still determine conclusions more than the underlying biology does. Three priorities follow from the above. First, network and co-abundance structure should be tested as a prespecified primary outcome rather than as a secondary description, as it is the one signal that has appeared across compartments and kingdoms and it is measurable in cohorts that already exist. Second, studies are needed in populations that the corpus does not currently reach—African, South Asian and Latin American—not as an equity gesture but because the existing findings cannot be interpreted without them. Third, and most tractably, adjustment for antihypertensive medication class and for dietary sodium and fibre intake should become standard rather than exceptional; the small number of studies that have done so report attenuated associations, which is exactly what would be expected if part of the observed signal reflects treatment and diet rather than disease.

## 5. Conclusions

Across gut, oral and fungal compartments, the microbiota is consistently different in hypertensive individuals, and consistently different in ways that do not replicate well between studies. This review identifies a small number of organisms with cross-population support, of which the *Ruminococcus gnavus* group is the most convergent, and one biochemical measure—salivary nitric oxide—that carries higher certainty than any taxonomic finding. It also identifies a structural signature, the collapse of microbial co-correlation networks, that recurs across compartments and kingdoms and that conventional differential-abundance analysis is not designed to detect. The certainty of evidence for each individual taxon is low to very low. This is a statement about taxon-level attribution and not about the microbiota–hypertension relationship as such, which is supported by consistent community-level differences and by experimental transfer models, and it is less discouraging than it may appear: it locates the problem not in the biological hypothesis, which remains plausible and is supported by experimental transfer models, but in the design and reporting of the human studies that have tested it. Larger prospective cohorts with adequate adjustment, analytic attention to community structure rather than to species lists alone, and recruitment beyond the handful of countries currently represented would be sufficient to move several of these findings out of the very-low-certainty category. Until then, no microbial taxon should be presented as an established marker of hypertension, and none is ready to inform clinical practice.

## Figures and Tables

**Table 1:** Summary of taxonomic and biochemical convergence in the oral compartment.

Taxon/Finding	Direction	Independent Studies	Populations	Convergence Status
*Neisseria subflava*	↓ (protective, nitrate-reducing)	2	USA (LaMonte, prospective) [16] + Italy (Barbadoro, case–control) [17]	Convergent—cross-method (16S vs. qPCR), cross-design (prospective vs. case–control), same proposed mechanism
Salivary nitric oxide (biochemical, not taxonomic)	↓ associated with hypertension	1	Italy (Barbadoro) [17]	Single-study; direct biochemical confirmation of the nitrate-reduction mechanism underlying the *N. subflava* signal
*Veillonella* (oral–gut transmitter)	↑ (harmful, stably enriched)	1 (causal/experimental)	China (Chen et al.) [18]	Single-study; causal design (salivary gavage); qualitatively echoed in Al-Maweri et al.’s benchmark review (Section 3.1.8)
10 OTUs (unspecified species)	↑ (harmful, pre-correction only)	1	USA (LaMonte) [16]	Not significant after Benjamini–Hochberg correction—reported for transparency, not as a convergent or independently reportable signal
5 OTUs (unspecified species, incl. *N. subflava*)	↓ (protective, pre-correction only)	1	USA (LaMonte) [16]	Not significant after Benjamini–Hochberg correction, except *N. subflava* considered separately above given independent qPCR confirmation
*Streptococcus mutans*	↓ (protective)	1	Italy (Barbadoro) [17]	Single-study qPCR finding
*Treponema denticola*, *Aggregatibacter actinomycetemcomitans*	↑ (harmful)	1	Italy (Barbadoro)	Single-study qPCR findings

Only *Neisseria subflava* meets the pre-specified two-independent-study convergence threshold (Section 2) within the oral compartment—a markedly sparser convergence picture than the gut compartment’s four convergent taxa (Section 3.2.10), consistent with the oral compartment’s smaller and more methodologically heterogeneous evidence base (Section 3.1.1).

**Table 2:** Overview of studies cited in this document. Ref. = reference number in this document (matches the numbered References list). Role: Diversity = contributed diversity data; Taxa = contributed taxon-level data; Causal = causal-evidence synthesis; Benchmark = retained narratively, excluded from all pooling; Within-category = excluded from primary normotensive-vs-hypertensive pooling, addressed in a dedicated sub-analysis.

Ref.	Study	Design	Compartment	Population (N)	Role in This Review
[4]	Li et al. 2017	Case–control + FMT (germ-free mice)	Gut	China	Causal
[5]	Li et al. 2023	Mendelian randomisation	Gut	—	Causal
[16]	LaMonte et al. 2022	Prospective (10.4 y)	Oral	USA—Buffalo, WHI (N = 735 prospective, 1215 total)	Diversity, taxa
[17]	Barbadoro et al. 2021	Case–control	Oral	Italy—Ancona (N = 48)	Taxa (biochemical)
[18]	Chen et al. 2023	Multi-compartment, causal (salivary gavage)	Oral + Gut	China—Shanghai	Causal
[19]	Zou et al. 2022	3-cohort (pre-HTN/HTN/normotensive)	Gut mycobiome	China	Taxa (mycobiome)
[20]	Chen et al. 2023	Case–control	Mycobiome, multi-compartment	China—Shanghai (N = 60: 36 HTN, 24 controls)	Taxa (mycobiome)
[21]	Gao et al. 2025	Cross-cohort, multi-cohort	Gut + mycobiome	Multiple cohorts	Benchmark
[22]	Avram et al. 2025	Systematic review + meta-analysis	Gut	—	Benchmark
[23]	Al-Maweri et al. 2025	Systematic review	Oral	— (17 studies, N = 6007 pooled)	Benchmark
[25]	Verhaar et al. 2020	Cross-sectional	Gut	Netherlands—HELIUS (N ≈ 4600+)	Diversity, ethnicity
[26]	Deschasaux et al. 2018	Cross-sectional	Gut	Netherlands—HELIUS	Cohort description
[27]	Louca et al. 2021	Cross-sectional + independent replication	Gut	UK—TwinsUK + PREDICT-1, female-only (N = 871 + 448)	Diversity, taxa, sex
[33]	Palmu et al. 2020	Cross-sectional	Gut	Finland—FINRISK (N = 6953)	Diversity
[34]	Yeo et al. 2026	Prospective (20 y)	Gut	Finland—FINRISK (N = 3311)	Diversity
[35]	Verhaar et al. 2026	Prospective (9.5 y/6.2 y)	Gut	Netherlands—HELIUS (N = 4792/3511)	Diversity, taxa, ethnicity
[36]	Sun et al. 2019	Cross-sectional	Gut	USA—CARDIA (N = 529)	Diversity
[40]	Cheng et al. 2025	Case–control	Gut	China—Lishui, Zhejiang (N = 205: 153 HTN, 52 controls)	Diversity, taxa
[48]	Liu et al. 2026	Cross-sectional, geographic comparison	Gut	China—Daqing vs. Haikou (N = 60)	Within-category
[43]	Valdez-Palomares et al. 2025	Cross-sectional	Gut	Mexico—Mexico City (N = 240)	Within-category
[32]	Guo et al. 2026	Cross-sectional	Oral	China—Guangzhou	Within-category
[49]	Liu et al. 2025	Cross-sectional, co-abundance network	Gut	China—Guangdong Gut Microbiome Project (N = 6999)	Within-category

**Table 4:** Summary of mycobiome findings and their status in this review.

Finding	Direction/Description	Independent Studies	Populations	Status
*Exophiala* spp.	Altered, species-dependent, shared across saliva, plaque, and faeces	1 (cohort shared with [18])	China (Shanghai)	Single cohort; not convergent
Eleven fungal species correlated with SBP/DBP	Seven positive, four negative, compartment-specific	1 (cohort shared with [18])	China (Shanghai)	Single cohort; not convergent
Fungal dysbiosis at the pre-hypertensive stage	Present before diagnosis; predicts immunoglobulin light-chain dysregulation	1	China	Single study; distinct taxa from [20]
Collapse of fungal co-correlation networks	22 → 3 (saliva), 23 → 3 (plaque), 26 → 1 (faeces)	1, echoed in gut bacteriome network analyses	China (Shanghai)	Structural signature; see Section 3.3.4 and Section 4
Alpha diversity	No significant difference in saliva or plaque; trend in faeces (*p* = 0.09)	1	China (Shanghai)	Null
Beta diversity	Significant in saliva and plaque; not in faeces	1	China (Shanghai)	Single cohort
Reproducibility of mycobiome alteration	No consistent alteration across cohorts	1 (multi-cohort)	Multiple	Contradicts the above

**Table 5:** Certainty of evidence (GRADE) for the key findings of this review. All bodies of evidence start at low certainty because all constituent studies are observational.

Finding	Studies (Populations)	Effect as Reported	Downgraded for	Rated Up for	Certainty
*Ruminococcus gnavus* group ↑ (gut)	3 (China ×2 cross-sectional; Netherlands prospective)	OR 1.07 (1.01–1.14), BH-corrected, fully adjusted [35]; direction concordant in two Chinese cohorts	Imprecision (−1): interval abuts the null; effect small in absolute terms	—	Very low
*Neisseria subflava* ↓ (oral)	2 (USA prospective 16S; Italy case–control qPCR)	HR 0.89 [16]; 9091 ± 5481 vs. 4791 ± 4349, *p* < 0.001 [17]	Risk of bias (−1): prospective HR did not survive BH correction; n = 48 with group imbalance. Imprecision (−1)	—	Very low
Faecalibacterium ↓ (gut)	4 (China ×3, Brazil)	Direction only; effect sizes not extractable	Risk of bias (−1): cross-sectional, multiplicity correction undocumented in three of four studies	—	Very low
Roseburia ↓ (gut)	5 (China ×4, Brazil, plus [40])	Direction only for most; *R. hominis* protective for MACE/MACE+ [35]	Risk of bias (−1). Indirectness (−1): Mexican and HELIUS contributions from a within-hypertensive comparison and a cardiovascular outcome respectively	—	Very low
Klebsiella ↑ (gut)	2 (China)	Direction only	Risk of bias (−1). Inconsistency (−1): direction reversed in a United States study [8]. Indirectness (−1): no replication outside east Asia	—	Very low
Ruminiclostridium 6 ↓ and Erysipelotrichaceae UCG-003 ↑ (gut)	1 publication, internal discovery + replication (UK)	β −0.31 (−0.5 to −0.13) and β 0.46 (0.3–0.62), author meta-analysed [27]	Inconsistency not assessable (single research group). Indirectness (−1): exclusively female	—	Very low
Lower alpha diversity (gut)	5 with extractable estimates plus 1 narrative null	OR 0.75 (0.60–0.94) [36]; OR 0.91 (0.86–0.96) [33]; β −0.05 (−0.095 to −0.004) [27]; HR 0.99 (0.92–1.07), null [34]	Inconsistency (−1): the longest prospective study reports a precise null after full adjustment. Imprecision (−1)	—	Very low
Mycobiome dysbiosis	2 cohorts positive vs. 1 multi-cohort study negative [21]	Beta-diversity separation in saliva and plaque but not faeces; network collapse	Inconsistency (−1): contradicted by a purpose-designed cross-cohort study. Imprecision (−1): n = 60	—	Very low
Salivary nitric oxide ↓ (oral, biochemical)	1 (Italy case–control)	57.5 ± 19.6 vs. 165.8 ± 61.7 µmol/L, *p* = 0.023; OR 0.33 (0.13–0.86) [17]	Inconsistency not assessable (single study). Imprecision (−1): n = 48	Large effect (+1): near three-fold difference in means with a consistent multivariable odds ratio	Low

**Table 6:** Handling of antihypertensive medication across the thirty observational studies included in this review. Categories are mutually exclusive. Gao et al. [21] is retained as a narrative benchmark and Li et al. [5] is a Mendelian randomisation study without participant-level medication data; neither is listed. Per-study detail is given in Table S4.

Handling of Antihypertensive Medication	n	Studies
Treatment-naive by design (treated participants excluded, or analysis restricted to participants free of medication at baseline)	7	[4,16,34,37,38,42,50]
Adjusted by pharmacological class	1	[33]
Adjusted as a single, undifferentiated covariate	3	[25,36,45]
Treated and untreated participants both present, with no medication term in the analysis	9	[18,19,27,35,39,44,46,49,60]
Entire hypertensive group on therapy (contrast is with treated, in two cases pharmacologically controlled, hypertension)	5	[8,40,43,48,51]
Treatment status not reported	5	[17,20,32,41,47]

## Data Availability

No new data were created in this study. All data supporting the findings are drawn from the published reports cited in the References and are summarised within the article and its Supplementary Materials. The full search strategies, the per-study risk-of-bias judgements, the methodological characteristics of the included studies and the data extraction template are provided as Supplementary Tables S1–S3 and Supplementary Form S1; the record flow is presented as Figure 2 in the main text.

## Supplementary Materials

Thefollowingsupporting information can be downloaded at https://www.mdpi.com/article/10.3390/ijms27188029/s1.
