# Supplementary material for: Gut, Oral, and Fungal Microbiota in Hypertension: A Multi-Compartment Systematic Review
Source: Int J Mol Sci. 2026 Sep 9;27(18):8029. doi: 10.3390/ijms27188029 (PMC13607618; doi:10.3390/ijms27188029)
Supplement: Supplementary file 1 [file ijms-27-08029-s001.zip › Form S1.pdf]

Form S1. Data extraction template

Systematic review of gut, oral and fungal microbiota in relation to hypertension. Standardised form used by both reviewers for independent data extraction from each included report.

One form is completed per included report by each of the two reviewers independently. Fields left unreported by the source study are recorded as "not reported" rather than left blank, so that reporting gaps can be distinguished from extraction omissions. Completed forms were compared field by field; discrepancies were checked against the source and, if persistent, resolved by the third reviewer.

Domain	Field	Entry
**Record identification**	Extraction ID (Rayyan record number)	
	First author, year of publication	
	DOI / PMID	
	Journal, volume, pages	
	Language of publication	
	Reviewer initials and date of extraction	
	Second reviewer initials and date; discrepancies resolved by	
**Study design**	Design (case–control / cross-sectional / prospective cohort / Mendelian randomisation / nested experimental component)	
	Cohort or consortium name, if any	
	Shares a cohort with another included report? If yes, which	
	Comparator (normotensive / no hypertension at baseline / within-hypertensive category — specify)	
	Follow-up duration (prospective designs)	
**Population**	Country and recruitment setting	
	Total N; N per group	
	Age (mean ± SD or median, IQR)	
	Sex distribution; single-sex design (yes/no)	
	Ethnicity, as reported; ethnicity-stratified analysis (yes/no)	
	BMI; diabetes, dyslipidaemia and CKD prevalence	
	Diet assessment performed (yes/no); instrument	
**Exposure to hypertension**	Blood pressure ascertainment method (24-h ABPM / home / office single / office repeated / self-reported / registry or ICD code)	
	Number of readings and visits; device and cuff validation, if reported	

Domain	Field	Entry
	Hypertension definition and threshold (≥140/90, ≥130/80, other — specify guideline)	
	Antihypertensive treatment: proportion treated; classes recorded (yes/no); adjusted for (yes/no)	
	Pre-hypertensive stratum present (yes/no)	
**Microbiota methodology**	Compartment(s) sampled (faeces / saliva / subgingival plaque / supragingival plaque / other)	
	Collection, transport and storage conditions; stabilising buffer	
	DNA extraction kit	
	Method (16S rRNA amplicon / ITS / shotgun metagenomics / targeted qPCR)	
	Hypervariable region (16S) or ITS region; primers	
	Sequencing platform and read length	
	Bioinformatic pipeline and version; reference database and version	
	OTU or ASV clustering; rarefaction depth	
	Lowest taxonomic rank reported	
**Metabolite data**	Metabolites measured (SCFA / TMAO / nitric oxide / bile acids / other)	
	Measurement matrix (faeces / plasma / serum / urine / saliva) — mandatory	
	Analytical platform (GC/MS, LC-MS/MS, NMR, colorimetric assay) — mandatory; values from different platforms are not directly comparable	
	Units as reported	
**Results — diversity**	Alpha diversity indices reported; direction; effect estimate with 95% CI and P; extractable (yes/no)	
	Beta diversity: distance metric, test (PERMANOVA/ADONIS), R^2, P	
**Results — taxa**	Number of taxa tested; taxonomic rank of testing	
	Differential-abundance method and software	
	Correction for multiple comparisons (method; number of taxa significant before and after)	
	Taxa increased in hypertension, with effect estimates and 95% CI	
	Taxa decreased in hypertension, with effect estimates and 95% CI	
	Network or co-abundance analysis performed (yes/no); result	
**Results — other**	Functional or pathway analysis (yes/no); result	

Domain	Field	Entry
	Cardiovascular outcomes reported (MACE and similar)	
	Diagnostic or predictive modelling (AUC, panel composition)	
**Adjustment**	Covariates in the primary adjusted model	
	Subgroup analyses by sex; by ethnicity; by enterotype	
**Appraisal**	Newcastle–Ottawa version applied; total stars; overall judgement	
	Funding source and declared conflicts of interest of the source study	
	Data and code availability of the source study	
**Reviewer notes**	Discrepancies between reviewers and how resolved	
	Queries sent to study authors; response	
	Free-text notes	
