# Supplementary material for: Gut, Oral, and Fungal Microbiota in Hypertension: A Multi-Compartment Systematic Review
Source: Int J Mol Sci. 2026 Sep 9;27(18):8029. doi: 10.3390/ijms27188029 (PMC13607618; doi:10.3390/ijms27188029)
Supplement: Supplementary file 1 [file ijms-27-08029-s001.zip › Supplementary_Materials.pdf]

Supplementary Materials

Gut, Oral, and Fungal Microbiota in Hypertension: A Multi-Compartment Systematic Review

Carini F, Sorce A, Ciuppa ME, David S, Giammanco M, di Carlo P, Evola S, Tomasello G, Mulè G, Carollo C

Contents

Table S1. Search strategies and records retrieved, by database

Table S2. Risk of bias of the included studies (Newcastle–Ottawa Scale)

Table S2a. Cohort studies   Table S2b. Case–control studies

Table S2c. Cross-sectional studies (Modesti adaptation)   Table S2d. Designs outside the scope of the Newcastle–Ottawa Scale

Table S3. Methodological characteristics of the included studies, including blood pressure ascertainment

Table S4. Handling of antihypertensive medication across the included studies

Form S1. Data extraction template

Checklist S1. PRISMA 2020 checklist

Checklist S2. Synthesis Without Meta-analysis (SWiM) reporting checklist

Table S1. Search strategies and records retrieved, by database

PubMed / MEDLINE

**Interface / platform**	NCBI PubMed (https://pubmed.ncbi.nlm.nih.gov)
**Date searched**	__ June 2026 (inception to 30 June 2026)
**Filters and limits**	None. No species, article-type, publication-date or language filter was applied at any point. Automatic Term Mapping left enabled.
**Search string, as run**	#1 "Microbiota"[Mesh] OR "Gastrointestinal Microbiome"[Mesh] OR "Mycobiome"[Mesh] OR "Dysbiosis"[Mesh] OR "Metagenomics"[Mesh] OR "Metagenome"[Mesh] OR "RNA, Ribosomal, 16S"[Mesh] OR "Bacteria"[Mesh:NoExp] OR "Fungi"[Mesh:NoExp] OR microbiot*[tiab] OR microbiome*[tiab] OR microflora[tiab] OR "microbial community"[tiab] OR "microbial communities"[tiab] OR bacteriome*[tiab] OR mycobiome*[tiab] OR mycobiot*[tiab] OR "fungal community"[tiab] OR "fungal communities"[tiab] OR dysbiosis[tiab] OR dysbiotic[tiab] OR metagenom*[tiab] OR metataxonom*[tiab] OR "16S rRNA"[tiab] OR "16S ribosomal"[tiab] OR "18S rRNA"[tiab] OR "internal transcribed spacer"[tiab] OR ITS1[tiab] OR ITS2[tiab] OR "amplicon sequencing"[tiab] OR "shotgun metagenomic"[tiab] OR "short chain fatty acid"[tiab] OR "short-chain fatty acids"[tiab] OR SCFA[tiab] OR SCFAs[tiab] OR "trimethylamine N-oxide"[tiab] OR TMAO[tiab] OR "faecal microbiota transplantation"[tiab] OR "fecal microbiota transplantation"[tiab]<br><br>#2 "Hypertension"[Mesh] OR "Prehypertension"[Mesh] OR "Blood Pressure"[Mesh] OR "Arterial Pressure"[Mesh] OR "Blood Pressure Monitoring, Ambulatory"[Mesh] OR "Antihypertensive Agents"[Mesh] OR hypertens*[tiab] OR prehypertens*[tiab] OR "pre-hypertension"[tiab] OR normotens*[tiab] OR "blood pressure"[tiab] OR "high blood pressure"[tiab] OR "elevated blood pressure"[tiab] OR "raised blood pressure"[tiab] OR "systolic pressure"[tiab] OR "diastolic pressure"[tiab] OR "arterial pressure"[tiab] OR "ambulatory blood pressure"[tiab] OR ABPM[tiab]<br><br>#3 #1 AND #2
**Records retrieved**	2,338

Embase

**Interface / platform**	Elsevier Embase.com (Emtree, 2026 edition)
**Date searched**	__ June 2026 (inception to 30 June 2026)
**Filters and limits**	None. Conference abstracts and animal records were retained at the identification stage and removed at screening against the eligibility criteria, rather than by a database limit.
**Search string, as run**	#1 'microbiota'/exp OR 'intestine flora'/exp OR 'mouth flora'/exp OR 'mycobiome'/exp OR 'dysbiosis'/exp OR 'metagenomics'/exp OR 'RNA 16S'/exp OR (microbiot* OR microbiome* OR microflora OR bacteriome* OR mycobiome* OR mycobiot* OR 'microbial community' OR 'fungal community' OR dysbiosis OR dysbiotic OR metagenom* OR metataxonom* OR '16S rRNA' OR '18S rRNA' OR 'internal transcribed spacer' OR its1 OR its2 OR 'amplicon sequencing' OR 'shotgun metagenomic' OR 'short chain fatty acid' OR scfa OR 'trimethylamine n oxide' OR tmao OR 'faecal microbiota transplantation' OR 'fecal microbiota transplantation'):ti,ab,kw<br><br>#2 'hypertension'/exp OR 'prehypertension'/exp OR 'blood pressure'/exp OR 'ambulatory blood pressure monitoring'/exp OR 'antihypertensive agent'/exp OR (hypertens* OR prehypertens* OR normotens* OR 'blood pressure' OR 'high blood pressure' OR 'elevated blood pressure' OR 'systolic pressure' OR 'diastolic pressure' OR 'arterial pressure' OR 'ambulatory blood pressure' OR abpm):ti,ab,kw

	#3 #1 AND #2
**Records retrieved**	1,818

Scopus

**Interface / platform**	Elsevier Scopus (https://www.scopus.com)
**Date searched**	__ June 2026 (inception to 30 June 2026)
**Filters and limits**	None. No document-type, subject-area or language limit applied.
**Search string, as run**	`TITLE-ABS-KEY (microbiot* OR microbiome* OR microflora OR bacteriome* OR mycobiome* OR mycobiot* OR "microbial community" OR "fungal community" OR dysbiosis OR dysbiotic OR metagenom* OR metataxonom* OR "16S rRNA" OR "18S rRNA" OR "internal transcribed spacer" OR "amplicon sequencing" OR "shotgun metagenomic" OR "short-chain fatty acid*" OR SCFA OR "trimethylamine N-oxide" OR TMAO OR "faecal microbiota transplantation" OR "fecal microbiota transplantation")`<br>`AND TITLE-ABS-KEY (hypertens* OR prehypertens* OR normotens* OR "blood pressure" OR "systolic pressure" OR "diastolic pressure" OR "arterial pressure" OR "ambulatory blood pressure" OR ABPM)`
**Records retrieved**	1,987

Web of Science Core Collection

**Interface / platform**	Clarivate Web of Science; editions SCI-EXPANDED, SSCI, A&HCI, CPCI-S, ESCI; timespan: all years
**Date searched**	__ June 2026 (inception to 30 June 2026)
**Filters and limits**	None. No document-type or language refinement applied.
**Search string, as run**	`#1 TS=(microbiot* OR microbiome* OR microflora OR bacteriome* OR mycobiome* OR mycobiot* OR "microbial community" OR "fungal community" OR dysbiosis OR dysbiotic OR metagenom* OR metataxonom* OR "16S rRNA" OR "18S rRNA" OR "internal transcribed spacer" OR "amplicon sequencing" OR "shotgun metagenomic" OR "short chain fatty acid*" OR SCFA OR "trimethylamine N oxide" OR TMAO)`<br><br>`#2 TS=(hypertens* OR prehypertens* OR normotens* OR "blood pressure" OR "systolic pressure" OR "diastolic pressure" OR "arterial pressure" OR "ambulatory blood pressure" OR ABPM)`<br><br>`#3 #1 AND #2`
**Records retrieved**	1,916

Table S2. Risk of bias of the included studies (Newcastle–Ottawa Scale)

Risk of bias was assessed with the Newcastle–Ottawa Scale in the version matching each design, using the adaptation of Modesti et al. for cross-sectional studies. Because the exposure of interest is microbiota composition rather than a discrete exposure, the selection items referring to “exposed” and “non-exposed” cohorts were operationalised as the higher- and lower-abundance strata, or as the hypertensive and normotensive groups, as appropriate to each report; this operationalisation is recorded for each study in the notes column. Each study was assessed independently by two reviewers, with a third resolving disagreement.

Scoring. One point per item met, except comparability — and, for cross-sectional studies, ascertainment of exposure and assessment of the outcome — which may receive up to two. Overall judgement follows the AHRQ thresholds for cohort and case–control studies, and the thresholds in current use for the Modesti adaptation (9–10 very good, 7–8 good, 5–6 satisfactory, 0–4 unsatisfactory).

Status of the entries. Item-level scores were assigned from the full text of every included report. Totals and overall judgements are now reported for every study. For the nine-point cohort and case–control scales the overall judgement follows the AHRQ domain-based conversion (Agency for Healthcare Research and Quality, Methods Guide for Effectiveness and Comparative Effectiveness Reviews, Rockville, MD): Good requires three or four stars in Selection, one or two in Comparability, and two or three in Outcome or Exposure; Fair requires two stars in Selection with the same Comparability and Outcome or Exposure requirements; Poor follows from zero or one star in Selection, zero in Comparability, or zero or one in Outcome or Exposure. A study can therefore be rated Poor on a moderate total, and several are, because a single unmet domain is decisive; this is a property of the instrument and is retained deliberately. For the ten-point Modesti adaptation used for cross-sectional designs (Modesti PA, Reboldi G, Cappuccio FP, Agyemang C, Remuzzi G, Rapi S, et al. Panethnic differences in blood pressure in Europe: a systematic review and meta-analysis. PLoS One. 2016;11(1):e0147601), with the rating bands of Herzog R, Álvarez-Pasquin MJ, Díaz C, Del Barrio JL, Estrada JM, Gil Á. Are healthcare workers’ intentions to vaccinate related to their knowledge, beliefs and attitudes? BMC Public Health. 2013;13:154, totals of nine or ten are rated Very good, seven or eight Good, five or six Satisfactory, and four or fewer Unsatisfactory. The final column of each table states the basis for the scores in the row. The accompanying workbook carries the same grid with the item-by-item scoring rubric.

Table S2a. Cohort studies (maximum 9 stars)

Ref	Study	Selection				Comp.	Outcome			Total /9	Overall	Design, and basis for the scores
		S1	S2	S3	S4	C1	O1	O2	O3			
16	LaMonte et al. 2022	1	1	1	1	2	0	1	1	8	Good	Oral; WHI/OsteoPerio, USA; prospective, 10.4 y. Only prospective study of the oral compartment. Fifteen OTUs significant before correction; none survived Benjamini–Hochberg correction. Alpha diversity reported as P-values on box plots only, without means, SD or effect estimates. — Scored from the full text (LaMonte 2022, J Am Heart Assoc 11:e021930). S1: WHIOS enrolled 2,249 women from the western New York community and neither periodontal status nor blood pressure was an inclusion criterion for either study. S2: exposed and unexposed are the same cohort, stratified on continuous bacterial abundance. S3: 16S rRNA V3-V4 on Illumina MiSeq, standardised paper-point sampling, positive and negative controls, plate randomisation against batch effects. S4: the prospective analysis is restricted to the 735 women without prevalent diagnosed and treated hypertension at baseline. C1: model 3 adjusts for age, race and ethnicity, education, neighbourhood socioeconomic status, self-rated health, treated diabetes, diet quality, physical activity and statin use. CAVEAT for C1: the two covariates that differed most between incident cases and non-cases, BMI (24.9 vs 26.2, P<0.001) and smoking (P<0.001, their Table S1), appear in none of the three models and are handled only by stratification. O1 scores 0 and is the contested item: baseline blood pressure is measured to a validated protocol (mercury sphygmomanometer, cuff sized to arm circumference, 5 min seated rest, two readings >=2 min apart, averaged, Korotkoff I and V), but the INCIDENT outcome is self-reported on an annual questionnaire asking whether a doctor has prescribed pills for high blood pressure. The counter-argument for scoring 1 is the reported validation, kappa=0.84 against Medicare claims and kappa=0.86 test-retest; this is the item on which the two reviewers are most likely to diverge and

Ref	Study	Selection				Comp.	Outcome			Total /9	Overall	Design, and basis for the scores
		S1	S2	S3	S4	C1	O1	O2	O3			
												the divergence should be documented. O2: mean follow-up 10.4 years (SD 5.9, range 0.4-19.2). O3: the fall from 1,215 to 735 is not attrition but the by-design exclusion of 480 women with prevalent treated hypertension; all 735 enter the Cox model with explicit censoring. A stricter reviewer would score 0 because the proportion censored early is not reported - note that O1=0 together with O3=0 drops the outcome domain to 1 and the overall judgement from Good to Poor. Discrepancy in the source: the Results text reports 387 incident cases (52.7%) whereas the abstract and Table 3 report 375 (360+375=735); 375 is internally consistent and was used here.
35	Yeo et al. 2026	**1**	**1**	**1**	**1**	**2**	**1**	**1**	**1**	9	Good	Gut; FINRISK, Finland; prospective, 20 y. Longest follow-up in the corpus. Precise null for Shannon diversity in the fully adjusted model. — Scored from the full text (Yeo 2026, FINRISK 2002). S1: stratified random sample of the Finnish population aged 25-74 drawn from the national population register across six regions. S2: same cohort, stratified on continuous microbial abundance. S3: shallow shotgun metagenomics, Earth Microbiome Project protocol, Illumina HiSeq 4000 2x150 bp, Greengenes2, HUMAnN v3.0.1, 5%/0.1% filter, CLR. S4: 3,263 participants with prevalent hypertension excluded, defined as SBP>=140 or DBP>=90 or self-reported antihypertensive use or a register-based diagnosis before baseline. C1: age, sex, BMI, diabetes types 1 and 2, healthy food choices, cardiovascular disease, smoking. O1 scores 1: incident hypertension by linkage to national registers (Hospital Discharge, Causes-of-Death, special reimbursement code, or at least three ATC purchases of dihydropyridine calcium-channel blockers or thiazide diuretics) - ascertainment independent of the participant, in direct contrast with study 16. O2: baseline 2002, follow-up to 31 December 2022. O3: fixed-date national register follow-up, complete by construction. CAVEATS: the analytic sample follows three successive filters, 13,500 invited to 8,798 examined (65.5%) to 7,355 with a stool sample (80.2%) to 3,311 after exclusions, and 474 were dropped for incomplete covariate data with no imputation; the outcome definition is deliberately specific, so a treated hypertensive on ACE inhibitors, ARBs or beta-blockers is captured only through a diagnosis or reimbursement code, and part of the precise null may be under-ascertainment; faecal samples were mailed overnight in winter, held at -20 C from 2002 until 2017, which is transparently reported but a real pre-analytical concern. NOTE FOR THE MANUSCRIPT: the overview table lists this reference with author order unconfirmed; the first author is Yeo LF. The paper states the Finnish population was more than 99% white in 2002 and that race and ethnicity were not collected - worth citing in the Discussion for the indirectness argument.
36	Verhaar et al. 2026	**1**	**1**	**1**	**1**	**2**	**1**	**1**	**1**	9	Good	Gut; HELIUS, Netherlands; prospective, 9.5 y / 6.2 y. Largest fully Benjamini–Hochberg-corrected taxon-level dataset in the corpus (62/92 ASVs). Formal stratification by ethnicity and by sex. MACE/MACE+ is a cardiovascular surrogate, not a hypertension outcome. — Scored from the full text (Verhaar 2026, HELIUS). S1: random sampling from the Amsterdam municipality registry, ages 18-70, stratified by ethnicity to ensure representation across groups. S2: same cohort, stratified on continuous ASV abundance. S3: 16S rRNA V4 on Illumina MiSeq 2x250 bp, 515F/806R primers, VSEARCH/UNOISE pipeline, SILVA v138.1, rarefied to 15,000 counts, antibiotic users within 3 months excluded. S4: participants with the diagnosis of interest at baseline were excluded separately for each diagnosis. C1: model 2 adjusts for age, sex, BMI, smoking and alcohol. O1 scores 1: new-onset hypertension established at the follow-up VISIT using a validated semi-automatic oscillometric device (Microlife WatchBP Home), the average of two readings after 5 min supine rest, or antihypertensive use. O2: baseline 2011-2015, follow-up 2019-2022. O3 is the contested item and scores 1: only 3,533 of 5,160 participants with a faecal sample attended follow-up (60.2%), far below the 80% threshold, but the scale's alternative criterion is met because the

Ref	Study	Selection				Comp.	Outcome			Total /9	Overall	Design, and basis for the scores
		S1	S2	S3	S4	C1	O1	O2	O3			
												authors compared the baseline characteristics of those lost with those retained. A reviewer applying the numeric threshold would score 0; note that even so the outcome domain stays at 2 and the overall judgement remains Good, so the divergence is documentable but not consequential. CAVEATS: interaction testing was performed at p<0.05 WITHOUT multiple-testing adjustment, whereas the main results are Benjamini-Hochberg corrected - this bears directly on the ethnicity and sex axes of the present review; sodium intake was derived from the food frequency questionnaires but appears in no model, a substantial omission in a hypertension study. DISCREPANCY TO CHECK: the present review states that 92 ASVs were tested against incident hypertension, whereas the Methods report filtering to 225 ASVs for the new-onset analyses (222 for MACE); the origin of the figure 92 needs verifying against the paper's Results.

S1 representativeness of the exposed cohort; S2 selection of the non-exposed cohort; S3 ascertainment of exposure; S4 demonstration that the outcome was not present at baseline; C1 comparability of cohorts on the basis of design or analysis (up to two stars: one for age and sex, one for further cardiometabolic confounders and antihypertensive treatment); O1 assessment of outcome; O2 adequacy of follow-up duration; O3 completeness of follow-up.

Table S2b. Case–control studies (maximum 9 stars)

Ref	Study	Selection				Comp.	Exposure			Total /9	Overall	Compartment, setting, and basis for the scores
		S1	S2	S3	S4	C1	E1	E2	E3			
4	Li et al. 2017	**0**	**0**	**1**	**0**	**0**	**1**	**1**	**0**	3	Poor	Gut; China. Nested germ-free faecal transplantation component assessed separately (Table S2d). — Scored from the full text (Li 2017, human case-control component only). The nested germ-free FMT component is appraised separately in Table S2d using the SYRCLE risk-of-bias tool. S1 scores 0: single centre (Fuwai Hospital, Beijing); no description of the recruitment source or selection method. S2 scores 0: no power calculation; N=60 total (30 EH + 15 PE + 15 NT) without justification. S3 scores 1: healthy controls described as volunteers without cardiovascular disease, not hospital patients. S4 scores 0: blood pressure for cases from medical records; no measurement described to confirm normotension in controls. C1 scores 0: no multivariable model in the human component. E1: 16S V3-V4 on Illumina MiSeq 2x250 bp, primers 341F/806R, QIIME, 97% OTU clustering, RDP classifier against Greengenes. E2: same protocol in both groups. E3 scores 0: non-respondents not described. NOTE: the three-group design (EH, PE, NT) with a gradient across Prevotella and Klebsiella supports a dose-response relationship and is worth citing explicitly in section 3.2.2 alongside the binary EH vs NT comparison. ANIMAL COMPONENT (Table S2d, SYRCLE): germ-free C57BL/6 mice received faecal transplants from EH or NT human donors; systolic BP was significantly higher in mice receiving EH microbiota. SYRCLE: sequence generation unclear, baseline characteristics comparable (same strain/age/sex), allocation concealment and random housing not described, blinding not described; incomplete outcome data low risk; selective reporting low risk. Overall: unclear risk of bias, which is standard for this study type.
8	Kim et al. 2018	**0**	**0**	**0**	**0**	**0**	**1**	**1**	**0**	2	Poor	Gut; USA. Only United States study to examine Klebsiella; direction opposite to the two Chinese studies. — Scored from the full text (Kim 2018, USA). S1 scores 0: single university centre (University of Florida); no description of the recruitment source or selection method. S2 scores 0: no power calculation; N=18+12, the smallest case-control in the corpus, without justification. S3 scores 0: controls recruited at the same centre but not documented as community controls; unclear

Ref	Study	Selection				Comp.	Exposure			Total /9	Overall	Compartment, setting, and basis for the scores
		S1	S2	S3	S4	C1	E1	E2	E3			
												whether they are patients or healthy volunteers. S4 scores 0: hypertension classified from medical records and self-report, not from a measurement performed for this study; the method by which the original blood pressure readings were obtained is not declared. C1 scores 0: no multivariable model; t-test, Mann-Whitney U and correlations only. E1: 16S V3-V4 on Illumina MiSeq, primers 341F/806R, QIIME, 97% OTU clustering, Greengenes 13_8. E2: same protocol in both groups. E3 scores 0: non-respondents not described. IMPORTANT FOR THE VOTE-COUNTING: the 18 cases have a mean blood pressure of 160/100 mmHg WHILE ON ANTIHYPERTENSIVE MEDICATION, meaning they are uncontrolled hypertensives on treatment. This is a different population from the untreated hypertensives in the Chinese studies (Li, Yan). The divergent direction of Klebsiella may reflect this population difference rather than a genuine geographic divergence; this should be stated in the Discussion.
17	Barbadoro et al. 2021	**1**	**0**	**1**	**1**	**1**	**1**	**1**	**0**	6	Good	Oral (saliva, supragingival/subgingival/denture plaque); Ancona, Italy. N=48 with group imbalance (25 normotensive vs 23 hypertensive). Targeted qPCR of eight species, not untargeted sequencing; no community-level diversity index computable by design. — Scored from the full text (Barbadoro 2021, Italy). S1: consecutive series from the dental clinic of Universita Politecnica delle Marche, Ancona, recruited June 2013-March 2015. S2 scores 0: no power calculation; N=48 (25 normotensive + 23 hypertensive) without justification. S3: controls are normotensive patients from the same clinic, not inpatients. S4: the paper states 'the blood pressure was checked in controls during physical examination', so normotension is confirmed by measurement rather than self-report only. For cases, hypertension is a prior physician diagnosis. C1: multiple logistic regression adjusts for BMI, oral hygiene, marital status and age. Smoking was collected with the IPAQ questionnaire and described in the baseline table but was excluded from the final model by stepforward selection at P>=0.30; given the biological plausibility of smoking as a confounder for salivary NO, its exclusion by statistical threshold rather than biological reasoning is a limitation. Antihypertensive drug class and sodium not included. E1: TaqMan qRT-PCR for 8 target species plus a universal probe for total bacterial count, Rotor-Gene 3000, validated primer/probe sets (Kuboniwa 2004, Ciric 2010), duplicate reactions, same thermocycling conditions per organism. Targeted design, objective and calibrated. E2: same protocol and same matrices (saliva, supragingival plaque, subgingival plaque, sub-prosthetic plaque) in both groups. E3 scores 0: non-respondents not described. SALIVARY NO: ELISA kit (R&D Systems), enzymatic conversion of nitrate to nitrite, Griess reaction at 540 nm. Result verified: normotensive 165.77+/-61.7 umol/l vs hypertensive 57.49+/-19.61 umol/l, P=0.023, approximately 3-fold lower in hypertensives, as cited in section 3.1.3 of the review. Antibiotics excluded within 3 months; periodontal treatment within 6 months. Software STATA 15.1.
18	Chen et al. 2023 (J Adv Res)	**1**	**1**	**1**	**1**	**1**	**1**	**1**	**0**	7	Good	Oral + gut; Shanghai, China. Nested salivary-gavage transmission experiment assessed separately (Table S2d). Shares its cohort with [20]. — Scored from the full text (Chen 2023, J Adv Res, Shanghai). The nested salivary-gavage animal component is appraised separately in Table S2d using the SYRCLE risk-of-bias tool. S1: Shanghai Ninth People's Hospital with explicit inclusion/exclusion criteria. S2 scores 1: explicit sample size calculation targeting r=0.25, alpha=0.05, 80% power, minimum N=123; the only case-control in the corpus with a formal power calculation. S3: controls without hypertension from the same institution. S4: ESC/ESH 2018 criteria with measurement performed for this study - three readings at 5-minute intervals, Omron electronic sphygmomanometer, mean of three; normotension in controls verified with the same protocol. C1: smoking excluded by design (exclusion criterion); however, antihypertensive medication is not reported as an exclusion criterion and is not in any statistical model, despite the

Ref	Study	Selection				Comp.	Exposure			Total /9	Overall	Compartment, setting, and basis for the scores
		**S1**	**S2**	**S3**	**S4**	**C1**	**E1**	**E2**	**E3**			
												hypertensive group having a mean duration of 12.3 years, making ongoing treatment very likely. E1: 16S rRNA and metagenomic sequencing on Illumina platforms at Personal Bio Inc., Shanghai; detailed methods (region, primers, pipeline) are in the Supplementary Material not provided here. E2: same protocol and same matrices (saliva, subgingival plaque, faeces) in both groups. E3 scores 0: non-respondents not described. ANIMAL COMPONENT (Table S2d, SYRCLE): male C57BL/6J mice aged 10 weeks, depleted with antibiotic cocktail, then oral gavage with saliva from hypertensive or normotensive donors, followed by subcutaneous Ang II minipump; BP by tail-cuff. SYRCLE: sequence generation not described; baseline comparable (same strain/age/sex); allocation concealment, random housing, blinding not described; incomplete outcome data low risk; selective reporting low risk. Overall: unclear risk of bias. KEY FINDING: SNV matching demonstrates that oral bacterial strains from hypertensive subjects appear in the gut microbiota of the same individuals at higher frequency than in controls; salivary gavage transmits the hypertensive phenotype in the Ang II mouse model. This is the strongest causal evidence for the oral compartment in the corpus.
19	Zou et al. 2022	**0**	**0**	**0**	**0**	**0**	**1**	**1**	**0**	2	Poor	Gut mycobiome; China. Three-group design including a pre-hypertensive stratum. — Scored from the full text (Zou 2022, gut mycobiome). S1 scores 0: no description of recruitment source, method or invitation rate. S2 scores 0: no power calculation; sample size not clearly stated in the available text. S3 scores 0: normotensive controls exist but how they were selected is not described. S4 scores 0: the abstract states participants were classified based on their blood pressure, but no measurement protocol is described anywhere in the available text - no device, no number of readings, no rest period. C1 scores 0: no multivariable model; Wilcoxon rank-sum, PERMANOVA and Pearson correlations only. NOTE: antihypertensive medication users vs non-users were compared in a PERMANOVA and did not differ (R2=0.023, FDR=0.414), which is one of the few sensitivity analyses for medication in this corpus; this does not score C1 but is worth noting in the synthesis. E1: ITS sequencing on Illumina platform - appropriate method for mycobiome profiling; ITS region specified. E2: same protocol across all three groups (pre-HTN, HTN, NT). E3 scores 0: non-respondents not described. THREE-GROUP DESIGN: pre-HTN, HTN and NT; mycobiome differs between pre-HTN and NT (PERMANOVA FDR=0.036), suggesting fungal alterations precede established hypertension - relevant for the causality discussion in section 3.3. Malassezia increased in HTN, Mortierella in NT - verified against section 3.3.2 of the review.
20	Chen et al. 2023 (Microbiol Spectr)	**1**	**0**	**1**	**1**	**0**	**1**	**1**	**0**	5	Poor	Mycobiome, multi-compartment; Shanghai, China. N=60 (36 hypertensive, 24 controls). Shares its cohort with [18]; counted once for convergence. — Scored from the full text (Chen 2023, Microbiol Spectr, Shanghai). Same cohort and clinical site as Chen 2023 J Adv Res [18]: Shanghai Ninth People's Hospital, Department of Cardiology, same inclusion/exclusion criteria, same blood pressure protocol. S1: same as [18] - explicit recruitment criteria at Shanghai Ninth People's Hospital. S2 scores 0: no sample size calculation; N=36+24 without justification (smaller than [18]). S3: controls without hypertension from the same institution. S4: identical to [18] - three readings at 5-minute intervals, Omron electronic sphygmomanometer, sitting position, mean of three. C1 scores 0: no multivariable model; Student's t-test, PERMANOVA, LEfSe and Spearman correlations only; smoking excluded by design. E1 scores 1: SHOTGUN METAGENOMICS on Illumina NovaSeq PE150, Cutadapt v1.2.1, fastp v0.23.2, MEGAHIT assembly, Kraken2 v2.1.2 against NCBI RefSeq + Kaiju v1.8.2 against NCBI NR. The only study in the corpus using shotgun metagenomics for mycobiome profiling (all others use ITS); results for Malassezia and Candida are not directly comparable with Zou 2022 (ITS) due to platform differences. E2: same protocol across

Ref	Study	Selection				Comp.	Exposure			Total /9	Overall	Compartment, setting, and basis for the scores
		S1	S2	S3	S4	C1	E1	E2	E3			
												all 60 participants, same matrices (saliva, subgingival plaque, faeces). E3 scores 0: non-respondents not described.
51	Qu et al. 2022	**0**	**0**	**1**	**0**	**0**	**1**	**1**	**0**	3	Poor	Gut; Yantai, China (63 hypertensive patients, 34 normotensive spouse controls). Reclassified from cohort to case–control: despite the paper's subtitle, recruitment ran from December 2019 to June 2020 at a single hospital with no follow-up. The controls are the patients' spouses, included to mitigate the influence of shared diet and lifestyle. S1 scores 0: the inclusion criterion as printed reads 'without using antihypertensive drugs, systolic blood pressure <= 140 mmHg and/or diastolic blood pressure <= 90 mmHg' as a criterion for including a hypertensive patient — the inequality is inverted in the source — and no blood pressure measurement protocol is described (no device, no rest period, no number of readings). S2 scores 0: single hospital, with no indication of a consecutive series. S3 scores 1: community controls, spouses rather than inpatients. S4 scores 0: controls are described only as healthy, with no documented blood pressure measurement confirming normotension. C1 scores 0: no multivariable model; one-way ANOVA, Welch's ANOVA, Spearman and Pearson correlations only. The spouse design controls household diet and lifestyle, but spouses differ systematically by sex, so no star is awarded. E1 and E2 score 1: 16S rRNA V3–V4 with 341F/806R primers on Illumina NovaSeq 6000, Uparse v7.0.1001, SILVA v138.1 via Mothur v1.46.1, applied identically to both groups. E3 scores 0: non-response rate not reported. Further limitations: no correction for multiple comparisons (MetaStat and LEfSe with LDA > 4 against a bare P < 0.05 threshold, across phylum, class, order, family and genus); 97% OTU clustering; functional profiling by Tax4Fun, which is inference rather than measurement; and exclusion of diabetes and of digestive, respiratory and neurological disease, giving a highly selected population.
42	Yan et al. 2017	**0**	**0**	**0**	**1**	**1**	**1**	**1**	**0**	4	Poor	Gut; China. Contributes to the Roseburia and Klebsiella convergence counts. — RECLASSIFIED TO CROSS-SECTIONAL (see Table S2c). Scores shown here reflect the case-control NOS items as far as applicable; S4 maps to E1 (ascertainment of exposure); O1 is not applicable since blood pressure is the exposure in case-control terms. See Table S2c for the full assessment.
46	Cheng et al. 2025	**0**	**0**	**0**	**1**	**1**	**1**	**1**	**0**	4	Poor	Gut; Lishui, Zhejiang, China. N=205 (153 hypertensive, 52 controls). Null alpha diversity alongside significant beta-diversity separation (ADONIS P<0.01). — RECLASSIFIED TO CROSS-SECTIONAL (see Table S2c). Scores shown here reflect the case-control NOS items as far as applicable; S4 maps to E1 (ascertainment of exposure); O1 is not applicable since blood pressure is the exposure in case-control terms. See Table S2c for the full assessment.
48	Mushtaq et al. 2019	**0**	**0**	**0**	**0**	**0**	**1**	**1**	**0**	2	Poor	Gut; China. Restricted to grade 3 hypertension; indirectness for the general hypertensive population. — Cases restricted to grade 3 hypertension, which bears on S2 once the source paper is checked.
49	Zuo et al. 2019	**1**	**0**	**0**	**0**	**0**	**1**	**1**	**0**	3	Poor	Gut; China. Contributes to the Faecalibacterium and Roseburia convergence counts. — RECLASSIFIED TO CROSS-SECTIONAL (see Table S2c). Scores shown here reflect the case-control NOS items as far as applicable; S4 maps to E1 (ascertainment of exposure); O1 is not applicable since blood pressure is the exposure in case-control terms. See Table S2c for the full assessment.
50	Silveira-Nunes et al. 2020	**1**	**1**	**1**	**1**	**1**	**1**	**1**	**0**	7	Good	Gut; Brazil. Only replication of the Faecalibacterium and Roseburia signals outside East Asia. — Scored from the full text (Silveira-Nunes 2020, Brazil). S1: cases defined as chronically hypertensive for >10 years on antihypertensive treatment, recruited from Casa Unimed, a community health promotion centre; not a hospital-based series. S2: consecutive series within the

Ref	Study	Selection				Comp.	Exposure			Total /9	Overall	Compartment, setting, and basis for the scores
		S1	S2	S3	S4	C1	E1	E2	E3			
												community centre without additional selection. S3: controls from the same community centre, not hospital controls. S4: controls defined as having no report of hypertension by self-report; not verified by measurement; sufficient for one star but not for a stricter definition. C1: diet assessed by 24-hour recalls over 3 days (2 weekdays + 1 weekend), analysed with the Brazilian TACO food composition table - one of the few studies in the corpus with structured dietary data; however, diet does not enter any multivariable model and LEfSe/Mann-Whitney analyses are unadjusted. E1: 16S rRNA V3-V4 on Illumina MiSeq 2x300 bp, primers 341F/805R, PANDAseq + QIIME, UCLUST 97%, RDP classifier against Greengenes May 2013 release. E2: same protocol in both groups. E3 scores 0: non-respondents not described. NOTE: blood pressure stratification is based on self-reported pressure at the time of diagnosis and antihypertensive medication use, not on a measurement performed for this study. Table S3 should record: BP ascertainment = self-reported at diagnosis, not re-measured. NOTE: Greengenes May 2013 is outdated even in 2020, with non-updated taxonomy. Cytokines (not cited in the main text): IFN-gamma, TNF-alpha, IL-17A, IL-6, IL-10, IL-4, IL-2 from PBMC cultures, analysed by CBA and functional cytokine signature method.

S1 adequacy of the case definition (blood pressure ascertainment method and threshold); S2 representativeness of the cases; S3 selection of controls; S4 definition of controls (documented absence of hypertension); C1 comparability of cases and controls (up to two stars, as above); E1 ascertainment of exposure (sampling, extraction and sequencing protocol); E2 same method of ascertainment for cases and controls; E3 non-response rate.

Table S2c. Cross-sectional studies, Modesti adaptation (maximum 10 stars)

Ref	Study	Selection (max 5)				Comp.	Outcome (max 3)		Total /10	Overall	Compartment, setting, and basis for the scores
		S1	S2	S3	S4	C1	O1	O2			
21	Gao et al. 2025	**0**	**0**	**0**	**2**	**0**	**0**	**1**	3	Unsatisfactory	Gut + mycobiome; multiple cohorts. Retained as a benchmark. The only multi-cohort test of mycobiome reproducibility, and it is negative. — Scored from the full text (Gao 2025, mSystems). RECLASSIFICATION: the paper analyses ORIGINAL data from two cohorts in Beijing and Dalian (N=159 HTN + 101 HC) with shotgun metagenomics performed for this study; it is a primary analysis, not a secondary synthesis, and should be in the primary corpus, not the benchmark list. S1 scores 0: no description of recruitment source, setting or invitation method. S2 scores 0: no sample size calculation. S3 scores 0: non-respondents not described. S4 scores 2: shotgun metagenomics on Illumina platform, species-level taxonomic annotation; detailed pipeline presumably in the Supplementary Material (not provided). C1 scores 0: no multivariable confounding adjustment; Wilcoxon rank-sum, PERMANOVA, MaAsLin2 and random forest used, none adjusting for cardiometabolic confounders. O1 scores 0: blood pressure measurement not described in the available text; phenotype details in Supplementary Table S1 (not provided). O2: combined p-values using Fisher's exact test, FDR Benjamini-Hochberg (q=0.25 for bacteriome, q=0.73 for mycobiome). KEY FINDING FOR §3.3: the gut bacteriome shows consistent alterations across both cohorts (61 species), while the gut mycobiome does not replicate (only Blastomyces emzantsi c231 consistent across cohorts). This is the first cross-cohort study testing both kingdoms simultaneously and directly supports the argument for poor mycobiome replicability in the review.
25	Verhaar et al. 2020	**1**	**1**	**0**	**2**	**1**	**1**	**0**	6	Satisfactory	Gut; HELIUS, Netherlands (N≈4,600+). Ethnicity-stratified; faecal SCFA measured. — Scored from the full text (Verhaar 2020, HELIUS baseline cross-sectional). Same cohort and sequencing protocol as Verhaar 2026: random sampling from Amsterdam municipality registry, stratified by ethnicity

Ref	Study	Selection (max 5)				Comp.	Outcome (max 3)		Total /10	Overall	Compartment, setting, and basis for the scores
		**S1**	**S2**	**S3**	**S4**	**C1**	**O1**	**O2**			
											(Dutch, South-Asian Surinamese, African Surinamese, Ghanaian, Turkish, Moroccan), 16S V4 on Illumina MiSeq 2x250 bp, 515F/806R, bead-beating, PCR in duplicate. S1-S4: as per Verhaar 2026. C1 scores 1, not 2: the main analysis uses gradient boosted tree machine learning in nested cross-validation to predict SBP/DBP from microbiota; the 'corrected' models compute residuals after a linear model for age, sex and BMI only, which is not confounding adjustment in the NOS sense. A full linear regression model (age, sex, BMI, smoking, antihypertensive medication, diabetes) is run only for the 10 best-predicting ASVs in a subgroup of 200 selected participants, not in the full cohort. O1: Microlife WatchBP Home, average of two readings, five minutes supine rest - identical to Verhaar 2026. O2 scores 0: the main machine learning analysis produces importance scores, not P values, with no multiple testing correction; Spearman correlations between the 10 best ASVs and BP are reported without correction; Mann-Whitney U comparisons for SCFAs in the subgroup of 200 are uncorrected. No mention of FDR or Bonferroni anywhere. SCFA PLATFORM: HPLC with UV detection (De Baere method) on a subgroup of 200 Dutch participants aged <=50, selected for extreme SBP (top 100 matched to bottom-35th-percentile 100 by sex, age and BMI) after freeze-drying and dry-weight correction. This is the third distinct SCFA platform in the corpus: Huart 2019 uses NMR, de la Cuesta-Zuluaga uses GC/MS, and Verhaar 2020 uses HPLC-UV. Additionally, the matrix is freeze-dried faeces corrected to dry weight, unlike the other two. Values are not comparable across these three studies for three simultaneous reasons: platform, matrix, and normalisation procedure. The SCFA subgroup is purposively selected for extreme blood pressure, then matched - any SCFA-blood pressure estimate from it is not generalisable to the full cohort and is not comparable with studies measuring SCFAs in unselected samples.
27	Louca et al. 2021	**1**	**1**	**0**	**2**	**1**	**1**	**1**	7	Good	Gut; TwinsUK (N=871) + PREDICT-1 (N=448), UK; female-only. Internal discovery–replication with inverse-variance random-effects meta-analysis conducted by the original authors; treated as a single already-validated evidence unit. — Scored from the full text (Louca 2021, TwinsUK + PREDICT-1). Evaluated as a single discovery+replication unit as stated in the main text of the review; the Modesti scale is applied to the study as a whole. S1: TwinsUK is a national registry of adult twins recruited as volunteers without disease selection; PREDICT-1 recruits from TwinsUK and the general population via online advertising. Not a random sample, but reasonably representative of adult women in the UK. S2: N=871+448 for a binary comparison with FDR correction is amply satisfactory; no power calculation. S3 scores 0: non-respondents not described. S4: 16S V4 on Illumina MiSeq, DADA2 ASVs, QIIME, pipeline and instrument both specified. The authors selected 871 UNRELATED women from TwinsUK, eliminating the family structure by design rather than by model correction; the independence issue does not arise. C1 scores 1, not 2: models adjust for age, age-squared and BMI with FDR correction, but smoking, diet, sodium and antihypertensive medication are absent from the microbiota models - the latter is notable since medication use enters the case definition. O1 scores 1: triplicate measurements with a validated device (Marshall mb02 OR Omron Mx3 OR Omron HEM713C), mean of the second and third readings, participant seated >=3 minutes - a good protocol, but THREE different devices are used without specifying whether they were randomly assigned or used by cohort; uncertainty about systematic device-level variation is enough to withhold the second star. O2: FDR<0.05 in the GAMLSS-BEZI discovery analysis, FDR<0.01 in the likelihood-ratio tests, inverse-variance random-effects meta-analysis for replication. CAVEATS: (1) The hypertension definition is age-stratified, creating an unclassified grey zone (SBP 121-139, DBP 81-89) that is neither case nor control; the number of participants excluded by this rule is not reported. (2) The discovery and replication models use different thresholds: FDR<0.01 for discovery, P<0.05 (not FDR) for replication; this is methodologically acceptable as a discovery-replication approach but should be stated explicitly when citing the two replicated genera as

Ref	Study	Selection (max 5)				Comp.	Outcome (max 3)		Total /10	Overall	Compartment, setting, and basis for the scores
		S1	S2	S3	S4	C1	O1	O2			
											independently validated. (3) PREDICT-1 is a single-arm nutritional intervention study, not an observational study; participants completed test meal challenges and a 13-day home-based phase before measurement, and microbiota composition at sampling may have been influenced by the intervention.
31	Guo et al. 2026	**1**	**1**	**0**	**2**	**0**	**1**	**0**	5	Satisfactory	Oral; Guangzhou, China. Four-group design (healthy controls, periodontitis alone, hypertension alone, comorbidity); the hypertension-versus-controls comparison contributes to the primary oral synthesis, with periodontitis controlled by design. — Scored from the full text (Guo 2026, BMC Oral Health). S1: Department of Dentistry and Department of Cardiology, First Affiliated Hospital of Jinan University, January-September 2024; four predefined groups with explicit structured criteria. S2 scores 1: a priori power calculation with G*Power 3.1, Cohen's d=0.4, alpha=0.05, power=0.80, minimum N=15 per group (N*=60), with 10% attrition budget leading to 67 enrolled. Third study in the corpus with a formal power calculation. S3 scores 0: non-respondents not described. S4: 16S rRNA V3-V4 on Illumina NovaSeq 6000, primers 338F/806R, TGuide S96 kit, QIIME2, Cutadapt v0.33, Trimmomatic, 97% OTU clustering (reference database not stated in the available text). C1 scores 0: no multivariable model; Kruskal-Wallis, Tukey HSD, one-way ANOVA, LEfSe and PERMANOVA used; smoking excluded by design. O1 scores 1: diagnosis confirmed by three independent office measurements on separate occasions (Chinese Guidelines 2018: SBP>=140 and/or DBP>=90); device not specified but the repeated-visit protocol is more rigorous than a single measurement. O2 scores 0: PICRUSt2 functional pathway analysis via STAMP reports P<0.05 without FDR correction; genus-level Kruskal-Wallis is FDR-corrected but pathway analysis is not.
32	Liu et al. 2026	**0**	**0**	**0**	**2**	**2**	**1**	**1**	6	Satisfactory	Gut; Daqing vs Haikou, China (N=60). Within-category geographic comparison between two hypertensive populations. — Scored from the full text (Liu 2026, Daqing vs Haikou). S1 scores 0: two single hospitals (Daqing and Haikou); clinical convenience sample. S2 scores 0: no power calculation; N=70 without justification. S3 scores 0: non-respondents not described. S4: shotgun metagenomics on Illumina NovaSeq 6000 PE150, Fastp v0.23.4, MEGAHIT v1.2.9, MetaGeneMark v3.26, MMseq2, DIAMOND v0.9.14 against NCBI NR database; pipeline and database both specified. C1 scores 2: multivariate linear regression adjusted for age, BMI, smoking and drinking status applied to candidate genera identified by ALDEx2; the most complete adjustment among the Chinese studies in the corpus. O1 scores 1: three separate measurements on non-same days, at rest, 1-minute intervals (Chinese Hypertension Guidelines 2019); device not specified. Multi-visit protocol is more rigorous than single measurement but device and posture not documented. O2: Benjamini-Hochberg FDR applied where appropriate; ALDEx2 for differential abundance (includes internal correction for compositionality). WITHIN-CATEGORY CONFIRMED: all 70 participants are hypertensive; DQ vs HK is a geographic comparison between two hypertensive populations, not hypertensives vs normotensives. METABOLOMICS: LC-MS/MS, Q-Exactive, ACQUITY UPLC T3, positive and negative ionization modes, KEGG + HMDB annotation, mass error <10 ppm. Fourth distinct metabolomics platform in the corpus (after NMR, GC/MS and HPLC-UV); values are not cross-platform comparable.
33	Valdez-Palomares et al. 2025	**1**	**0**	**0**	**2**	**1**	**1**	**1**	6	Satisfactory	Gut; Mexico City, Mexico (N=240). Within-category comparison (controlled vs uncontrolled hypertension, all treated). First Latin American population in the corpus. — Scored from the full text (Valdez-Palomares 2025). S1: consecutive recruitment from two primary care centres in Mexico City, with explicit inclusion criteria. S2 scores 0: no power calculation; N=240 without justification. S3 scores 0: non-respondents not described. S4: 16S V3-V4 on Illumina MiSeq, primers 341F/805R, DADA2 in QIIME2 v2021.8, SILVA 138.1, prevalence filter >10% in at least one group. C1: adjusted for sex, age, BMI, smoking, alcohol and diabetes; antihypertensive drug class, sodium and

Ref	Study	Selection (max 5)				Comp.	Outcome (max 3)		Total /10	Overall	Compartment, setting, and basis for the scores
		S1	S2	S3	S4	C1	O1	O2			
											diet not included. O1: two readings with validated device (Omron HEM-7120), seated after 5 minutes rest, mean of two readings. O2: Benjamini-Hochberg FDR across all main analyses. WITHIN-CATEGORY CONFIRMED: all 240 participants are hypertensive adults on antihypertensive treatment; the comparison is controlled (SBP<130/80 on treatment, ACC/AHA 2017 criteria) vs uncontrolled (SBP>=130/80 on treatment). NOTE: the control threshold is 130/80 (ACC/AHA 2017), more stringent than the 140/90 used by most other studies in the corpus; comparisons across studies on this sub-analysis require caution. Additional biomarkers: hsCRP, TNF-alpha, IL-6, IL-10, lipid profile, glucose.
34	Palmu et al. 2020	**1**	**1**	**0**	**2**	**2**	**1**	**1**	8	Good	Gut; FINRISK, Finland (N=6,953). Association attenuated to non-significance in the fully multivariable-adjusted model. — Scored from the full text (Palmu 2020, FINRISK 2002 cross-sectional baseline). Same cohort as Yeo 2026; shares the sampling frame (stratified random sample from the national register, 25-74 years, 6 regions, 13,437 invited, 65.5% participation) and the shotgun metagenomics protocol (HiSeq 4000, 150 bp paired-end, SHOGUN v1.0.5). S1: population-based random sample as above. S2: N=6,953 amply satisfactory. S3 scores 0: non-respondents not described; exclusions within the analytic sample are detailed but not the comparison of participants with the broader invited population. S4: shotgun metagenomics, Illumina HiSeq 4000, SHOGUN v1.0.5, RefSeq v82 (May 2017). NOTE: Yeo 2026 uses Greengenes2 (2022) on the same cohort; direct taxonomic comparisons between the two papers require caution because of database-level differences. C1 scores 2 and is the most detailed adjustment in the corpus: age, sex, BMI, smoking, exercise, diabetes, and antihypertensive medication broken down by ATC class - diuretics (C03), beta-blockers (C07), calcium-channel blockers (C08), and RAS inhibitors (C09), each entered separately; medication use defined from the national Drug Purchase Register (purchase within 4 months before baseline), not self-reported. O1: two nurse-measured office readings, right arm, mercury sphygmomanometer, 5-min seated rest, mean of the two. O2: Benjamini-Hochberg correction via DESeq2 for genus-level analyses; PERMANOVA with 999 permutations for beta diversity. SODIUM: measured in a subsample of 829 participants by 24-h urine collection (ion-selective electrode, Optima analyser, Thermo Electron); analysed separately for the microbiota-sodium relationship but not included in the main blood pressure model. Incomparable with SCAPIS (Kawasaki formula on spot urine). Source code: 10.5281/zenodo.3622730.
37	Sun et al. 2019	**1**	**0**	**1**	**2**	**2**	**1**	**1**	8	Good	Gut; CARDIA, USA (N=529). Richness inversely associated with prevalent hypertension in the fully adjusted model. — Scored from the full text (Sun 2019, CARDIA). S1: sequential recruitment to a target of 600 from CARDIA Year 30 participants, with quotas by field centre (300 Chicago, 100 each of 3 others); not strictly probabilistic but drawn from a community cohort initiated in 1985-86 with race and sex quota sampling, and the source and restriction are explicitly declared. S2 scores 0: no sample size justification; the target of 600 is pragmatic. S3 scores 1: the analytic sample (N=529) is explicitly compared with the 2,752 CARDIA Year 30 participants who did not join the microbiome study, using chi-squared and non-parametric tests. S4: 16S V3-V4 on Illumina MiSeq 2x300, primers 341F/785R, PEAR merging, RDP Classifier v2.12 at 80% confidence, BioLockJ pipeline. C1: model 3 adjusts for sequencing run, field centre (4 categories), sex, race, age, education, physical activity, smoking, diet quality score, and antihypertensive medication use; the last is explicitly described as a methodological choice because medications may alter the gut microbiota. NOTABLE: models 4 and 5 adjust separately for BMI and waist circumference as POTENTIAL MEDIATORS, not confounders, tested sequentially - the only study in the corpus to distinguish explicitly between confounder and mediator adjustment. O1: three oscillometric readings (Omron HEM907XL, calibrated to random-zero sphygmomanometer), mean of the second and third, seated with elbow and forearm on the chair

Ref	Study	Selection (max 5)				Comp.	Outcome (max 3)		Total /10	Overall	Compartment, setting, and basis for the scores
		S1	S2	S3	S4	C1	O1	O2			
											armrest, four cuff sizes by arm circumference including a thigh cuff for >50 cm. O2: Benjamini-Hochberg FDR for taxonomy, PERMANOVA with 1,000 permutations for beta diversity. NOTE: BP is assessed by office measurement only, not ABPM; the OR 0.75 (95% CI 0.60-0.94) cited in the review is from a logistic model for prevalent hypertension defined as SBP>=140 or DBP>=90 or antihypertensive use, and should be contextualised against Lin 2025 when comparing. Diet assessed by a brief 23-item frequency questionnaire, less precise than the FFQs used in HELIUS and FINRISK. Stabilisation: tubes pre-filled with RNAlater for overnight shipping; no other study in the corpus describes this pre-analytical step.
38	Dan et al. 2019	**0**	**0**	**0**	**1**	**1**	**0**	**1**	3	Unsatisfactory	Gut; China. Propensity-score matched design. — Scored from the full text (Dan 2019). S1 scores 0: participants are teachers and staff at the hospital of Minzu University of China recruited during a workplace health screening - a convenience occupational sample, not a population sample. S2 scores 0: no power calculation; N=129 without justification. S3 scores 0: non-respondents not described. S4 scores 1, not 2: 16S V3-V4 on Illumina HiSeq 2500, USEARCH v9 in QIIME, 97% OTU clustering, RDP Classifier; pipeline and platform specified but the taxonomic reference database is not declared. C1 scores 1: propensity score matching (PSM) is applied, but the variables on which matching was performed are not declared anywhere in the paper; without knowing the matching variables it is impossible to assess whether major cardiometabolic confounders (BMI, smoking, diet, antihypertensive medication) were controlled. One star for the explicit attempt at adjustment. O1 scores 0: the Chinese Guidelines 2010 threshold is cited as the inclusion criterion, but there is no description of how blood pressure was actually measured - no device, no number of readings, no rest period. The normotensive inclusion criterion uses non-strict inequalities (90 mmHg <= SBP <= 140 mmHg), which overlaps with the hypertensive threshold - likely a typographic error in the source. O2: DESeq2 with q<0.05 for differential abundance; P<0.05 for other analyses; hybrid but correction applied for the primary analysis. NOTE: patients receiving antihypertensive therapy are explicitly excluded, which removes medication confounding but means hypertension prevalence is underrepresented relative to the true hypertensive population. ANTIBIOTICS: excluded within 8 weeks - the longest exclusion window among studies using time-based exclusion in this corpus.
39	Lv et al. 2023	**0**	**0**	**0**	**2**	**1**	**2**	**0**	5	Satisfactory	Gut; north-western China. — Scored from the full text (Lv 2023, Xi'an, Northwestern China). S1 scores 0: outpatient clinics of a single hospital (Honghui Hospital, Xi'an Jiaotong University); clinical convenience sample. S2 scores 0: no power calculation; N=205 without formal justification. S3 scores 0: non-respondents not described. S4 scores 2: both 16S rRNA V3-V4 (NovaSeq 6000, primers 338F/806R, UPARSE v7, QIIME v1.7, 97% OTU, RDP classifier) and shotgun metagenomics (NovaSeq 6000, PE250, SOAP2/MetaGeneMark, KEGG 73.1) applied in parallel; the only study in the corpus to use both methods simultaneously. C1 scores 1: antihypertensive medication is excluded by design (explicit exclusion criterion), removing the primary pharmacological confounder at source rather than in the model. However, the main statistical models are Wilcoxon rank-sum and LEfSe with no adjustment for BMI, smoking or diet. Note: excluding antihypertensive users introduces selection bias in the opposite direction to Kim 2018 (uncontrolled treated hypertensives) - these are untreated hypertensives, who may not represent the real hypertensive population. O1 scores 2: Omron HEM-7130, left arm at heart level, seated with palm face up, 3-5 min rest, two readings at 1-2 min interval, mean of two. Complete and documented protocol. O2 scores 0: LEfSe with LDA>2 and P<0.05, no FDR correction; Wilcoxon rank-sum uncorrected. THRESHOLD: SBP>=130 and/or DBP>=80 (ACC/AHA 2017); controls must have SBP<120 and DBP<80, excluding the 120-129/80-89 elevated BP zone. Third distinct threshold in the corpus after 140/90 and ABPM thresholds.

Ref	Study	Selection (max 5)				Comp.	Outcome (max 3)		Total /10	Overall	Compartment, setting, and basis for the scores
		S1	S2	S3	S4	C1	O1	O2			
41	Song et al. 2023	**0**	**0**	**0**	**2**	**1**	**0**	**1**	4	Unsatisfactory	Gut; South Korea. Association conditional on enterotype; bears on the poor cross-study replication of single-taxon comparisons. — Scored from the full text (Song 2023, South Korea). S1 scores 0: volunteer convenience sample recruited through announcements at Ulsan University Hospital; not a population sample. S2 scores 0: no power calculation; N=393 without justification. S3 scores 0: non-respondents not described. S4: 16S V3-V4, Illumina MiSeq, primers 341F/806R, QIIME2 v2022.8, SILVA 138, DADA2 ASVs, rarefied to 8,000 reads. C1: adjusted for age, sex and BMI in the enterotype-hypertension association analysis; smoking, sodium, antihypertensive medication and diet not included. O1 scores 0: blood pressure measured by trained nurses but no device, number of readings or rest period is documented. O2: Benjamini-Hochberg FDR across all main analyses. UNIQUE FEATURE: the only study in the corpus to stratify the microbiota-hypertension analysis by enterotype (Dirichlet Multinomial Mixture, k=2: Prevotella-dominant enterotype 1 and Bacteroides-dominant enterotype 2) before testing for association; the main finding is that the association exists only in some enterotypes and not others, which offers a partial explanation for the poor cross-study replicability of individual taxa. NOTE: the choice of k=2 for enterotype classification strongly influences results; no sensitivity analysis on k is reported. NOTE: antihypertensive medication use is neither an exclusion criterion nor a covariate, which is a substantive gap in a study aiming to isolate the microbiota-blood pressure relationship.
43	Joishy et al. 2022	**1**	**0**	**0**	**2**	**0**	**0**	**0**	3	Unsatisfactory	Gut; Assam, India. Two agropastoralist communities; the only South Asian population in the corpus. — Scored from the full text (Joishy 2022, Assam, India). S1: three distinct geographic communities in Assam, India (Aanthmile, Jagiroad, Kamalabari/Majuli), agropastoral populations. S2 scores 0: no power calculation; N=71 without justification. S3 scores 0: non-respondents not described. S4: 16S rRNA V3-V4 on Illumina MiSeq 2x300 bp, primers 341F/805R, sequenced at Macrogen (Seoul), DADA2 ASVs, SILVA 138 training set, phyloseq pipeline, filter <5% of individuals. RNAlater stabilisation during collection, same as CARDIA (Sun 2019). C1 scores 0: no multivariable model; Kruskal-Wallis, PERMANOVA and DESeq2 without confounding adjustment. O1 scores 0: blood pressure is measured on-site but no measurement protocol is described - no device, no number of readings, no rest period, no formal diagnostic threshold. O2 scores 0: DESeq2 used without explicit FDR correction in the available Methods; Kruskal-Wallis uncorrected. UNIQUE FEATURE: the only study in the corpus with a South Asian population; three agropastoral communities in Assam. Results that replicate here have stronger generalisability; results that do not replicate may reflect genuine population specificity.
44	Huart et al. 2019	**0**	**0**	**0**	**2**	**0**	**2**	**1**	5	Satisfactory	Gut; Belgium; male-only. Blood pressure by 24-hour ambulatory monitoring. Faecal SCFA quantified. — Scored from the full text (Huart 2019). INCOMPLETE: the Statistical Analysis section was not available, so C1 and O2 remain open; the total will fall between 4 and 7. S1 scores 0: single-centre recruitment at the University of Liege, described only as adult male individuals with or without known primary hypertension and normal kidney and liver function, with no recruitment source or sampling frame given. S2 scores 0: no sample size justification. S3 scores 0: non-respondents not described. S4 scores 2: 16S rRNA V1-V3 on Illumina MiSeq v3, MOTHUR v1.35, UCHIME, 0.03 clustering distance, SILVA v1.28, with a 20-species positive control and a negative control included in the run. O1 scores 2 and is the first full mark on this item in the corpus: 24-hour ABPM with a Spacelabs 90207 device following the European Society of Hypertension protocol, readings every 20 minutes by day and 30 minutes at night, daytime and nighttime means, and dipper/non-dipper classification. THRESHOLD: hypertension defined as 24-hour mean >=130/80 mmHg by ESH criteria, or antihypertensive use at any blood pressure level, with a third borderline group defined by isolated daytime (>=135/85) or nocturnal (>=120/70) hypertension. This is the sharpest threshold

Ref	Study	Selection (max 5)				Comp.	Outcome (max 3)		Total /10	Overall	Compartment, setting, and basis for the scores
		S1	S2	S3	S4	C1	O1	O2			
											divergence in the corpus, and structural rather than numeric, since an ambulatory threshold and an office threshold are not the same quantity. SCFA PLATFORM: 1H-NMR on a Bruker Avance at 500.13 MHz with a 5-mm TCI cryoprobe, maleic acid internal standard, CPMG for stool supernatants and serum and NOESY-presat for urine, in THREE matrices - faeces, serum and urine. De la Cuesta-Zuluaga uses GC/MS, so the two are not convertible; this is the methodological citation supporting the decision not to pool. ANTIBIOTICS: excluded only at the time of enrolment, with no time window - against 1 month in FINRISK, 3 months in HELIUS, and adjustment without exclusion in GGMP. MINOR: the ethics statement calls this a single-centre prospective study, but the analysis is a cross-sectional comparison of three ABPM-defined groups; the cross-sectional classification stands. Data availability: raw reads under NCBI BioProject PRJNA507937, metabolomics on MetaboLights. C1 scores 0: no multivariable model adjusting for cardiometabolic confounders is performed at any point; Kruskal-Wallis with Bonferroni, ANOVA, Spearman correlations and PLS-DA are used, but none controls for age, BMI, smoking or diet in the microbiota-blood pressure relationship. O2 scores 1: correction for multiple comparisons is applied and named - Bonferroni for the baseline characteristics table, Benjamini-Hochberg-Yekutieli for diversity and correlation analyses, and Tukey-Kramer for group comparisons. SCFA univariate: 1H-NMR integration at 1.93, 1.56 and 1.05 ppm for acetate, butyrate and propionate respectively, maleic acid as internal standard, Topspin v3.2. SCFA multivariate: AMIX v3.9.14, 0.04 ppm bucketing, 0.5-10.00 ppm, Pareto scaling, PLS-DA in SIMCA v13.0.3. TOTAL: 5/10, Satisfactory.
45	de la Cuesta-Zuluaga et al. 2018	**1**	**0**	**0**	**2**	**0**	**0**	**1**	4	Unsatisfactory	Gut; Colombia. Hypertension as one of several cardiometabolic outcomes. Faecal SCFA quantified. — Scored from the full text (de la Cuesta-Zuluaga 2018, Colombian cohort). S1: five Colombian cities, quota sampling by city, sex, age range and BMI, with random sampling from two health-service institutions per city; not strictly probabilistic but the sampling structure is explicit and reasonably representative of urban adult Colombians. S2 scores 0: no power calculation; N=441 is a pragmatic target. S3 scores 0: non-respondents not described. S4: 16S V4, Illumina MiSeq v2, primers F515/R806, Mothur v1.36, SILVA v123, UCHIME chimera detection, 97% OTU clustering, Greengenes 13_8_99 taxonomy. Negative controls, extraction blanks, mock community HM-782D, 5 inter-run replicates, MCC=0.79 - high-quality microbiological protocol. C1 scores 0: hypertension is one of several cardiometabolic outcomes used to characterise co-abundance groups (CAGs), not a primary endpoint with a dedicated multivariable model; the analyses are Spearman correlations, ANOVA on CAG subgroups, and Random Forest classification of microbial configurations, none of which adjusts for cardiometabolic confounders in the relationship between microbiota and blood pressure. O1 scores 0: blood pressure measured with a Rossmax AF701f digital device, two measurements, mean reported; no rest period, no position, no validated protocol or guideline reference documented. O2: FDR correction applied and named across all main analyses (Spearman, PERMANOVA, ANOVA, Kruskal-Wallis). Hypertension is not the primary outcome in this study: it is a descriptive covariate characterising microbial co-abundance groups (CAGs) in a general-population cohort, not an outcome analysed in a dedicated multivariable model. Accordingly, this study is discussed in Section 3.2.8 as a dedicated sub-analysis rather than pooled with the primary normotensive-vs-hypertensive synthesis. SCFA: not directly measured; functional potential inferred with Tax4Fun on SILVA 123; this is inference, not measurement, and should be marked accordingly in Table S3. Antibiotics: excluded within 3 months, plus antiparasitics.
47	Liu et al. 2025	**1**	**1**	**0**	**2**	**2**	**0**	**1**	7	Good	Gut; Guangdong Gut Microbiome Project, China (N=6,999). RECLASSIFIED: the paper's title says multi-regional prospective cohort, but its Methods open by describing the GGMP as a population-based cross-sectional study, with no follow-up, no incidence and no time-to-event. Assessed here as

Ref	Study	Selection (max 5)				Comp.	Outcome (max 3)		Total /10	Overall	Compartment, setting, and basis for the scores
		S1	S2	S3	S4	C1	O1	O2			
											cross-sectional. Co-abundance network design, reported narratively. — Scored from the full text (Liu 2025, Guangdong Gut Microbiome Project). RECLASSIFIED from cohort to cross-sectional: the Methods open by describing the GGMP as a population-based cross-sectional study carried out in 2015-2016, with no follow-up, no incidence and no time-to-event analysis, contrary to the paper's own title. S1: population-based across 14 regions of Guangdong Province, N=6,999. S2: no power calculation, but the sample size is amply satisfactory. S3 scores 0: neither the response rate nor the characteristics of non-respondents are described. S4: 16S rRNA V4 on Illumina HiSeq 2500, MiBioGen consortium pipeline, Fastp v0.23.2, subsampling to 10,000 reads, RDP Classifier v2.12 against SILVA v128 - pipeline and reference database both specified. C1: eight confounders - age, sex, BMI, smoking, antibiotic use, diabetes, hypercholesterolaemia and obesity. O1 scores 0: participants are stratified into hypertension grades on SBP and DBP, so blood pressure was measured, but neither the measurement protocol nor the diagnostic threshold appears anywhere in the Methods; they are presumably in the GGMP cohort description (their ref 32), which under our rule is not consulted. TO CHECK: if the paper's body describes a single office measurement this rises to 1 (total 8), if repeated measurements to 2 (total 9); it is the only item that can still move the result. O2: Wilcoxon rank-sum and Kruskal-Wallis, Benjamini-Hochberg FDR, CCLasso with 20 iterations and 100 bootstrap resamples, random-effects meta-analysis with BH. CAVEATS: antibiotic use is adjusted for as a covariate rather than used as an exclusion criterion, unlike HELIUS (excluded within 3 months) and FINRISK (within 1 month) - a substantive methodological difference between the three largest studies in the corpus, since adjusting for exposure in a linear model is not equivalent to removing exposed participants; the Methods refer in one place to four hypertension grades and in another to stratification into three; BMI and obesity both appear in the adjustment set and largely encode the same information.
57	Lin et al. 2025	**1**	**1**	**0**	**2**	**2**	**2**	**1**	9	Very good	Gut; SCAPIS, Sweden. 24-hour blood pressure measurements; one of only two studies in the corpus with ambulatory blood pressure data. — Scored from the full text (Lin 2025, SCAPIS). S1: random sample from the population register of individuals aged 50-64 from six counties in Sweden (N=30,154 invited). S2: sample size amply satisfactory (N=3,695 ABPM subsample, N=2,770 office subsample). S3 scores 0: the characteristics of non-respondents are not described; it is known that 30,154 were invited but how participants compare to non-participants is not reported here. S4: shotgun metagenomic sequencing on Illumina NovaSeq 6000, mean 25-26 million read pairs, CHAMP pipeline with HMR05 gene catalogue, GTDB release 214, filter >0.01% in >30% of participants, CLR with universal pseudo-value; negative and positive controls in every extraction round. C1: age, sex, country of birth, smoking, energy-adjusted fibre intake, total energy intake, estimated 24-h sodium excretion (from spot urine morning sample via Kawasaki formula, log-transformed), antidiabetic and antihypidaemic medication, BMI; covariates selected using d-separation on a DAG in DAGitty v3.0. SCAPIS is the only study in the corpus to adjust for sodium, the most physiologically direct confounder in the microbiota-blood pressure relationship. O1 scores 2: 24-h ABPM with Labtech EC-3H/ABP, every 30 min during the day and 90 min at night in Uppsala, every 30 min in Malmo; explicit quality control, with 312 individuals excluded for invalid data. A parallel analysis in the non-ABPM subsample uses an office device (Omron M10-IT, 5 min supine) with the same adjustment model, providing a within-paper estimate of the effect of switching from office to ambulatory measurement - useful for the review's blood pressure axis. O2: Benjamini-Hochberg FDR 5% applied to all linear regression models. NOTE: blood pressure is analysed as a continuous variable (mean and SD of 24-h SBP and DBP), not dichotomised to a hypertension threshold. ANTIBIOTICS: excluded in the 6 months before visit 1 based on the national prescription register - objective, not self-reported, and the longest window in the corpus. PPI: identified by

Ref	Study	Selection (max 5)				Comp.	Outcome (max 3)		Total /10	Overall	Compartment, setting, and basis for the scores
		S1	S2	S3	S4	C1	O1	O2			
											measured plasma omeprazole/pantoprazole from metabolomics, included in a sensitivity analysis. Data: ENA accession PRJEB51353.

S1 representativeness of the sample (1 star); S2 sample size justified and satisfactory (1 star); S3 non-respondents described and comparable (1 star); S4 ascertainment of the exposure, that is, of microbiota composition (up to 2 stars); C1 comparability of the outcome groups, confounders controlled (up to 2 stars); O1 assessment of the outcome, that is, of blood pressure (up to 2 stars: two for a validated objective measurement, one for a record-based or self-reported definition); O2 statistical test appropriate and clearly described, including correction for multiple comparisons (1 star).

Table S2d. Designs outside the scope of the Newcastle–Ottawa Scale

Ref	Study or component	Design	Instrument and domains applied	Judgement and notes
5	Li et al. 2023	Two-sample bidirectional Mendelian randomisation	MR-specific appraisal (not NOS): instrument strength (F-statistic), instrument–exposure relevance, independence, exclusion restriction, MR-Egger intercept for directional pleiotropy, Cochran's Q for heterogeneity, leave-one-out and weighted-median sensitivity analyses, sample overlap.	High risk of bias. Instruments for microbiota composition are weak and taxonomically coarse, as is general for microbiome GWAS data.
40	Czesnikiewicz-Guzik et al. 2019	Mendelian randomisation + randomised controlled trial of non-surgical periodontal therapy	MR arm: 4 SNPs as IVs (LOC107984137, MTND1P5, DEFA1A3, SIGLEC5); palindromic rs1537415 excluded; MR-PRESSO, IVW and weighted median; GWAS BP ~750,000 UK Biobank+ICBP. MR risk of bias: moderate-high (only 4 instruments, F-statistic not declared, sample overlap not evaluated). RCT arm (Cochrane RoB 2): D1 low (computer-generated randomisation, opaque envelope); D2 low (any antihypertensive change = exit); D3 low (ITT primary, per-protocol reported); D4 low (24-h ABPM Spacelabs Ultralite 90217, all cardiovascular assessors masked); D5 low (registered NCT02131922, primary outcome prespecified). RCT overall: low risk of bias.	MR arm: moderate to high risk of bias (four instruments, F-statistic not reported, sample overlap not evaluated). RCT arm: low risk of bias for the primary outcome, with some concerns arising from single blinding. The RCT is the strongest evidence in the corpus: ambulatory blood pressure as the primary outcome, 7 mmHg systolic reduction at two months with intensive periodontal therapy versus conventional care. MR and RCT convergence supports a causal periodontitis–hypertension relationship despite the limitations of the MR arm.
4	Li et al. 2017 — nested animal component	Faecal microbiota transplantation from human donors into germ-free mice	SYRCLE risk-of-bias tool for animal studies (sequence generation, baseline characteristics, allocation concealment, random housing, blinding of caregivers, random outcome assessment, blinding of outcome assessor, incomplete outcome data, selective outcome reporting).	Unclear risk of bias across most SYRCLE domains: sequence generation, allocation concealment, random housing, and blinding of caregivers and outcome assessors are not reported. Assessed separately from the human case–control component of the same report; contributes to the causal-evidence synthesis only.
18	Chen et al. 2023 — nested animal component	Salivary gavage from hypertensive participants into angiotensin II-infused mice	SYRCLE risk-of-bias tool for animal studies, as above.	Unclear risk of bias across most SYRCLE domains, with randomisation, allocation concealment and blinding not reported; a sample size calculation is reported. Assessed separately from the

Ref	Study or component	Design	Instrument and domains applied	Judgement and notes
				human observational component of the same report.

The four secondary syntheses retained as narrative benchmarks [22,23,24,60] are not part of the corpus and were therefore not assessed with the Newcastle–Ottawa Scale. If the journal requires an appraisal of the benchmark literature, AMSTAR-2 is the appropriate instrument and can be added here.

Table S3. Methodological characteristics of the included studies

This table records, for every included study, the two methodological axes on which the corpus is most heterogeneous and which are treated in this review as discriminant rather than incidental: the method by which blood pressure was ascertained, and the platform on which microbiota and metabolite data were generated. Ascertainment of blood pressure ranges from 24-hour ambulatory monitoring to self-report, and estimates derived from these are not equivalent in precision or in the phenotype they capture; likewise, short-chain fatty acid concentrations measured by gas chromatography–mass spectrometry and by nuclear magnetic resonance are not directly comparable and are not pooled. Em dashes mark fields to be completed from the extraction sheet.

Ref	Study	Country / cohort	Design	Blood pressure ascertainment	Hypertension definition and threshold	Compartment and sample	Platform / region	Multiplicity correction	Covariates in the primary model
16	LaMonte et al. 2022	USA; WHI/OsteoPerio	Prospective cohort	Seated, after 5 min rest; two measurements at least 2 min apart, averaged; Korotkoff phases I and V. Antihypertensive use verified by inspection of pill bottles	Baseline categories aligned with the 2017 ACC/AHA guideline: normotensive SBP <120 and DBP <80 with no medication; undiagnosed elevated SBP >=120 or DBP >=80 with no medication; prevalent hypertension by physician diagnosis with medication. Incident outcome: newly physician-diagnosed hypertension treated with medication	Subgingival plaque	16S rRNA	Benjamini–Hochberg	—
17	Barbadoro et al. 2021	Italy; Ancona	Case–control	Blood pressure checked in controls at physical examination; no protocol, device or number of readings reported	Hypertension status as previously diagnosed by a physician; no numeric threshold reported	Saliva; supra-/subgingival and denture plaque	Targeted qPCR (8 species + total load)	—	BMI (multivariable logistic regression)
18	Chen et al. 2023 (J Adv Res)	China; Shanghai	Case–control + animal experiment	Three readings at 5-min intervals by a physician using an Omron electronic sphygmomanometer; average recorded	SBP >=140 and/or DBP >=90 mmHg	Saliva; subgingival plaque; faeces	—	—	—
31	Guo et al. 2026	China; Guangzhou, Jinan University (N=67: 16 HC, 18 P, 17 T, 16 TP)	Cross-sectional, 4-group (HC/P/T/TP)	3 independent office measurements on separate occasions; device not specified	SBP >=140 and/or DBP >=90 on 3 separate occasions (Chinese Guidelines 2018), or treated with current BP<140/90	Saliva (sublingual mucosa)	16S V3-V4, NovaSeq 6000, QIIME2, 97% OTU	Kruskal-Wallis FDR for taxa; STAMP P<0.05 (uncorrected) for functional pathways	None
4	Li et al. 2017	China	Case–control + FMT	Three readings at 5-min intervals with a random-	Optimal, pre-hypertension and	Faeces	—	—	—

Ref	Study	Country / cohort	Design	Blood pressure ascertainment	Hypertension definition and threshold	Compartment and sample	Platform / region	Multiplicity correction	Covariates in the primary model
				zero mercury column sphygmomanometer, taken seated by nurses or physicians; average recorded	hypertension assigned by reference to national guidelines; no numeric cutoff stated in the report. An independent validation sub-analysis used SBP >=150 or DBP >=100 for cases and SBP <=125 or DBP <=80 for controls				
5	Li et al. 2023	— (GWAS consortia)	Mendelian randomisation	Not applicable (genetic instruments)	Not applicable: genetic instruments for microbiota composition against genetically predicted blood pressure; no participant-level threshold	Faeces (source GWAS)	Not applicable	—	Not applicable
8	Kim et al. 2018	USA	Case–control	—	Not stated numerically in the available text; the hypertensive group had mean blood pressure 160/100 mmHg while on antihypertensive medication	Faeces	—	—	—
19	Zou et al. 2022	China	Case–control (3 groups)	Seated; three readings at 5-min intervals with a random-zero mercury column sphygmomanometer, averaged	SBP >=140 or DBP >=90 and/or use of antihypertensive medication; pre-hypertension SBP 120-139 or DBP 80-89 without medication; normotension SBP <=120 or DBP <=80	Faeces (mycobiome)	—	—	—
20	Chen et al. 2023 (Microbiol Spectr)	China; Shanghai	Case–control	Seated; three readings at 5-min intervals with an Omron electronic sphygmomanometer, averaged	SBP >=140 and/or DBP >=90 mmHg (2018 ESC/ESH guidelines)	Saliva; subgingival plaque; faeces (mycobiome)	Shotgun metagenomics	—	—
21	Gao et al. 2025	China; Beijing + Dalian (N=159+101)	Cross-sectional, 2 cohorts	Not described in available text (see Supplementary Table S1)	Not stated in available text	Faeces (bacteriome + mycobiome)	Shotgun metagenomics, Illumina; species-level annotation	Fisher combined p-values; Benjamini-Hochberg FDR (q=0.25	None

Ref	Study	Country / cohort	Design	Blood pressure ascertainment	Hypertension definition and threshold	Compartment and sample	Platform / region	Multiplicity correction	Covariates in the primary model
								bacteriome, q=0.73 mycobiome)	
25	Verhaar et al. 2020	Netherlands; HELIUS	Cross-sectional	Semi-automatic oscillometric device (Microlife WatchBP Home)	SBP >140 or DBP >90 mmHg, or self-reported use of blood-pressure-lowering medication	Faeces; faecal SCFA	—	—	—
27	Louca et al. 2021	UK; TwinsUK + PREDICT-1	Cross-sectional + replication	Triplicate measurements approximately 1 min apart at heart level; mean of the second and third recorded	Cases: SBP >=140 or DBP >=90, or current antihypertensive use, in participants under 60 years. Controls: age-stratified, SBP <=120 and DBP <=80, or SBP <=115 and DBP <=80 above 50 years, and free of blood-pressure-lowering medication	Faeces	—	FDR < 0.05 (discovery); author-conducted meta-analysis	—
32	Liu et al. 2026	China; Daqing vs Haikou	Cross-sectional (geographic)	Three separate measurements at rest	SBP >140 and/or DBP >90 mmHg on three separate resting measurements (2019 Chinese guidelines). Both compared groups are hypertensive; the contrast is geographic	Faeces; faecal metabolome	—	—	Age, BMI, smoking, alcohol
33	Valdez-Palomares et al. 2025	Mexico; Mexico City	Cross-sectional	—	SBP >=130 or DBP >=80 mmHg (2017 ACC/AHA), applied within a treated population to separate controlled from uncontrolled hypertension	Faeces	—	—	—
34	Palmu et al. 2020	Finland; FINRISK	Cross-sectional	Nurse-measured, sitting, right arm, mercury manometer with a 14x40 cm cuff, after a 5-minute rest; two readings, mean of the two used	Systolic BP >=140 mmHg, diastolic BP >=90 mmHg, or use of antihypertensive medication; blood pressure also analysed as a continuous variable, which is the primary analysis	Faeces	—	—	Age and sex; separate fully multivariable model

Ref	Study	Country / cohort	Design	Blood pressure ascertainment	Hypertension definition and threshold	Compartment and sample	Platform / region	Multiplicity correction	Covariates in the primary model
35	Yeo et al. 2026	Finland; FINRISK	Prospective cohort (20 y)	Nurse-measured; three readings after 5 min seated rest with a standard mercury sphygmomanometer, 1 min apart; mean of the three	Prevalent hypertension: SBP >=140, DBP >=90, self-reported antihypertensive use, or a register-based diagnosis. Incident hypertension: special reimbursement code, or at least three purchases of dihydropyridine calcium-channel blockers or thiazide diuretics in the national Drug Reimbursement Register	Faeces	—	—	Age and sex; separate fully multivariable model
36	Verhaar et al. 2026	Netherlands; HELIUS	Prospective cohort	Office blood pressure after 5 minutes of supine rest; average of two readings with a validated semi-automatic oscillometric device (Microlife WatchBP Home)	Systolic BP >=140 mmHg or diastolic BP >=90 mmHg, or use of antihypertensive medication	Faeces	—	Benjamini–Hochberg	Age, sex, BMI, smoking, alcohol
37	Sun et al. 2019	USA; CARDIA	Cross-sectional	Mean of the second and third of three measurements with an OMRON HEM907XL automated oscillometric monitor calibrated to a random-zero sphygmomanometer; cuff size by arm circumference	SBP >=140 and/or DBP >=90 mmHg and/or current use of antihypertensive medication	Faeces	—	—	Model 3: sequencing run, field centre, sex, race, age, education, physical activity, smoking, diet quality; antihypertensive medication use added for the systolic blood pressure analysis. Model 4 adds BMI as a potential mediator.
38	Dan et al. 2019	China	Cross-sectional (propensity-score matched)	Not described	SBP >=140 or DBP >=90 mmHg in the absence of antihypertensive medication (2010 Chinese guidelines); normotension defined as SBP 90-140 and DBP 60-90	Faeces	—	—	Propensity-score matching on diet, age, sex, BMI and waist-to-hip ratio (no regression adjustment)

Ref	Study	Country / cohort	Design	Blood pressure ascertainment	Hypertension definition and threshold	Compartment and sample	Platform / region	Multiplicity correction	Covariates in the primary model
39	Lv et al. 2023	China; north-west	Cross-sectional	OMRON HEM-7130 electronic sphygmomanometer; two readings 1-2 min apart, averaged	SBP >=130 and/or DBP >=80 mmHg — the 2017 ACC/AHA threshold, lower than that used by most of the corpus	Faeces	—	—	—
41	Song et al. 2023	South Korea; Ulsan (N=393)	Cross-sectional, enterotype-stratified	Nurse-measured; no device, readings or rest documented	SBP >=130 or DBP >=80 or antihypertensive use	Faeces	16S V3-V4, MiSeq, QIIME2 v2022.8, DADA2, SILVA 138	Benjamini-Hochberg FDR	Age, sex, BMI
42	Yan et al. 2017	China	Case–control	Not described	SBP/DBP >=140/90 mmHg; controls required to have current blood pressure <=120/80 mmHg	Faeces	—	—	—
43	Joishy et al. 2022	India; Assam, 3 communities (N=71)	Cross-sectional	Measured on-site; no protocol described	Not formally stated	Faeces	16S V3-V4, MiSeq 2x300 bp, DADA2, SILVA 138, phyloseq	Kruskal-Wallis uncorrected; DESeq2 (FDR not explicit)	Linear mixed models with 15 fixed effects, including generic medicinal use, location, age, sex, BMI and blood parameters
44	Huart et al. 2019	Belgium; Liege, male-only	Cross-sectional	24-h ABPM, Spacelabs 90207, ESH protocol, q20min day / q30min night	24-h mean >=130/80 (ESH) or antihypertensive use	Faeces, serum, urine; SCFA	16S V1-V3, MiSeq v3, MOTHUR 1.35, SILVA 1.28; SCFA by 1H-NMR (Bruker Avance 500 MHz)	—	—
45	de la Cuesta-Zuluaga et al. 2018	Colombia; 5 cities (N=441)	Cross-sectional (CAG/cluster analysis)	Rossmax AF701f digital, 2 readings, mean; no rest/position protocol documented	Hypertension is a descriptive covariate, not a primary endpoint	Faeces (no direct SCFA measurement; functional inference by Tax4Fun only)	16S V4, MiSeq v2, Mothur v1.36, SILVA 123, Greengenes 13_8_99; mock community MCC=0.79	FDR (qvalue package) across all main analyses	None (no multivariable model for BP)
46	Cheng et al. 2025	China; Lishui, Zhejiang	Case–control	Trained nurses; three readings at 5-min intervals with a random-zero mercury column sphygmomanometer, averaged	SBP >=140 or DBP >=90 mmHg, confirmed on repeated examination, or use of antihypertensive medication	Faeces	—	—	—
47	Liu et al. 2025	China; Guangdong GMP	Cross-sectional, population-based	Not described	SBP >=140 or DBP >=90 mmHg, with severity graded 0 to 3;	Faeces	—	—	—

Ref	Study	Country / cohort	Design	Blood pressure ascertainment	Hypertension definition and threshold	Compartment and sample	Platform / region	Multiplicity correction	Covariates in the primary model
					grade 3 defined as SBP >=180 or DBP >=110				
48	Mushtaq et al. 2019	China	Case–control	Not described	Grade 3 hypertension	Faeces	—	—	—
49	Zuo et al. 2019	China	Case–control	Not described	SBP >140 and DBP >90 mmHg	Faeces	—	—	—
50	Silveira-Nunes et al. 2020	Brazil	Case–control	Blood pressure taken at the time of diagnosis; no protocol reported	SBP >140 and DBP >90 mmHg (Brazilian Society of Cardiology / Hypertension / Nephrology, 2010), recorded at diagnosis; all cases subsequently treated	Faeces	—	—	—
51	Qu et al. 2022	China; Yantai	Case–control	—	Chinese Guidelines 2018 (threshold as printed is inverted)	Faeces	16S V3-V4, NovaSeq6000	None applied	None
57	Lin et al. 2025	Sweden; SCAPIS, Uppsala+Malmo (N=3,695 ABPM / 2,770 office)	Cross-sectional	24-h ABPM Labtech EC-3H/ABP q30/q90 min; office Omron M10-IT	Continuous (mean and SD of 24-h SBP and DBP) — no dichotomisation	Faeces; blood for metabolomics	Shotgun, NovaSeq 6000, CHAMP/HMR05, GTDB r214, CLR	Benjamini-Hochberg FDR 5%	Age, sex, country of birth, smoking, fibre, energy, sodium (Kawasaki), antidiabetics, antilipidaemics, BMI; DAG-based selection

Table S4. Handling of antihypertensive medication across the included studies

This table records, for every included study, the proportion of participants receiving antihypertensive therapy, how that information was ascertained, and how the treatment was handled in the analysis. Studies are assigned to one of six mutually exclusive categories. The distinction matters because the categories define different comparisons: a study restricted to treatment-naive patients contrasts untreated hypertension with normotension, whereas a study in which the entire hypertensive group is on long-term therapy contrasts pharmacologically controlled hypertension with the absence of hypertension.

Ref	Study	Participants on antihypertensive therapy	Ascertainment of medication use	Handling in the analysis	Category
4	Li et al. 2017	0%	Recruitment restricted to newly diagnosed patients	Treated patients excluded by design: "all the subjects in the hypertension group were newly diagnosed hypertensive patients prior to antihypertensive treatment". Statins, aspirin, insulin, metformin, nifedipine and metoprolol also absent	1. Treatment-naive by design
5	Li et al. 2023	Not applicable	Not applicable	Mendelian randomisation using genetic instruments; no participant-level medication data	Not applicable
8	Kim et al. 2018	100% of the 18 hypertensive participants	Reported	Entire hypertensive group on therapy with mean blood pressure 160/100 mmHg, i.e. uncontrolled treated hypertension rather than untreated hypertension. Not adjusted	5. Entire hypertensive group treated
16	LaMonte et al. 2022	0% in the prospective analysis; 480/1,215 (39.5%) at baseline in the full sample	Inspection of pill bottles as part of a medication inventory, plus questionnaire	Prospective analysis restricted to the 735 women without prevalent treated hypertension at baseline; medication use forms part of the incident outcome definition. Baseline categories are themselves defined by medication status	1. Treatment-naive by design
17	Barbadoro et al. 2021	Not reported	Assisted interview; hypertensive subjects asked about antihypertensive treatment	Participants with a change of antihypertensive therapy in the month before enrolment excluded. Treatment status collected but neither reported nor entered in the model (only BMI adjusted)	6. Not reported
18	Chen et al. 2023 (J Adv Res)	83.3% (30/36) and 81.4% (48/59) in the two hypertensive groups; 0% in both normotensive groups	Self-reported, tabulated in Table 1	Tested formally as a factor in Adonis/PERMANOVA, with no significant impact on microbiota composition; not entered as a covariate in differential-abundance models	4. Mixed, not adjusted
19	Zou et al. 2022	65.2% (30/46) in the hypertensive group; 0% in pre-hypertensive and normotensive groups by definition	Self-reported, tabulated in Table 1, with duration of hypertension	Formal confounder check reported in the main text: mycobiome of medication users versus non-users did not differ (PERMANOVA R^2 = 0.023, FDR = 0.414). Not entered as a covariate	4. Mixed, not adjusted
20	Chen et al. 2023 (Microbiol Spectr)	Not reported (shares its cohort with [18])	Not reported	Hypertension defined on blood-pressure values alone; medication does not appear in the case	6. Not reported

				definition, the exclusion criteria or the analysis	
25	Verhaar et al. 2020	21.7% overall (1,016/4,672); 8.1% to 33.4% across the six ethnic groups	Participants asked to bring all current medication, from which use was determined	Entered as a binary covariate in the fully adjusted model alongside BMI, smoking and diabetes. The authors report that effects were attenuated after this adjustment but remained significant for most predictors	3. Adjusted as a single covariate
27	Louca et al. 2021	Not reported	Self-reported; listed among the variables available for analysis	Cases may be treated (current antihypertensive use is part of the case definition); controls required to be free of blood-pressure-lowering medication in both age strata. Drug use recorded but not entered as a covariate (models adjust for age, age^2 and BMI)	4. Mixed, not adjusted
31	Guo et al. 2026	Not reported	Structured clinical data record form recording medication use	Treated individuals explicitly included in the case definition (“even if current blood pressure < 140/90 mmHg”). Proportion not reported and no adjustment applied; the authors note the study was underpowered to examine the effect of specific antihypertensive medications	6. Not reported
32	Liu et al. 2026	92.9% (Daqing) and 84.4% (Haikou); daily use 78.6% and 75.0%	Self-reported frequency of use, tabulated in Table 1	The only study in the corpus to report frequency rather than a binary status. Compared between the two hypertensive groups (P = 0.524) but not adjusted for; covariates were age, BMI, smoking and alcohol. Both groups are hypertensive, so this is a within-hypertensive geographic comparison	5. Entire hypertensive group treated
33	Valdez-Palomares et al. 2025	100%	Reported	Entire sample under antihypertensive treatment; the comparison is between controlled and uncontrolled hypertension, not between hypertensive and normotensive participants	5. Entire hypertensive group treated
34	Palmu et al. 2020	18.0% (1,253/6,953), reported by class: beta-blockers 10.3%, RAS inhibitors 8.2%, calcium-channel blockers 4.2%, diuretics 3.3%	Finnish national Drug Purchase Register, which captures all prescription purchases; use defined as a purchase in the four months preceding baseline, not self-report	The only study in the corpus to adjust for antihypertensive medication by pharmacological class, entering diuretics (ATC C03), beta-blockers (C07), calcium-channel blockers (C08) and renin-angiotensin-system inhibitors (C09) as separate terms; it is also the only study to report the treated proportion broken down by class. Analyses were additionally repeated in users and non-users of antihypertensive medication separately	2. Adjusted by drug class

35	Yeo et al. 2026	0% at baseline in the incident analysis by design	National Drug Reimbursement Register: special reimbursement code or at least three purchases of dihydropyridine calcium-channel blockers or thiazide diuretics (ATC codes), a class restriction the authors justify explicitly	Prevalent hypertension, including self-reported antihypertensive use and register-based diagnosis, is an exclusion criterion; medication purchases define the incident outcome. Covariates: age, sex, BMI, type 1 and 2 diabetes, healthy food choices, cardiovascular disease and smoking	1. Treatment-naive by design
36	Verhaar et al. 2026	Not reported	Not reported; the authors list inaccurate reporting of medication among the study's limitations	Antihypertensive use forms part of the case definition but does not appear among the covariates of either model (model 1: age; model 2: age, sex, BMI, smoking and alcohol use), verified from the full text	4. Mixed, not adjusted
37	Sun et al. 2019	83.6% of hypertensive participants (153/183), of whom 128 were controlled; only 30 hypertensive participants were untreated	Self-reported medication inventory	Entered as a covariate in Model 3 for the systolic blood pressure analysis, explicitly justified on the grounds that medications may alter the gut microbiota. A stratified analysis separating hypertension from medication use was considered and reported as underpowered, with the supporting numbers given	3. Adjusted as a single covariate
38	Dan et al. 2019	0%	Recruitment screening	Patients who had received antihypertensive therapy were excluded; the diagnostic criterion itself specifies blood pressure thresholds “in the absence of antihypertensive medications”. Propensity-score matching on diet, age, sex, BMI and waist-to-hip ratio	1. Treatment-naive by design
39	Lv et al. 2023	0%	Recruitment screening	Antihypertensive medication is an explicit exclusion criterion, justified by its capacity to modulate the gut microbiota. The authors list treatment-naive recruitment among the study’s strengths	1. Treatment-naive by design
41	Song et al. 2023	Not reported	Not reported	Antihypertensive use forms part of the case definition; covariates were age, sex and BMI, with no medication term	4. Mixed, not adjusted
42	Yan et al. 2017	Approximately 35%, reported as a composite figure	Self-reported, stated only in the Discussion and not tabulated	The reported proportion combines antihypertensive drugs with nutritional supplements (“antihypertensive drugs or specific nutritious supplementary”) and is therefore not decomposable. No adjustment; groups matched on age, sex and BMI	4. Mixed, not adjusted
43	Joishy et al. 2022	Not reported	Questionnaire item on use of medication, not specific to antihypertensives	Generic medicinal use entered as one of 15 fixed effects in the linear mixed models. Medication is therefore adjusted for, but as an	3. Adjusted as a single covariate

				undifferentiated variable rather than an antihypertensive-specific or class-level term	
44	Huart et al. 2019	Not reported	Not reported	Antihypertensive treatment forms part of the case definition; no medication term in the analysis	4. Mixed, not adjusted
45	de la Cuesta-Zuluaga et al. 2018	Not reported	Not reported	No multivariable model relating microbiota to blood pressure is fitted, so no adjustment for medication arises; blood pressure is a descriptive covariate of microbial co-abundance groups	6. Not reported
46	Cheng et al. 2025	100% (153/153); mean duration of hypertension 16.8 ± 7.9 years	Tabulated in Table 1	No adjustment. The authors identify this as a limitation and call for studies in drug-naive patients to disentangle the effects of hypertension from those of its treatment	5. Entire hypertensive group treated
47	Liu et al. 2025	Not reported anywhere in the report	Not reported	Eight confounders adjusted (age, sex, BMI, smoking, antibiotic use, diabetes, hypercholesterolaemia, obesity); antihypertensive medication is not among them, although antibiotic use is	4. Mixed, not adjusted
48	Mushtaq et al. 2019	Not reported	Not reported	Grade 3 hypertension by World Health Organization classification; antihypertensive treatment is not mentioned in the eligibility criteria, the participant description or the analysis	6. Not reported
49	Zuo et al. 2019	0%	Recruitment screening	“None of the participants were under antihypertensive treatment”, verified across angiotensin-converting-enzyme inhibitors, angiotensin-receptor blockers, beta-blockers, statins and aspirin, with the exclusion justified by their potential influence on the gut microbiota	1. Treatment-naive by design
50	Silveira-Nunes et al. 2020	100%, on therapy for more than ten years	Reported by drug name and class	Inclusion required “reported and treated hypertension for more than ten years”, with chronic medication use that kept blood pressure under control. Classes named individually (thiazide and loop diuretics, angiotensin-receptor antagonists, beta-blockers), some participants on combinations. No adjustment	5. Entire hypertensive group treated
51	Qu et al. 2022	0%	Eligibility screening	Use of antihypertensive drugs is an exclusion criterion in the published inclusion criteria (the printed inequality is inverted in the source, but the intent is unambiguous)	1. Treatment-naive by design
57	Lin et al. 2025	Not reported	Not reported	Antidiabetic and antilipidaemic medication are adjusted for in the	4. Mixed, not adjusted

primary directed-acyclic-graph-based model, but antihypertensive medication is not

Categories. 1. Treatment-naive by design: treated participants excluded, or the analysis restricted to participants free of medication at baseline. 2. Adjusted by drug class: medication entered as separate pharmacological-class terms. 3. Adjusted as a single covariate: medication entered as one undifferentiated term. 4. Mixed, not adjusted: treated and untreated participants both present, with no medication term in the analysis. 5. Entire hypertensive group treated: every case on therapy, so the contrast is with treated rather than untreated hypertension. 6. Not reported: treatment status not stated. Counts across the 30 observational studies: category 1, n = 7; category 2, n = 1; category 3, n = 3; category 4, n = 9; category 5, n = 5; category 6, n = 5. Gao et al. [21] is retained as a narrative benchmark and is therefore not listed. Li et al. [5] is a Mendelian randomisation study with no participant-level medication data.

Checklist S1. PRISMA 2020 checklist

From: Page MJ, McKenzie JE, Bossuyt PM, Boutron I, Hoffmann TC, Mulrow CD, et al. The PRISMA 2020 statement: an updated guideline for reporting systematic reviews. BMJ 2021;372:n71. Reproduced under CC BY 4.0.

Section and topic	Item	Checklist item	Location where reported
**TITLE**			
**Title**	1	Identify the report as a systematic review.	Title page
**ABSTRACT**			
**Abstract**	2	See the PRISMA 2020 for Abstracts checklist.	Abstract
**INTRODUCTION**			
**Rationale**	3	Describe the rationale for the review in the context of existing knowledge.	Section 1
**Objectives**	4	Provide an explicit statement of the objective(s) or question(s) the review addresses.	Section 1, final paragraph
**METHODS**			
**Eligibility criteria**	5	Specify the inclusion and exclusion criteria for the review and how studies were grouped for the syntheses.	Section 2, paragraphs 2 and 6
**Information sources**	6	Specify all databases, registers, websites, organisations, reference lists and other sources searched or consulted to identify studies. Specify the date when each source was last searched or consulted.	Section 2, paragraph 3; Table S1
**Search strategy**	7	Present the full search strategies for all databases, registers and websites, including any filters and limits used.	Table S1
**Selection process**	8	Specify the methods used to decide whether a study met the inclusion criteria of the review, including how many reviewers screened each record and each report retrieved, whether they worked independently, and if applicable, details of automation tools used in the process.	Section 2, paragraph 3; Figure S1
**Data collection process**	9	Specify the methods used to collect data from reports, including how many reviewers collected data from each report, whether they worked independently, any processes for obtaining or confirming data from study investigators, and if applicable, details of automation tools used in the process.	Section 2, paragraph 4; Form S1
**Data items**	10a	List and define all outcomes for which data were sought. Specify whether all results that were compatible with each outcome domain in each study were sought, and if not, the methods used to decide which results to collect.	Section 2, paragraph 2; Form S1
	10b	List and define all other variables for which data were sought. Describe any assumptions made about any missing or unclear information.	Section 2, paragraph 4; Form S1
**Study risk of bias assessment**	11	Specify the methods used to assess risk of bias in the included studies, including details of the tool(s) used, how many reviewers assessed each study and whether they worked independently, and if applicable, details of automation tools used in the process.	Section 2, paragraph 5; Table S2

Section and topic	Item	Checklist item	Location where reported
**Effect measures**	12	Specify for each outcome the effect measure(s) used in the synthesis or presentation of results.	Section 2, paragraph 6
**Synthesis methods**	13a	Describe the processes used to decide which studies were eligible for each synthesis.	Section 2, paragraph 6
	13b	Describe any methods required to prepare the data for presentation or synthesis, such as handling of missing summary statistics, or data conversions.	*To complete*
	13c	Describe any methods used to tabulate or visually display results of individual studies and syntheses.	Section 2, paragraph 6; Tables 2–4
	13d	Describe any methods used to synthesise results and provide a rationale for the choice(s). If meta-analysis was performed, describe the model(s), method(s) to identify the presence and extent of statistical heterogeneity, and software package(s) used.	Section 2, paragraphs 6 and 7
	13e	Describe any methods used to explore possible causes of heterogeneity among study results.	Section 2, paragraph 6 (sex, ethnicity, compartment); Table S3
	13f	Describe any sensitivity analyses conducted to assess robustness of the synthesised results.	
**Reporting bias assessment**	14	Describe any methods used to assess risk of bias due to missing results in a synthesis (arising from reporting biases).	
**Certainty assessment**	15	Describe any methods used to assess certainty (or confidence) in the body of evidence for an outcome.	Section 2, final paragraph
**RESULTS**			
**Study selection**	16a	Describe the results of the search and selection process, from the number of records identified in the search to the number of studies included in the review, ideally using a flow diagram.	Figure S1
	16b	Cite studies that might appear to meet the inclusion criteria, but which were excluded, and explain why they were excluded.	Sections 3.1.7, 3.2.8; Figure S1
**Study characteristics**	17	Cite each included study and present its characteristics.	Section 3, overview table; Table S3
**Risk of bias in studies**	18	Present assessments of risk of bias for each included study.	Table S2
**Results of individual studies**	19	For all outcomes, present, for each study: (a) summary statistics for each group (where appropriate) and (b) an effect estimate and its precision, ideally using structured tables or plots.	Sections 3.1.2–3.1.4, 3.2.2–3.2.5, 3.3.2–3.3.5
**Results of syntheses**	20a	For each synthesis, briefly summarise the characteristics and risk of bias among contributing studies.	Sections 3.1.1, 3.2.1, 3.3.1; Table 5
	20b	Present results of all statistical syntheses conducted. If meta-analysis was done, present for each the summary estimate and its precision and measures of statistical heterogeneity. If comparing groups, describe the direction of the effect.	Tables 2–4 (structured vote-counting on direction of effect; no pooling)
	20c	Present results of all investigations of possible causes of heterogeneity among study results.	Sections 3.2.7, 4.x (sex, ethnicity, geography)
	20d	Present results of all sensitivity analyses conducted to assess the robustness of the synthesised results.	
**Reporting biases**	21	Present assessments of risk of bias due to missing results for each synthesis assessed.	*see item 14*
**Certainty of evidence**	22	Present assessments of certainty (or confidence) in the body of evidence for each outcome assessed.	Table 5
**DISCUSSION**			
**Discussion**	23a	Provide a general interpretation of the results in the context of other evidence.	Section 4

Section and topic	Item	Checklist item	Location where reported
	23b	Discuss any limitations of the evidence included in the review.	Section 4
	23c	Discuss any limitations of the review processes used.	
	23d	Discuss implications of the results for practice, policy, and future research.	Section 4, final paragraphs; Section 5
**OTHER INFORMATION**			
**Registration and protocol**	24a	Provide registration information for the review, including register name and registration number, or state that the review was not registered.	*n.a.*
	24b	Indicate where the review protocol can be accessed, or state that a protocol was not prepared.	n.a
	24c	Describe and explain any amendments to information provided at registration or in the protocol.	n.a
**Support**	25	Describe sources of financial or non-financial support for the review, and the role of the funders or sponsors.	Funding statement
**Competing interests**	26	Declare any competing interests of review authors.	Conflicts of Interest statement
**Availability of data, code and other materials**	27	Report which of the following are publicly available and where they can be found: template data collection forms; data extracted from included studies; data used for all analyses; analytic code; any other materials used in the review.	Data Availability Statement — currently reads “Not applicable” and should instead point to Form S1 and Table S3

Checklist S2. Synthesis Without Meta-analysis (SWiM) reporting checklist

From: Campbell M, McKenzie JE, Sowden A, Katikireddi SV, Brennan SE, Ellis S, et al. Synthesis without meta-analysis (SWiM) in systematic reviews: reporting guideline. BMJ 2020;368:l6890. Reproduced under CC BY 4.0. The SWiM items complement, and do not replace, the PRISMA 2020 items.

Item	Item name	What the guideline asks for	How it is addressed in this review
1	**Grouping studies for synthesis**	Describe how studies were grouped for each synthesis and provide a rationale.	Studies were grouped by anatomical compartment (gut bacteriome, oral bacteriome, mycobiome) and, within compartment, by type of result (diversity, taxon-level, functional/metabolic, causal). Section 2, paragraph 6.
2	**Describe the standardised metric and transformation methods used**	Describe the standardised metric for each outcome and the methods used for any data transformation.	No standardised metric was derived and no transformation was applied. Diversity estimates are reported in the metric of the original report (OR, HR, β), and taxon-level results as direction of effect only. Section 2, paragraphs 6–7.
3	**Describe the synthesis method**	Describe and justify the methods used to synthesise the effects.	Structured vote-counting on direction of effect, aggregated at phylogenetic family level; a taxon was considered convergent when reported in a concordant direction by at least two independent studies not sharing a cohort. Section 2, paragraph 6.
4	**Criteria used to prioritise results for summary and synthesis**	State and justify the criteria used to prioritise results.	Priority given to studies with an extractable effect estimate, correction for multiple comparisons, and a normotensive comparator; within-category comparisons were retained but reported as dedicated sub-analyses. Sections 2, 3.1.7, 3.2.8.
5	**Investigation of heterogeneity in reported effects**	Describe and justify the methods used to investigate heterogeneity.	Heterogeneity was examined narratively by compartment, sex, ethnicity, geography and blood pressure ascertainment, distinguishing studies purposively designed for an axis from post-hoc subgroup analyses. Sections 2, 3.2.7; Table S3.
6	**Certainty of evidence**	Describe the methods used to assess certainty of the synthesis findings.	GRADE adapted for exposure–outcome association rather than intervention effect, applied per key convergent finding; all bodies of evidence started at low certainty. Section 2, final paragraph; Table 5.
7	**Data presentation methods**	Describe the graphical and tabular methods used to present the effects.	Structured convergence tables per compartment (Tables 2–4) and a GRADE certainty table (Table 5). No forest plots, as no pooling was performed.
8	**Reporting results**	For each comparison, report the synthesis findings, including the direction, magnitude and precision of effects where available.	Sections 3.1–3.4.
9	**Limitations of the synthesis**	Report the limitations of the synthesis methods used and/or the certainty of the findings.	Section 4 (limitations of vote-counting, multiplicity, medication confounding, geographic restriction) and Section 5.
